# Novel asymmetrical azines appending 1,3,4-thiadiazole sulfonamide: synthesis, molecular structure analyses, *in silico* ADME, and cytotoxic effect†

**DOI:** 10.1039/d3ra00123g

**Published:** 2023-04-03

**Authors:** Samir Bondock, Tallah Albarqi, Ibrahim A. Shaaban, Moaz M. Abdou

**Affiliations:** a Chemistry Department, Faculty of Science, King Khalid University 9004 Abha Saudi Arabia; b Chemistry Department, Faculty of Science, Mansoura University 35516 Mansoura Egypt bondock@mans.edu.eg; c Department of Chemistry, Faculty of Science (Men's Campus), Al-Azhar University Nasr City 11884 Cairo Egypt; d Egyptian Petroleum Research Institute Nasr City 11727 Cairo Egypt

## Abstract

Toward finding potential and novel anticancer agents, we designed and prepared novel differently substituted unsymmetrical azine-modified thiadiazole sulfonamide derivatives using the “combi-targeting approach”. An efficient procedure for synthesizing the designed compounds starts with 5-acetyl-3-*N*-(4-sulfamoylphenyl)-2-imino-1,3,4-thiadi-azoline 4. The *E*/*Z* configuration for compound 5 was investigated based on spectral analysis combined with quantum mechanical calculation applying the DFT-B3LYP method and 6-31G(d) basis set. The computational results found that the *E* isomer was energetically more favorable than the *Z* isomer by 2.21 kcal mol^−1^. Moreover, ^1^H and ^13^C chemical shifts for the *E* and *Z* isomers in DMSO were predicted using the GIAO-B3LYP/6-31G(d) computations and IEF-PCM solvation model. The computed chemical shifts for both isomers are consistent with those observed experimentally, indicating that they exist in the solution phase. Moreover, the *E*/*Z* configuration for the synthesized azines 7a–c, 9, 11, 13, 15a and 15b was also studied theoretically using the DFT-B3LYP/6-31G(d) calculations. *In silico* prediction for the biological activities was reported regarding the HOMO–LUMO energy gaps and molecular reactivity descriptors besides the ADMT/drug-likeness properties. The cytotoxic effect of the synthesized compounds has been assayed *via* the determination of their IC_50_.

## Abstract 2

Toward finding potential and novel anticancer agents, we designed and prepared novel differently substituted unsymmetrical azine-modified thiadiazole sulfonamide derivatives using the “combi-targeting approach”.

## Graphical abstract

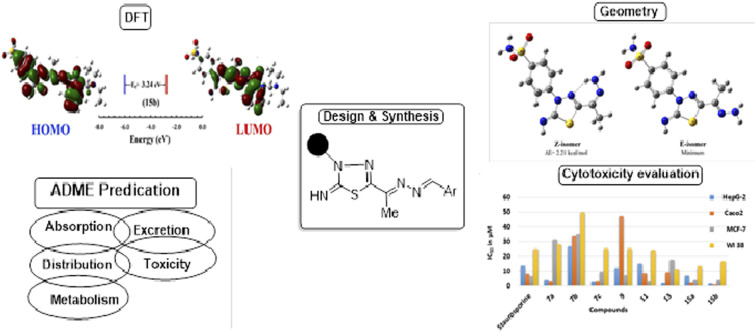

## 1. Introduction

Current obstacles to treating cancer include the emergence of drug resistance and unfavorable off-target effects of anticancer drugs, which energizes medicinal chemists to continuously produce novel anticancer medications with high efficacy and low toxicity.^1,2^

**Fig. 1:**
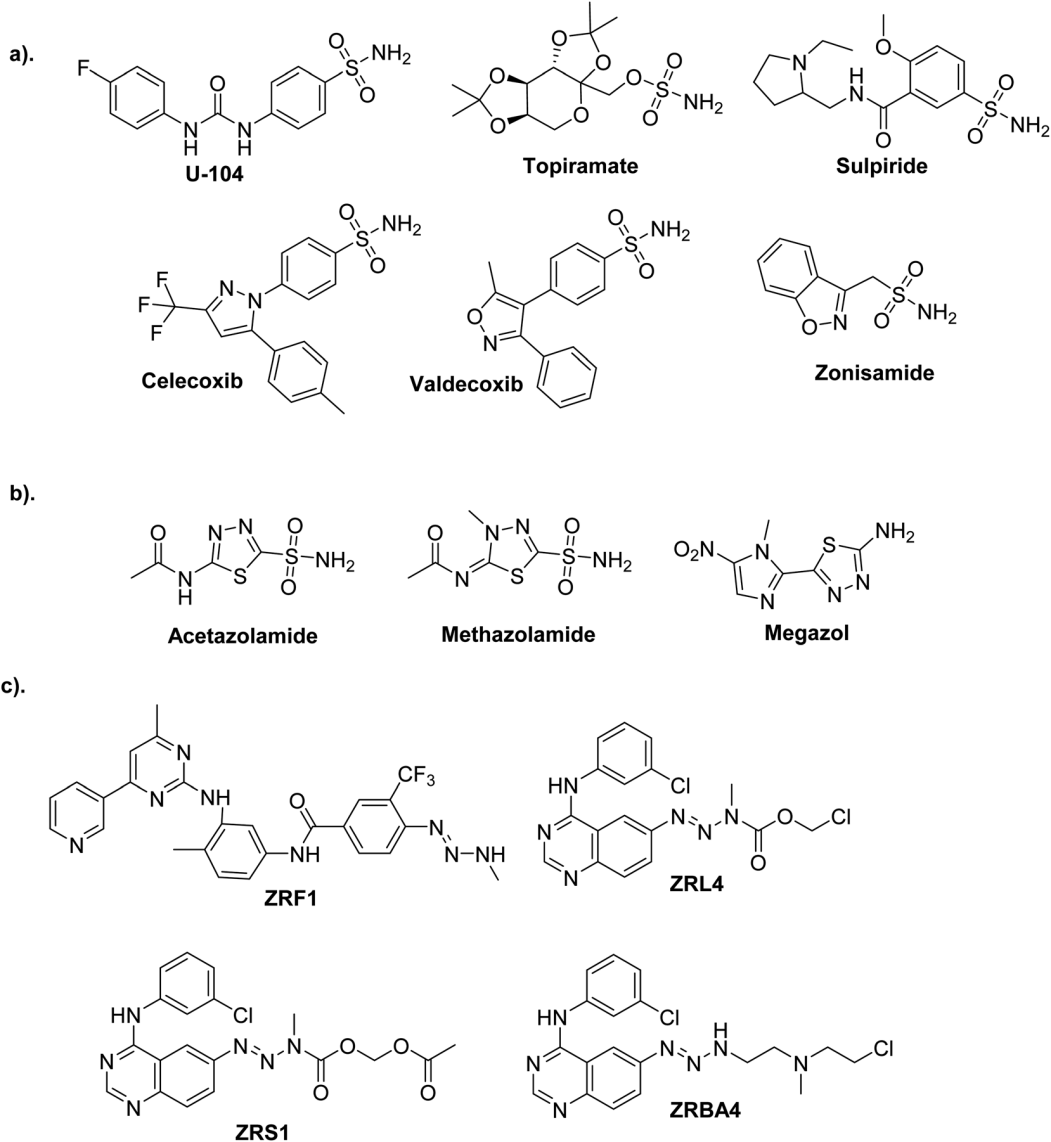
Commercial drugs on the market contain bioactive cores. (a) Sulfonamides. (b) Thiadiazole sulfonamides. (c) Asymmetrical azines.

One of the most promising candidates in the field of synthetic drugs is sulphonamides (Fig. 1a).^3–6^ The thiadiazole platform constitutes intriguing and rapidly expanding sulfonamide derivative systems. Several medicines on the market are related to this system, making it a flexible tool for drug design (Fig. 1b).^7,8^ According to a literature survey, azines and derivatives serve as crucial structural components in various versatile scaffolds with a wide range of drug applications (Fig. 1c).^9^ Azines have recently gained attention for configurations, and tautomers that profoundly affect biochemical processes.^10^

Recently, a vital approach/strategy in drug discovery is the amalgamation of two or more complete medications into a single molecular structure, known as a combi-molecule, which may be a good solution to achieve bioactive molecules, with high potency and different mechanisms of action, due to the synergistic effect.^11^ Hence, cohesive systems incorporating 1,3,4-thiadiazole sulfonamide with azines may help design new anticancer hybrids to improve biological properties.

To investigate the application of a combi-molecule strategy, we designed and prepared novel compounds 7a–c, 9, 11, 13 and 15a–b in this work by linking azines fragments and thiadiazole sulfonamides. These compounds were then subjected to cytotoxic assays on three cancerous cell lines. Their cytotoxic assay was comparable to the positive control staurosporine in the low micromolar region.

Moreover, we employ quantum mechanical (QM) computations to provide theoretical analyses for compound 5, including the *E*/*Z* configurations and conformational study toward free rotatable single bonds. Thus, QM calculations were reported using the density functional theory (DFT) at the level of B3LYP^12^ and 6-31G(d) basis set. Owing to its reliable accuracy and reduced computation time, the B3LYP/6-31G(d) calculation was widely recognized and applied for theoretical studies of organic molecules of medium-large size.^13,14^ The ^1^H and ^13^C NMR chemical shifts (*δ*, ppm) were also computed by applying the approach of gauge-invariant atomic orbitals, GIAOs,^15^ to investigate the *E*/*Z* configuration of 5 in the solution phase. *In silico* techniques have been widely applied to drug screening.^16–19^ Various computational tools and methods may be used to identify the candidate drug from other compounds depending on multiple features such as physicochemical/pharmacokinetic parameters and drug-likeness. Herein, QM calculations were carried out for the synthesized compounds to correlate their structures with biological activities *via* analysis of the FMOs and quantum chemical descriptors. To assess the synthesized compounds 7a–c, 9, 11, 13, 15a and 15b as drug candidates, the SwissADME^20^ and pkCSM^21^ servers were used to predict physicochemical characteristics, drug-likeness, and ADMET properties.

## 2. Results and discussion

### 2.1. Chemistry

**Scheme 1:**
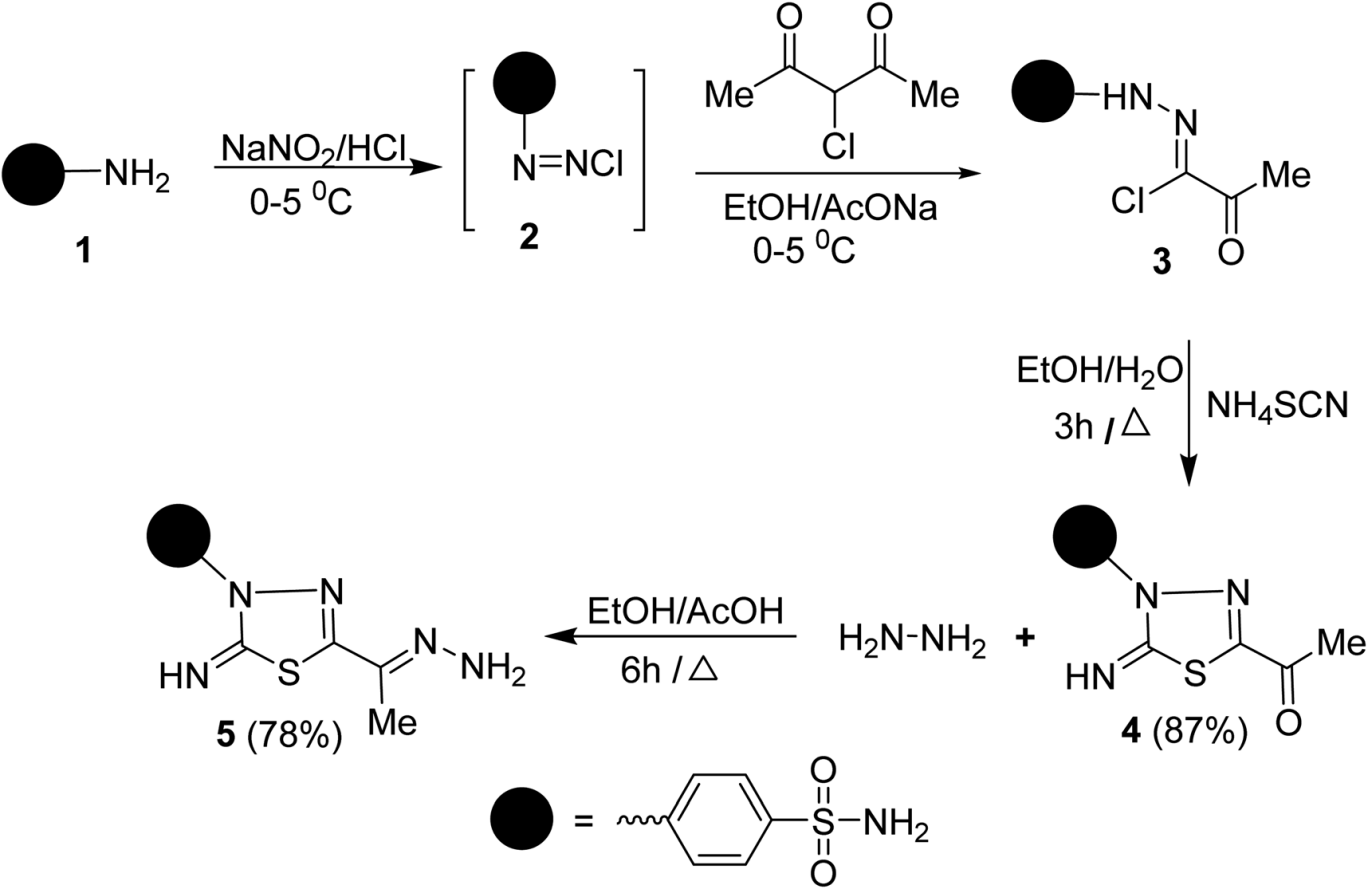
Synthesis of compound 5.

To synthesize the target compounds 7a–c, 9, 11, 13, 15a and 15b the synthetic sequence starts with the preparation of 5-acetyl-3-*N*-(4-sulfamoylphenyl)-2-imino-1,3,4-thiadiazoline 4 as commencing material. 1,3,4-Thiadiazoline 4 was efficiently prepared *via* a cyclization reaction of freshly synthesized 2-oxo-*N*-(4-sulfamoylphenyl)propanehydrazonoyl chloride 3, obtained from the Japp–Klingemann reaction of 3-chloro-2,4-pentanedione with diazonium chloride of sulfanilamide 2 in a buffered ethanolic solution, with an aqueous ethanolic solution of ammonium thiocyanate under reflux (Scheme 1). Compound 4's structure was determined *via* microanalysis and spectral data. In the IR spectrum, three bands were observed at 3352, 3289, and 3277 cm^−1^, indicating the existence of primary and secondary N–H stretching vibration, respectively. The bands at 1692, 1330, and 1298 cm^−1^ indicated the existence of a carbonyl group (C=O) and sulfonamide group (SO_2_NH_2_), respectively. The ^1^H-NMR spectrum displayed three singlet signals at *δ* 2.50, 7.45, and 9.62 ppm, characteristic for CH_3_, SONH_2_, and NH protons, respectively, as well as two doublet signals, resonating at *δ* 7.92 and 8.18 ppm with the identical coupling constant value (*J* = 9.35 Hz) and integrating for four protons indicating the existence of 4-disubstituted benzene. Its ^13^C-NMR spectrum showed the presence of eight signals which agrees with its molecular structure. The signals of CH_3_ and a carbonyl carbon resonate at 24.88 and 189.83 ppm, respectively. In the mass spectrum (MS), the molecular ion peak (M^+^) for 4 was found at *m*/*z* = 298, which is compatible with its molecular formula (C_10_H_10_N_4_O_3_S_2_).

**Fig. 2:**
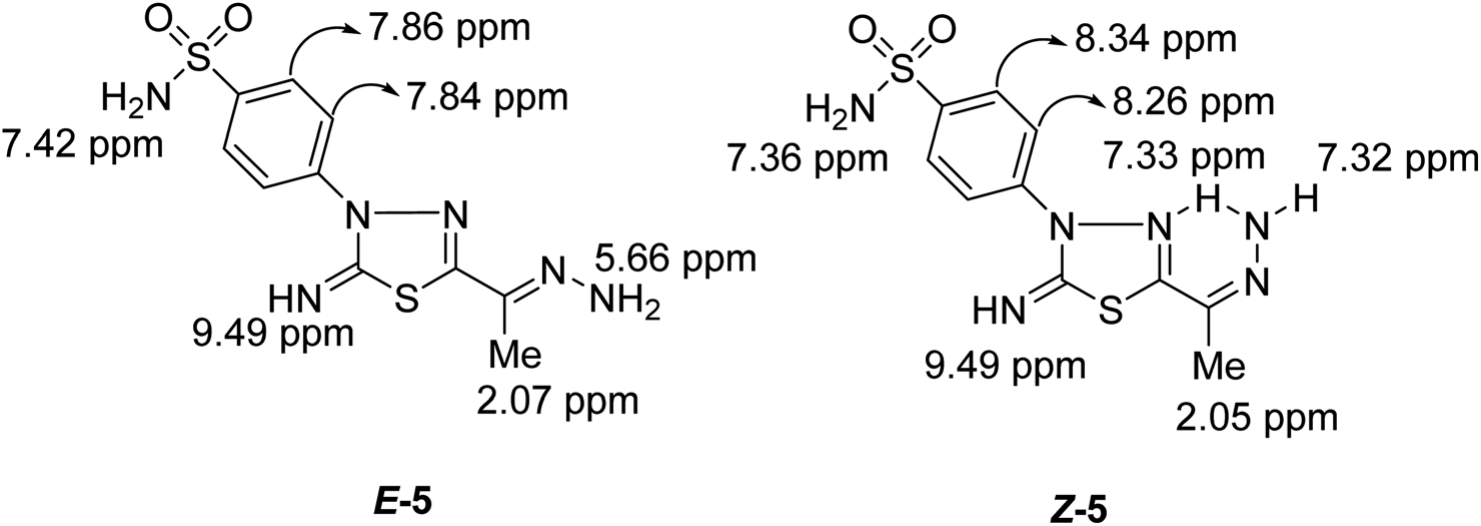
Protons chemical shift values of *E*- & *Z*-isomers of compound 5.

Condensation of 5-acetyl-3-*N*-(4-sulfamoylphenyl)-2-imino-1,3,4-thiadiazoline 4 with hydrazine afford the respective 4-(5-(1-hydrazonoethyl)-2-imino-1,3,4-thiadiazol-3(2*H*)-yl)benzenesulfonamide 5 (Scheme 1). The structure of later hydrazone 5 was confirmed through spectroscopic analyses. An examination of its IR spectrum revealed the lack of a carbonyl absorption band and the existence of azomethine (C=N) and amino groups at wavenumbers 1645, 3416, and 3285 cm^−1^. Interestingly, the ^1^H-NMR spectrum of hydrazone 5 showed two sets of resonances that supported the presence of 5 in two isomeric forms. The separation of signals of aromatic, –NH_2_, and CH_3_ protons in the two isomers is well resolved. The population ratio of the major and minor isomers is (52 : 48). Based on previous studies, the most stable and the major isomer is assigned to the *E*-isomer around the C=N bond.^22^ That is supported by the observed chemical shift value of the hydrazone-NH_2_ protons in *E*- and *Z*-isomers. In *E*-isomer, the hydrazone-NH_2_ protons appear as a singlet signal at *δ* 5.66 ppm. In comparison, *Z*-isomer resonates as two separate singlet signals at 7.33 and 7.32 ppm due to the possible formation of intramolecular H-bonds between N–H proton and C=N group in the thiadiazole ring as shown in Fig. 2. According to MS, the M^+^ at *m*/*z* is the molecular weight.

**Scheme 2:**
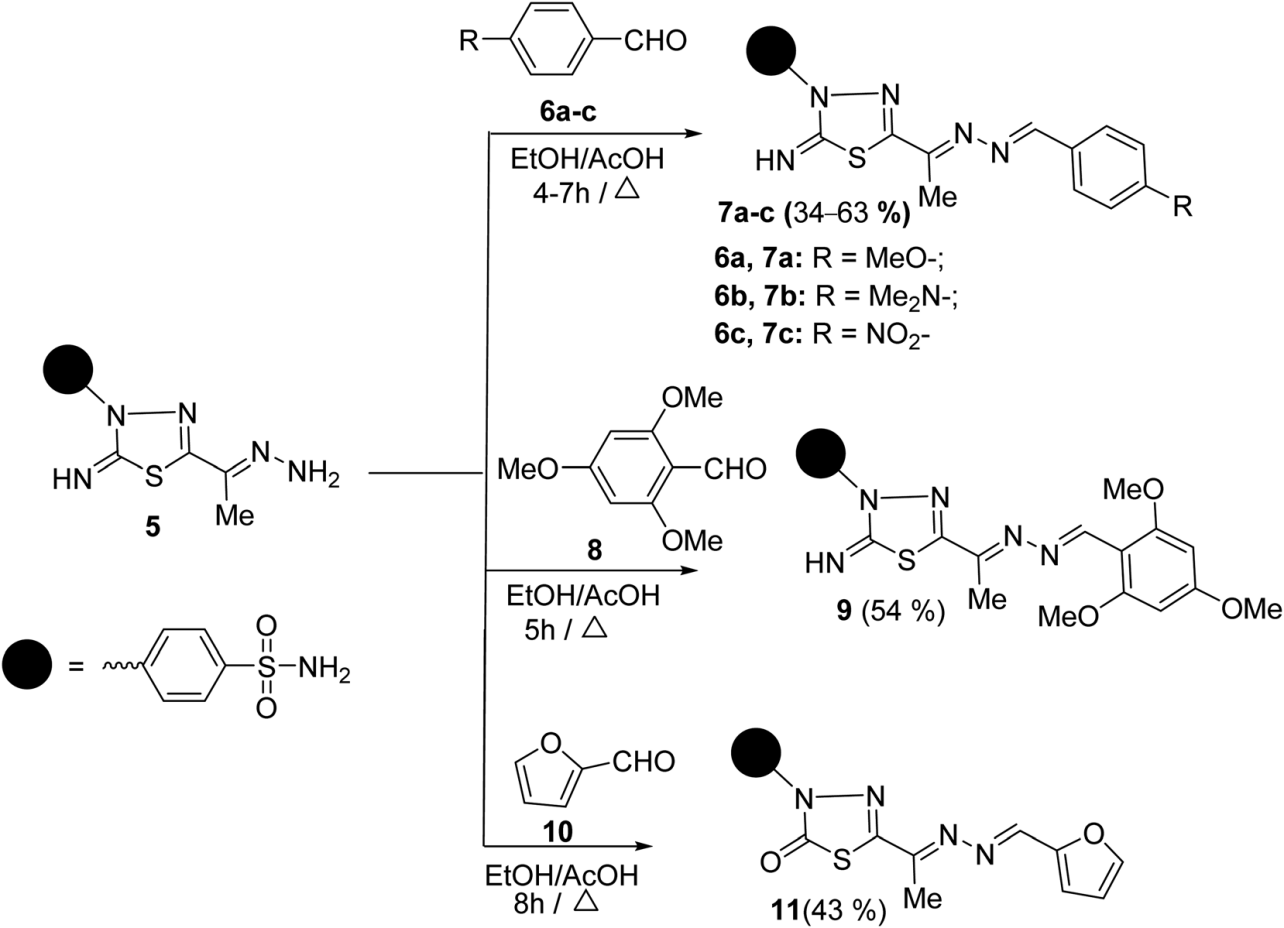
The synthesis of unsymmetrical azines 7a–c, 9, 11.

To synthesize unsymmetrical azines, we investigated the reactivity of the terminal –NH_2_ group of hydrazone 5 towards aromatic aldehydes, heterocyclic aldehydes, and cyclic ketones (Scheme 2). Hence, condensation of compound 5 with aromatic aldehydes, namely, 4-methoxy benzaldehyde 6a, 4-(*N*,*N*-dimethylamino) benzaldehyde 6b, 4-nitrobenzaldehyde 6c and 2,4,6-trimethoxybenzaldehyde 8 in refluxing ethanol yielded unsymmetrical azines, 1-(4-substituted benzylidene)-2-(1-(2-imino-3-(4-sulfamoylphenyl)-1,3,4-thiadiazol-5-yl)ethylidene) hydrazine 7a–c and 1-(2,4,6-trimethoxy benzylidene)-2-(1-(2-imino-3-(4-sulfamoyl phenyl)-1,3,4-thiadiazol-5-yl)ethylidene)hydrazine 9, respectively (Scheme 2). The molecular structures of the later azines were secured from microanalyses and spectral data. As a representative example, the IR spectrum of azine 7a displayed a strong absorption band at 3360, 3255, 3110, and 1604 cm^−1^ due to NH_2_, NH, and C=N functions, respectively. The ^1^H-NMR spectrum of 7a exhibited five characteristic singlet signals at *δ* 2.49, 3.84, 7.49, 8.63, 9.42 ppm corresponding to CH_3_, CH_3_O, NH_2_, azomethine proton (CH=N), and imine (C=NH) protons, respectively. Moreover, the ^13^C-NMR spectrum of 7a displayed fourteen carbon signals. Four characteristic peaks for four azomethine carbons showed at *δ* 161.91 (CH=N), 162.74 (C=N), 162.89 (thiadiazole-C_5_), and 168.75 (thiadiazole-C_2_) ppm. The signals at *δ* 13.46 and 55.85 ppm were attributed to methyl and methoxy carbons, respectively.

A M^+^ at *m*/*z* 430 is also revealed in the mass spectra of 7a, which corresponds to its molecular formula (C_18_H_18_N_6_O_3_S_2_). The mass fragmentation pattern of compound 7a (Scheme 1 at ESI†). The M^+^ undergoes N–N bond cleavages to give a cation ion peak at *m*/*z* = 134 a.m.u. followed by loss of hydrogen atom to afford ion peak at *m*/*z* = 133 a.m.u. Due to the formation of *p*-methoxybenzonitrile. Then, due to phenyl cation, it loses further methoxy and nitrile groups to afford the base peak at *m*/*z* = 77. The parent ion peak can also undergo denitrogenation with the formation of ion peaks at *m*/*z* = 402 and 403 a.m.u. assignable to 4-(2-imino-5-(1-(4-methoxyphenyl)prop-1-en-2-yl)-1,3,4-thiadiazol-3(2*H*)-yl)benzenesulfonamide and its protonated form, respectively (Scheme 1 at ESI†).

Similarly, the treatment of aminohydrazone 5 with furfural 10, as heterocyclic aldehyde, in ethanol afforded 1-(furan-2-ylmethylene)-2-(1-(2-oxo-3-(4-sulfamoylphenyl)-1,3,4-thiadiazol -5-yl) ethylidene)hydrazine 11 (Scheme 2). The IR spectrum of 11 exhibited bands at 3338–3245, 1685, 1625, 1325, and 1291 cm^−1^, which were attributed to NH_2_, C=O, C=N, and SO_2_ functions, respectively. Furthermore, the MS of 11 showed a M^+^ at *m*/*z* = 391 and an ion peak at *m*/*z* = 393 (M^+^ + 2). The ^1^H-NMR spectrum of 11 revealed the absence of imine proton at about 9.50 ppm. It disclosed the existence of three singlet signals at *δ* 2.44, 7.47, 8.48 ppm assigned to CH_3_, NH_2_, and CH=N protons, along with the expected signals of furan and benzene residue. Its ^13^C-NMR spectrum displayed important signals resonating at *δ* 13.68, 159.01, and 168.29 ppm characteristics for CH_3_, CH=N, and C=O carbons, respectively.

**Scheme 3:**
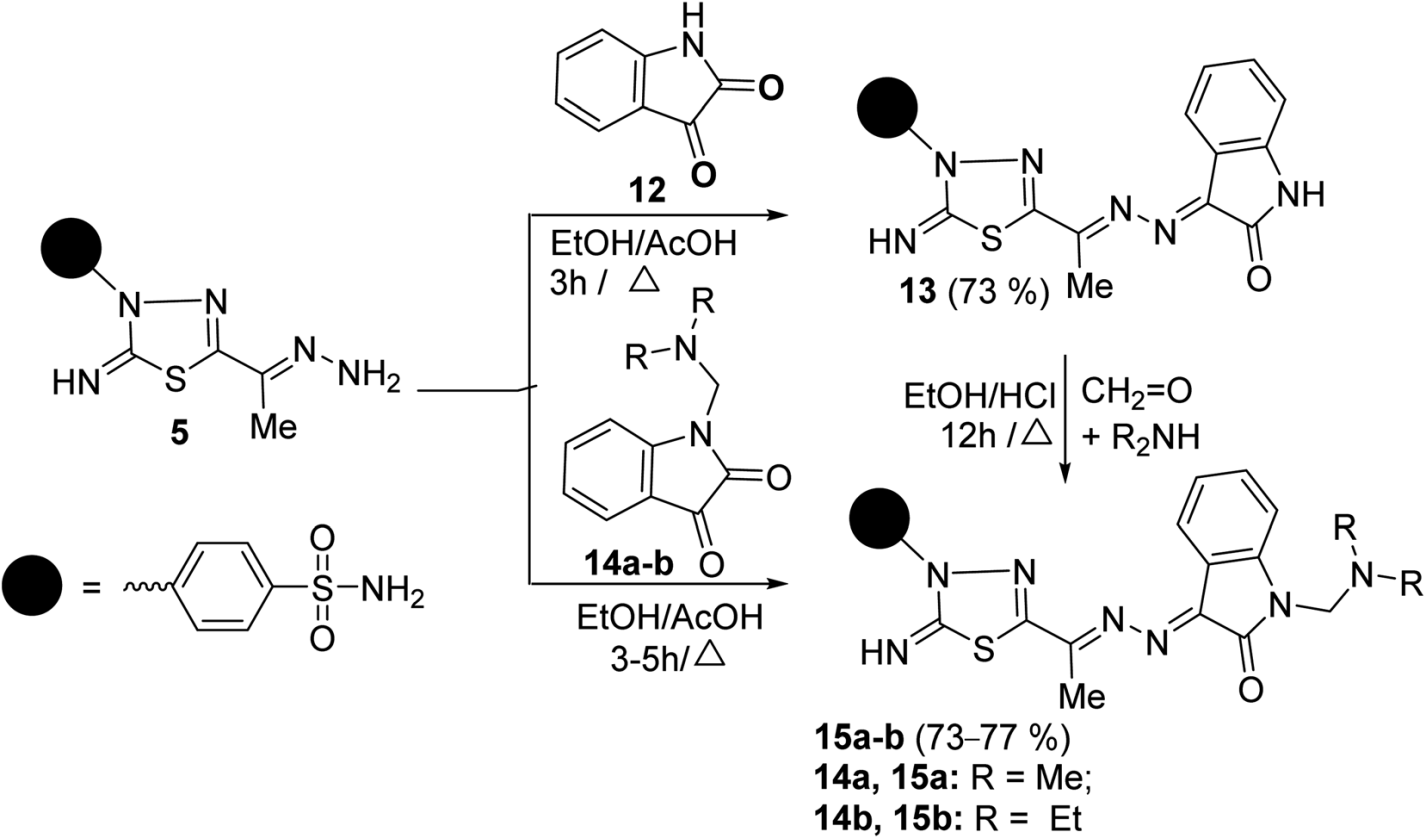
Reaction of aminohydrazone 5 with heterocyclic ketones 12, 14a–b.

The scope of these condensation reactions was extended *via* the treatment of aminohydrazone 2 with heterocyclic ketones, namely, isatin 12, 1-(*N*,*N*-dimethylaminomethyl)isatin 14a,^23^ and 1-(*N*,*N*-diethylaminomethyl)isatin 14b (ref. 23) to afford the respective unsymmetrical azines 13, 15a and 15b (Scheme 3). The MS of 13, 15a, and 15b showed parent ion peaks at *m*/*z* = 441, 498, and 526 a.m.u. which is consistent with their molecular formulas (C_18_H_15_N_7_O_3_S_2_), (C_21_H_22_N_8_O_3_S_2_) and (C_23_H_26_N_8_O_3_S_2_), respectively. The IR spectrum of 13 showed bands at 3303–3257, 3115, 1717, 1607, and 1587 characteristics for NH_2_, NH, C=O, C=N, and C=C functions, respectively. The ^1^H-NMR spectrum of 13 showed three D_2_O-exchangeable signals at *δ* 10.92, 9.54, 7.41 ppm assignable to indoline-NH, imine-NH, NH_2_ proton, and a singlet signal at 2.32 ppm assignable to the CH_3_ protons. Its ^13^C-NMR spectrum revealed signals at *δ* 163.91, 158.09, 154.71, and 13.49 ppm specific for carbonyl, thiadiazole-C_2_, C=N, and CH_3_ carbons, respectively. Additionally, the structures of 15a and 15b were chemically indicated *via* Mannich reactions of 13 with each formalin and dimethylamine or formalin and diethylamine.

### 2.2. Quantum mechanical study

#### 2.2.1. Molecular structure analyses

**Fig. 3:**
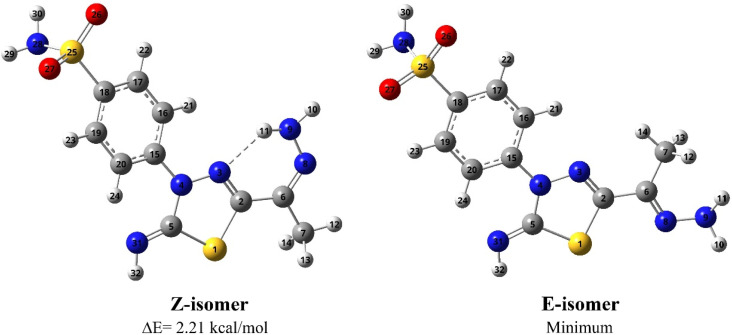
Optimized geometries and atom numbering for *Z* and *E* isomers of 5 predicted using the B3LYP/6-31G(d) calculations.

Initially, the *Z*/*E* configurations were theoretically investigated for 5 according to the orientation of amino N and thiadiazol C attached to the C=N bond where they are in the same (*Z* isomer) or opposite sides (*E* isomer), see Fig. 3. The computational outcomes using B3LYP functional combined with the 6-31G(d) basis set reveal that the *E* configuration is more stable than the *Z* form by 769 cm^−1^ (2.21 kcal mol^−1^). While adopting the *E*/*Z* configurations, we need to explore the exact conformation of –C(CH_3_)NNH_2_, phenyl sulfonamide, sulfonamide, and methyl moieties which result from free rotation about C_2_–C_6_, N_4_–C_15_, C_18_–S_25_ and C_6_–C_7_ single bonds, respectively. For this purpose, a relaxed scan of the PES was performed throughout the rotation of the dihedral angles *τ* N_8_C_6_C_2_N_3_, *τ* C_16_C_15_N_4_N_3_, *τ* N_28_S_25_C_18_C_19,_ and *τ* H_12_C_7_C_6_C_2_ from 0° to 360° in steps of 10° proceeded by optimization process after every scan point. The obtained curves of the PES scan using B3LYP/6-31G(d) calculations for *E* and *Z* isomers are given at ESI (Fig. S1 and S2†). For the *E* isomer, the predicted PES curve from the rotation of –C(CH_3_)NNH_2_ moiety shows a global minimum when *τ* N_8_C_6_C_2_N_3_ reaches 180.0° in which the imine bonds (C=N) are *trans* to each other.

In contrast, minimum energy for *Z*-isomer was obtained at *τ* N_8_C_6_C_2_N_3_ equal to 0.0° where both C=N bonds are *cis* to each other where stabilization could be attributed to intra-molecular H-bonding interaction between amino hydrogen and thiadiazol nitrogen. Moreover, the energetically favored conformation of benzenesulfonamide moiety (–C_6_H_4_SO_2_NH_2_) concerning the thiadiazol ring was assigned at *τ* C_16_C_15_N_4_N_3_ of 10.0° where the phenyl ring is almost planar towards the thiadiazol ring. The predicted curve for the scan of PES throughout the rotation of sulfonamide moiety for both *E* and *Z* isomer exhibit a minimum conformation at *τ* N_28_S_25_C_18_C_19_ equal to 260.0° in which the amino group is perpendicular to the phenyl ring. The internal rotation of the methyl group gives rise to a minimum at *τ* H_12_C_7_C_6_C_2_ equal to 60.0° and 0.0° for *Z* and *E* configuration, where the methyl group is orientated in *eclipsed* and *staggered* conformation to the adjacent C=N bond, respectively. In conclusion, opposite conformations of C(CH_3_)NNH_2_ and methyl moieties were obtained for *E* and *Z* isomers. In contrast to the *E* isomer, the global minimum of the *Z* isomer has the C=N bond in *cis* orientation to the thiadiazol C=N bond with the *eclipsed* conformation of the methyl group towards the C=N bond (Fig. 3).

The measured ^13^C NMR spectrum for 5 displays 20 signals, double the number of carbon atoms, and reveals the presence of both *Z* and *E* isomers in the solution phase. Thus, the ^1^H and ^13^C NMR chemical shifts (*δ* in ppm) were calculated for both isomers using B3LYP/6-31G(d) calculations and compared to those observed experimentally. The computed ^1^H and ^13^C chemical shifts for *Z* and *E* isomers have equivalent values in the experimental spectra, as Table 1 shows that both isomers exist in the solution phase. In contrast to the *E* isomer, one proton of amino-hydrazone moiety (H_11_) for the *Z* isomer was predicted to resonate at 7.31 ppm and matches the observed signal at 7.33 ppm, confirming the presence of the *Z* isomer in the solution phase. The downfield shift for H_11_ accounts for the intra-molecular H bonding with adjacent thiadiazol nitrogen. The ^1^H NMR spectrum shows three signals at 7.36, 7.36, and 7.43 ppm corresponding to *amino* protons of sulfonamide moiety (H_29_ and H_30_) in excellent agreement with those calculated for both *E* and *Z* isomers, 7.34–7.41 ppm. It's worth noting that, compared to tetramethylsilane (TMS), the prediction of chemical shifts for amino protons was improved when a multi-standard technique^24,25^ was applied using a comparable skeleton as a reference.

**Table 1:** Calculated^a^^1^H/^13^C NMR chemical shift (*δ*, ppm) for *Z* and *E* isomers of 5 compared to experimental values

Atom^b^	*δ*_calc_		*δ*_exp_ c	Atom^b^	*δ*_calc_		*δ*_exp_ c
	*Z*	*E*			*Z*	*E*	
C_2_	149.14		151.26	H_10_	5.52		(5.67)
		155.67	153.01			5.16	(5.67)
C_5_	160.48		(159.23)	H_11_	7.31		7.33
		164.53	(159.23)			5.63	5.67
C_6_	126.49		126.80	H_12_	1.95		(2.08)
		138.02	139.07			1.76	(2.08)
C_7_	20.14		11.36	H_13_	2.19		(2.08)
		7.64	10.27			1.68	(2.08)
C_15_	144.59		142.89	H_14_	2.21		(2.08)
		145.49	145.89			2.71	(2.08)
C_16_	121.93		122.43	H_21_	7.66		7.87
		120.39	120.24			7.79	8.28
C_17_	128.93		127.15	H_22_	7.73		8.27
		129.00	(131.35)			7.70	7.88
C_18_	138.60		141.75	H_23_	7.65		7.86
		136.97	134.63			7.60	7.84
C_19_	129.02		(131.35)	H_24_	8.57		8.35
		129.49	(131.35)			8.97	8.36
C_20_	120.83		121.33	H_29_	7.41		7.43
		118.09	118.60			7.36	(7.36)
				H_30_	7.38		(7.36)
						7.34	7.34
				H_32_	10.71		9.15
						10.82	9.31
rms^d^	3.24	2.41			0.49	0.57	
*R* ^2^ e	0.9988	0.9988			0.9838	0.9804	

a Calculations were carried out in DMSO solution by B3LYP/6-31G(d) method utilizing the IEF-PCM solvation model.

b For the structures and atom numbering of *Z* and *E* isomers (Fig. 3).

c The values of chemical shifts between brackets are assigned to more than one atom.

d rms refers to the root mean square deviations of the theoretical values of chemical shifts from their corresponding experimental values.

e The correlation coefficient (*R*^2^) between the computed and observed values of chemical shifts.

The predicted chemical shift for the *Z* isomer at 126.49 ppm belongs to the hydrazone carbon atom (C_6_) and better matches the ^13^C signal observed at 126.80 ppm. The counterpart value for the *E* isomer was computed at 138.02 ppm, which is consistent with the signal observed at 139.07 ppm. The experimental ^13^C NMR spectrum shows signals at 151.26, 141.75, 122.43 and 121.33 ppm are better correlated to the *Z* isomer's computed values of 149.14 (C_2_), 138.60 (C_18_), 121.93 (C_16_) and 120.83 (C_20_) ppm, respectively. Regarding the *E* isomers, these atoms are predicted to resonate at 155.67, 136.97, 120.39, and 118.09 ppm, respectively, which match the signals displayed at 153.01, 134.63, 120.24, and 118.60 ppm, respectively. Compared to the experimental value, the calculated chemical shifts of thiadiazol C_5_ (for *E* isomer) and methyl C_7_ (for *Z* isomer) were noticeably overestimated by 5.30 and 8.78 ppm, respectively. In sum, the computed chemical shifts of hydrogen/carbon nuclei for both *Z* and *E* isomers are well compared to those observed in the experimental ^1^H/^13^C NMR spectra, with high correlation coefficients (*R*^2^) of 0.9838/0.9988 and 0.9804/0.9988, respectively.

**Fig. 4:**
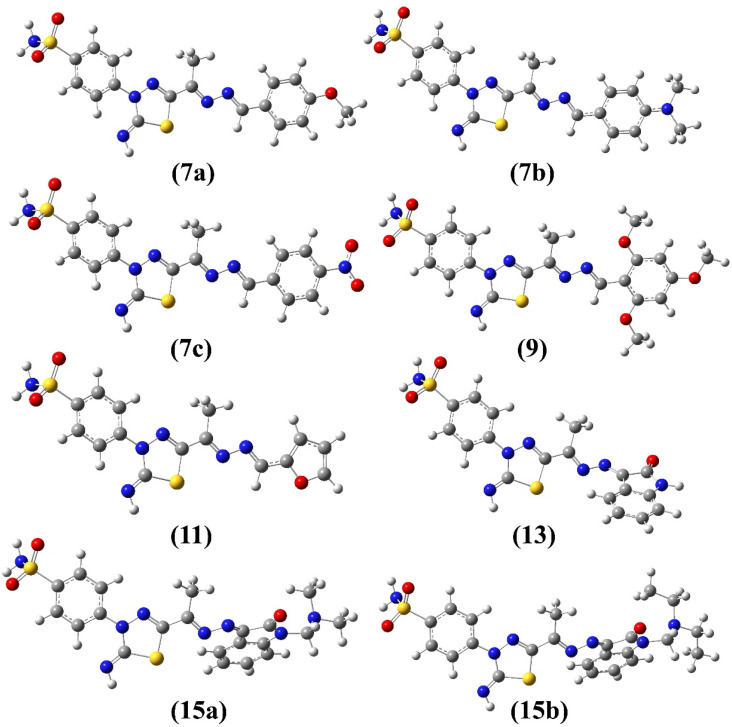
Optimized geometries of 7a–c, 9, 11, 13 and 15a–b obtained from B3LYP/6-31G(d) calculations.

The *E*/*Z* configuration for the synthesized products 7a–c, 9, 11, 13 and 15a–b were theoretically explored based on the orientation around the hydrazonyl C=N bonds, which results in four possible configurations, 1 (*EE*), 2 (*ZZ*), 3 (*EZ*) and 4 (*ZE*). Therefore, a full geometry optimization was carried out for each configuration followed by frequency applying B3LYP method at 6-31G(d) basis set. The ESI† provides the equilibrium geometries and computed energy difference for the suggested configurations (Fig. S3–S10/Table S1†). For all synthesized compounds, the computational outcomes revealed *EE* (1) isomer to be the favored configuration with the lowest energy. Fig. 4 shows the predicted equilibrium geometries for the most stable configuration (1, *EE*) for 7a–c, 9, 11, 13, 15a and 15b.

The impact of FMOs and their associated molecular reactivity descriptors on molecule biological reactivity has recently been considered.^26,27^ Herein, The FMOs were predicted for the optimized geometries of the synthesized molecules (5, 7a–c, 9, 11, 13 and 15a–b) to evaluate the reactivity and to correlate their biological activities. Subsequently, the computed energies for HOMO and LUMO were used to calculate a set of quantum chemical descriptors (Table 2), which are useful in assessing the molecule's overall reactivity.^28,29^

**Table 2:** DFT-B3LYP based^a^ quantum chemical descriptors^b^ for the synthesized compounds

Cpd	*E*	*I*	*A*	*E*_g_	*η*	*μ*	*ω*	*μ*_tot_	*α*
5	−45 266.30	5.75	1.64	4.11	2.06	−3.69	3.32	7.01	210.19
7a	−55 706.90	5.76	2.31	3.45	1.73	−4.03	4.71	9.53	367.89
7b	−56 236.08	5.36	2.11	3.24	1.62	−3.74	4.30	12.07	407.38
7c	−58 155.23	6.17	3.21	2.96	1.48	−4.69	7.43	3.69	368.79
9	−61 939.41	5.48	2.00	3.49	1.74	−3.74	4.01	11.91	402.22
11	−52 529.97	5.83	2.48	3.35	1.68	−4.15	5.15	7.73	321.64
13	−57 148.21	6.08	2.85	3.23	1.61	−4.46	6.17	5.25	339.36
15a	−61 863.38	6.02	2.76	3.25	1.63	−4.39	5.93	8.37	386.01
15b	−64 002.88	6.00	2.76	3.24	1.62	−4.38	5.92	8.77	409.20
Staurosporine	−41 561.54	4.90	0.79	4.12	2.06	−2.84	1.96	7.91	342.39
Valdecoxib	−36 773.65	6.60	1.34	5.25	2.63	−3.97	3.00	3.07	203.20
Methazolamide	−39 083.89	6.58	1.78	4.80	2.40	−4.18	3.64	1.50	126.35
ZRL4	−55 935.10	6.06	2.12	3.94	1.97	−4.09	4.25	2.18	279.79

a Calculations were carried out using 6-31G(d) basis set.

b *E*, total energy; *I*, ionization potential = −*E*_HOMO_; *A*, electron affinity = −*E*_LUMO_; *E*_g_, energy gap = *E*_LUMO_–*E*_HOMO_; *η*, hardness = (*I* − *A*)/2; *μ*, chemical potential = −(*I* + *A*)/2; *ω*, electrophilicity index = *μ*^2^/2*η* (all in eV); *μ*_tot_, total dipole moment in Debye; *α*, polarizability in a.u.

**Fig. 5:**
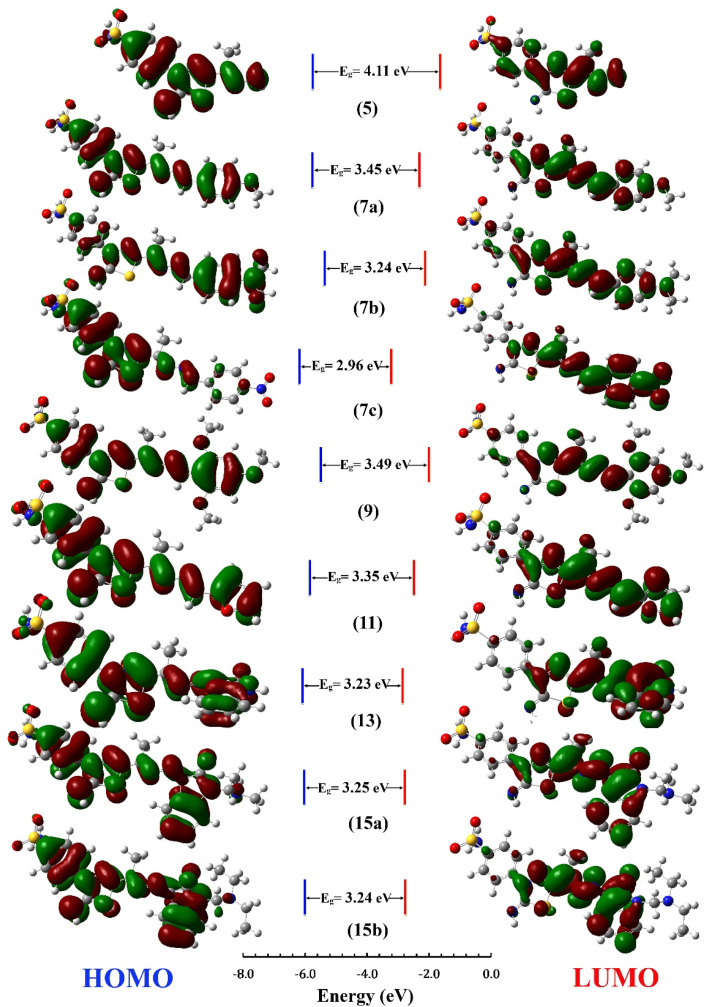
HOMO and LUMO Frontier MOs and energy gaps (*E*_g_) for the synthesized compounds predicted using B3LYP/6-31G(d) calculations.

The distribution for electron density in HOMOs and LUMOs for the investigated compounds is shown in Fig. 5, along with their energy gaps predicted using the B3LYP/6-31G(d) computations. The HOMO is largely localized on the thiadiazol ring and phenyl ring of the benzenesulfonamide moiety except for 7b, where the HOMO is mostly distributed over the dimethylphenylamine moiety. The LUMO localized over the whole molecule for all synthesized molecules except the region containing the benzenesulfonamide moiety.

The energy gap (*E*_HOMO_–*E*_LUMO_) is a valuable sign of a molecule's chemical reactivity and kinetic stability. A molecule with a small energy gap is more polarized and has a higher chemical reactivity and lower kinetic stability.^30^ Furthermore, Small energy gaps suggest that the molecule undergoes a large intramolecular charge transfer, which might affect the molecule's biological activity.^31^ The energy gaps calculated for the synthesized compounds ranged from 2.96 to 4.11 eV, comparable to the reported values for bioactive molecules^32–34^ and that computed for the standard reference drug, staurosporine (4.12 eV). For all compounds, the calculated energy gap *E*_g_ (Table 2) decreases in the order 5 (4.11 eV) > 9 > 7a > 11 > 15a > 15b = 7b > 13 > 7c (2.96 eV). These results are compatible with the observed high activities for 7c, 13, 15b, and 15a against HepG-2 and Caco2 (Table 3).

**Table 3:** Cytotoxicity (IC_50_) of tested compounds 7a–c, 9, 11, 13, 15a and 15b on different cell lines

Compound No.	IC_50_ (μM)			
	HepG-2	Caco2	MCF-7	WI 38
7a	4.17 ± 0.20	3.16 ± 0.20	31.50 ± 1.4	28.20 ± 1.50
7b	26.80 ± 1.50	33.60 ± 1.70	35.20 ± 1.6	49.80 ± 2.60
7c	3.04 ± 0.20	3.37 ± 0.20	9.59 ± 0.4	25.40 ± 1.90
9	12.20 ± 0.70	47.20 ± 2.30	7.21 ± 0.3	25.40 ± 1.30
11	15.00 ± 0.80	8.55 ± 0.40	3.34 ± 0.2	24.50 ± 1.80
13	1.76 ± 0.10	8.99 ± 0.40	17.70 ± 0.8	11.40 ± 0.60
15a	6.54 ± 0.40	2.33 ± 0.10	4.34 ± 0.2	13.40 ± 0.70
15b	1.62 ± 0.10	0.78 ± 0.04	4.43 ± 0.2	16.80 ± 0.90
Staurosporine	13.60 ± 0.80	8.18 ± 0.70	6.19 ± 0.3	25.20 ± 1.30

Chemical potential (*μ*) is the inverse of electronegativity (*χ*), and it defines how much energy a molecule absorbs or releases during a chemical process. Both descriptors significantly impact a molecule's inhibitory effectiveness.^35^ Compound 7c, with a high *χ* value (of 4.69 eV), is more active than 7a and 7b with low values (4.03 and 3.74 eV), which is attributed to the presence of the nitro group in 7c and validates the observed higher activity against MCF-7 (Table 3). Also, the hardness value for 7c (1.48 eV) is lower than that calculated for 7a and 7b. It shows that it is a softer and more reactive molecule compared to 7a and 7b, in agreement with the estimated energy gap, see Table 2. Owing to the values of the electrophilicity index (*ω*), organic compounds were categorized as strong (*ω* > 1.5 eV), moderate (0.8 < *ω* < 1.5 eV), or weak (*ω* < 0.8 eV) electrophiles.^36^ Compounds 7c and 13 are powerful electrophiles in this study, with *ω* values of 7.43 and 6.17, respectively. Compound 9 has the lowest biological activity against Caco2, which might be explained by its high energy gap (3.49 eV) and poor electrophilicity (4.01 eV) between all the synthesized compounds. To support the combi-targeting technique in this study, QM descriptors were computed for three commercial drugs with similar cores, valdecoxib, methazolamide, and ZSL4 (Fig. 1, Table 2). As a result, the computed *E*_g_ and *η* values for the ZSL4 drug matched those predicted for compound 5 and staurosporine. Also, compounds 7a and 11 have *μ* values of −4.03 and −3.97 eV, respectively, and are similar to those obtained for valdecoxib and ZSL4 drugs.

The dipole moment is also considered when correlating the molecule's biological activity, which might influence the degree of interaction between drugs and the active sites of protein.^37^ Compounds 15a–b, for example, are more active against Caco2 and MCF-7 than 13, which may be explained by the fact that 15a–b has a higher total dipole moment (8.37–8.77 Debye) than 13 (5.25). As shown in Table 2, compounds 7b and 9 have high total dipole moments (12.07 and 11.91 Debye, respectively) and exhibit high polarizabilities (407.38 and 402.22 a.u.), indicating that they are a good candidate for non-linear optical (NLO) materials.^38^

#### 2.2.2. ADMET prediction

Besides the high potency, the drug candidate's success involves favorable ADMET (absorption, distribution, metabolism, excretion, and toxicity) properties.^39^ The ADMET prediction models have been introduced as an additional tool to aid drug discovery.^16^ This study predicted the *in silico* ADMET characteristics of all synthesized compounds 7a–c, 9, 11, 13, 15a and 15b using the SwissADME^20^ and pkCSM^21^ servers, as shown in Table 4. The synthesized compounds show percent absorption ranged from 73.44 to 78.33%, suggesting they are well absorbed *via* the human intestine. Table 4 shows that three compounds 7a–c have no violations of Lipinski's rule of five^40^ for drug-likeness features and are considered orally active drugs. Other compounds have just one violation. All the synthesized compounds 7a–c, 9, 11, 13, 15a and 15b were found to be AMES nontoxic in nature and exhibit lethal doses (LD_50_) ranging from 2.02 to 3.32 mol kg^−1^, indicating these compounds seem to be suitably safe.

**Table 4:** Physicochemical and pharmacokinetic properties for the synthesized compounds 7a–c, 9, 11, 13 and 15a–b

Parameter^a^	7a	7b	7c	9	11	13	15a	15b	Staurosporine
MW (g mol^−1^)	430.50	443.55	445.48	490.56	390.44	441.49	498.58	526.63	466.53
*n*_rot_	6	6	6	8	5	4	6	8	2
HBA	8	7	9	10	8	8	9	9	4
HBD	2	2	2	2	2	3	2	2	2
TPSA (Å^2^)	172.40	166.41	208.99	190.86	176.31	192.27	186.72	186.72	69.45
%Abs	77.97	76.85	75.87	74.37	78.33	78.25	74.61	76.12	95.79
log *P*_o/w_	2.42	2.40	2.40	2.33	2.33	2.33	1.83	1.83	3.03
log *S*	−4.10	−4.26	−4.26	−4.25	−4.25	−4.25	−4.26	−4.26	−5.06
GI abs	Low	Low	Low	Low	Low	Low	Low	Low	High
log *K*_p_ (cm s^−1^)	−7.17	−7.15	−7.15	−7.58	−7.58	−7.58	−7.81	−7.81	−6.85
Lipinski, violation	Yes; 0	Yes; 0	Yes; 0	Yes; 1	Yes; 1	Yes; 1	Yes; 1	Yes; 1	Yes; 0
AMES toxicity	No	No	Yes	No	No	No	No	No	Yes
LD_50_ (mol kg^−1^)	2.14	2.24	3.32	2.20	2.18	2.02	2.13	2.21	2.46
Skin sensitization	No	No	No	No	No	No	No	No	No

a MW, molecular weight (g mol^−1^); *n*_rot_, number of rotatable bonds; HBA, number of H-bond acceptors; HBD, number of H-bond donors; TPSA, topological polar surface area; %Abs, intestinal absorption (human); log *P*_o/w_, lipophilicity; log *S*, water solubility; GI abs, gastrointestinal absorption; log *K*_p_, skin permeation, Lipinski, rule of five for drug likeness; LD_50_, oral rat acute toxicity.

The oral bioavailability was estimated using the SwissADME's bioavailability radar, which considers six physicochemical parameters: size, solubility, lipophilicity, polarity, flexibility, and saturation. The bioavailable radars for the investigated compounds in this work are given (Fig. S11 at ESI†). Accordingly, the pink zone for all examined compounds has four parameters that provide physicochemical space acceptable for oral bioavailability. However, saturation and polarity were found beyond the bioavailability radar's pink zone due to a low saturation (fraction of Csp^3^ < 0.25) and high polarity (TPSA > 130 Å^2^).

### 2.3. Bio-evaluation

#### 2.3.1. Cytotoxicity evaluation

**Fig. 6:**
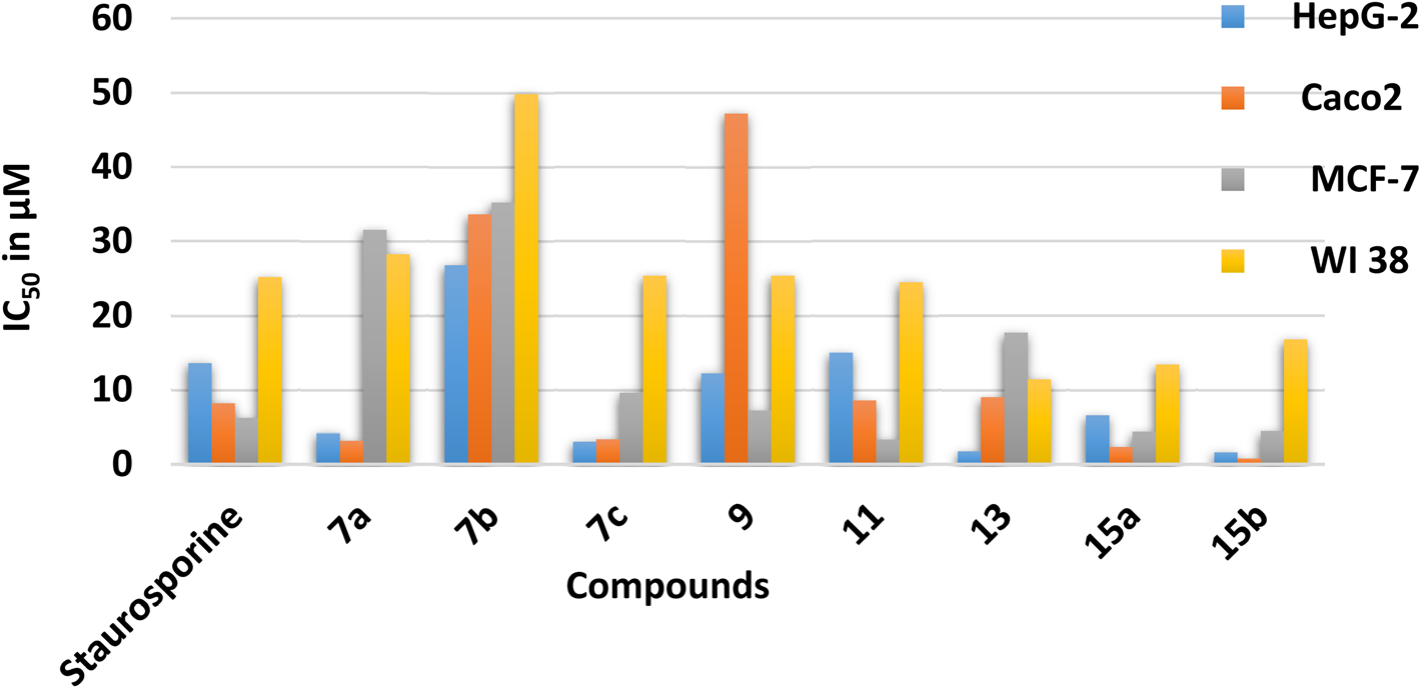
The *in vitro* cytotoxic evaluation of derivatives 7a–c, 9, 11, 13, 15a and 15b presented as IC_50_ in μM.

Screening of the cytotoxic effects of the synthesized compounds compared with staurosporine as a positive control against three human cancer cell lines: hepatocellular carcinoma (HepG-2), colon cancer (Caco-2), breast cancer (MCF-7) and one normal lung fibroblast (WI-38) (*vide*Table 3). The activity was determined using the standard MTT colorimetric assay.^41,42^Fig. 6 summarizes the results of the *in vitro* cytotoxic evaluation of synthesized compounds. As seen from Table 3, compounds 7a, 7c, 9, 13, 15a, and 15b displayed more activity against the HepG-2 cell line than staurosporine (IC_50_ = 13.60 μM). However, compound 11 (IC_50_ = 15.00 μM) showed nearly equipotent activity to staurosporine, and compound 7b showed the highest activity among the synthesized compound (IC_50_ = 26.80 μM). As for activity against Caco2, compounds 7a, 7c, 15a, and 15b (IC_50_ = 5.28, 3.16, 3.37, 2.33, and 0.78 μM, respectively) were more active than staurosporine (IC_50_ = 8.18 μM). As well as, compounds 11 and 13 (IC_50_ = 8.55 and 8.99 μM, respectively) exhibited almost similar activity to staurosporine. Moreover, compounds 7b and 9 (IC_50_ = 33.60 and 47.20 μM) showed moderate activity against the same cell line. Concerning the activity against the MCF-7 cell line, compounds 11 and 15a (IC_50_ = 3.34, 4.34, and 4.43 μM) were more active than staurosporine (IC_50_ = 6.90 μM). On the other hand, compounds 7c, 9, and 13 (IC_50_ = 9.59, 7.21, and 17.70 μM, respectively) showed high growth-inhibitory activity against MCF-7cell line compared with the moderate activity of compounds 7a and 7b (IC_50_ = 31.50 and 35.20 μM, respectively). Compounds 7a and 7b (IC_50_ = 28.20, and 49.80 μM, respectively) displayed less toxicity against the normal fibroblast cell line than staurosporine (IC_50_ = 25.20 μM).

## 3. Experimental section

### 3.1. Synthesis and spectroscopic characterization

#### 3.1.1. Synthesis of 5-acetyl-3-*N*-(4-sulfamoylphenyl)-2-imino-1,3,4-thiadiazoline (4)

A solution of propanehydrazonoyl chloride 1 (1.37 g, 0.005 mol) and ammonium thiocyanate (0.76 g, 0.005 mol) in EtOH (30 mL) was refluxed for 3 h. The reaction mixture was cooled and poured onto ice-cold water (40 mL). The formed precipitate was filtered and recrystallized from EtOH to give compound 4 as orange crystals (1.30 g, 87%); M.P. 181–182 °C. IR *ν*_max_/cm^−1^ = 3352, 3289 (NH_2_), 3277 (NH), 1692 (C=O), 1599 (C=N), 1579 (C=C), 1330, 1298 (SO_2_); ^1^H-NMR (DMSO-d_6_): *δ*_ppm_ = 2.50 (s, 3H, CH_3_), 7.45 (s, 2H, NH_2_), 7.92 (d, *J* = 9.35 Hz, 2H, Ar–H_2_,_6_), 8.18 (d, *J* = 9.35 Hz, 2H, Ar–H_3_,_5_), 9.62 (s, 1H, NH); ^13^C-NMR (DMSO-d_6_): *δ*_ppm_ = 24.88 (CH_3_), 122.00 (2CH, Ar–C_2,6_), 126.54 (2CH, Ar–C_3,5_), 141.28 (Ar–C_4_), 141.48 (Ar–C_1_), 148.04 (thiadiazole-C_5_), 158.30 (thiadiazole-C_2_), 189.83 (C=O); MS *m*/*z* (%): 298 (M^+^, 55.9), 297 (43.7), 241 (0.9), 218 (1.0), 198 (10.2), 182 (0.8), 181 (4.6), 176 (1.3), 172 (3.5), 169 (1.8), 148 (1.0), 134 (2.2), 120 (1.1), 106 (100), 93 (2.3), 78 (10.0), 54 (4.9). Anal. calc. For C_10_H_10_N_4_O_3_S_2_ (298.34): C, 40.26; H, 3.38; N, 18.78%; found: C, 40.24; H, 3.39; N, 18.77%.

#### 3.1.2. Synthesis of (*E*)-4-(5-(1-hydrazineylideneethyl)-2-imino-1,3,4-thiadiazol-3(2*H*)-yl) benzenesulfonamide (5)

A solution of 4 (2.98 g, 0.01 mol) and N_2_H_4_ (1.5 mL, 0.03 mol) in EtOH (40 mL) containing AcOH (3 drops) under reflux for 6 h. The precipitate was filtered and recrystallized from EtOH to get product 5 as a brown powder (2.43 g, 78%); M.P. 207–210 °C. IR *ν*_max/_cm^−1^ = 3416, 3285, 3233, 3119 (2NH_2_), 3065 (NH), 1645 (C=N), 1585 (C=C), 1323, 1305 (SO_2_); ^1^H-NMR (DMSO-d_6_): *δ*_ppm_ = 2.07 (s, 3H, CH_3_), 5.66 (s, 2H, NH_2_), 7.42 (s, 2H, NH_2_), 7.84 (d, *J* = 8.5 Hz, 2H, Ar–H_2,6_), 7.86 (d, *J* = 8.5 Hz, 2H, Ar–H_3,5_), 9.49 (s, 1H, NH); 2.05 (s, 3H, CH_3_), 7.32 (s, 1H, ^2^N-H^b^), 7.33 (s, 1H, ^2^N-H^a^), 7.36 (s, 2H, NH_2_), 8.26 (d, *J* = 8.5 Hz, 2H, Ar–H_2,6_), 8.34 (d, *J* = 8.5 Hz, 2H, Ar–H_3,5_), 9.49 (s, 1H, NH); ^13^C-NMR (DMSO-d_6_): *δ*_ppm_ = 11.55 (CH_3_), 120.24 (2CHAr–C_2,6_), 126.80 (2CHAr–C_3,5_), 131.35 (Ar–C_4_), 139.75 (Ar–C_1_), 145.89 (C=N), 151.28 (thiadiazole-C_5_), 159.23 (thiadiazole-C_2_); MS *m*/*z* (%): 312 (M^+^, 10.3), 270 (1.3), 251 (2.5), 227 (2.2), 205 (1.4), 193 (1.5), 176 (9.6), 158 (2.4), 145 (2.2), 129 (7.1), 111 (8.9), 88 (2.2), 65 (11.7), 57 (100); anal. calc. For C_10_H_12_N_6_O_2_S_2_ (312.37): C, 38.45; H, 3.87; N, 26.90%; found: C, 38.46; H, 3.86; N, 26.91%.

#### 3.1.3. General synthetic procedure for unsymmetrical azines: 1-arylidene (hetaryalidene)-2-(1-(2-imino-3-(4-sulfamoylphenyl)-1,3,4-thiadiazole-5-yl)ethylidene) hydrazines (7a–c, 9, 11)

A mixture of 5 (1 g, 0.003 mol), and 0.003 mol of an aromatic aldehydes 6a–c, 8 or heterocyclic aldehyde 10, was refluxed in EtOH (40 mL) containing AcOH (3 drops) under reflux for 3^_^8 h. The precipitate was filtered and recrystallized from EtOH to afford the desired compounds (7a–c, 9, 11, respectively).

##### 3.1.3.1. 4-[2-Imino-5-(-1-((4-methoxybenzylidene)hydrazineylidene)ethyl)-1,3,4-thiadiazol-3(2H)-yl]benzenesulfonamide (7a)

The compound was obtained by heating a solution of 4-methoxybenzaldehyde 6a (0.43 g, 0.003 mol) for 5 h under reflux. Brown powder (0.70 g, 54%); M.P. 249–252 °C; IR *ν*_max/_cm^−1^ = 3360, 3255 (NH_2_), 3110 (NH), 1604 (C=N), 1550 (C=C), 1332, 1305 (SO_2_); ^1^H-NMR (DMSO-*d*_*6*_): *δ*_ppm_ = 2.49 (s, 3H, CH_3_), 3.84 (s, 3H, OCH_3_), 7.07 (d, *J* = 9.3 Hz, 2CH, Ar–H_3′,5′_), 7.49 (s, 2H, NH_2_), 7.88 (d, *J* = 9.3 Hz, 2CHAr–H_2′,6′_), 7.97 (d, *J* = 2.5 Hz, 2CH, Ar–H_2,6_), 8.03 (d, *J* = 2.5 Hz, 2CHAr–H_3,5_), 8.63 (s, 1H, CH=N), 9.42 (s, 1H, C=NH); ^13^C-NMR (DMSO-d_6_): *δ*_ppm_ = 13.46 (CH_3_), 55.85 (OCH_3_), 114.87 (2CHAr–C_3′,5′_), 121.32 (2CHAr–C_2,6_), 126.88 (2CHAr–C_2′,6′_), 127.34 (Ar–C_1′_), 131.16 (2CHAr–C_3_,_5_), 131.33 (Ar–C_4_), 142.91 (Ar–C_1_), 149.40 (Ar–C_4′_), 161.91 (CH=N), 162.71 (C=N), 162.89 (thiadiazole-C_5_), 168.75 (thiadiazole-C_2_); MS *m*/*z* (%): 430 (M^+^, 4.2), 429 (0.4), 417 (3.9), 404 (11.2), 386 (0.09), 357 (0.1), 333 (0.1), 318 (0.1), 299 (0.1), 279 (0.1), 264 (0.4), 247 (0.2), 225 (0.7), 200 (1.5), 177 (6.4), 160 (2.1), 147 (4.3), 115 (3.0), 77 (100), 63 (50.4), 57 (31.6); anal. calc. For C_18_H_18_N_6_O_3_S_2_ (430.50); C, 50.22; H, 4.21; N, 19.52%; found: C, 50.21; H, 4.22; N, 19.51%.

##### 3.1.3.2. 4-[5-(1-((4-(Dimethylamino)benzylidene)hydrazineylidene)ethyl)-2-imino-1,3,4-thiadiazol-3(2H)-yl]benzenesulfonamide (7b)

The compound was obtained by heating a solution of 4-(dimethylamino)benzaldehyde 6b (0.47 g, 0.003 mol) for 4 h under reflux. Brown powder (0.45 g, 34%); M.P. 235–237 °C; IR *ν*_max/_cm^−1^ = 3310, 3225 (NH_2_), 3116 (NH), 1601 (C=N), 1524 (C=C), 1324, 1230 (SO_2_); ^1^H-NMR (DMSO-d_6_): *δ*_ppm_ = 2.46 (s, 3H, CH_3_), 3.02 (s, 6H, 2CH_3_), 7.39 (s, 2H, NH_2_), 6.77 (d, *J* = 9.3 Hz, 2CH, Ar–H_2′,6′_), 7.73 (d, *J* = 9.3 Hz, 2CH, Ar–H_3′,5′_), 7.90 (d, *J* = 8.5 Hz, 2CH, Ar–H_2,6_), 8.04 (d, *J* = 8.5 Hz, 2CH, Ar–H_3,5_), 8.43 (s, 1H, CH), 9.38 (s, 1H, NH); ^13^C-NMR (DMSO-d_6_): *δ*_ppm_ = 13.33 (CH_3_), 40.30 (2CH_3_), 112.08 (2CHAr–C_3′,5′_), 121.19 (2CHAr–C_2,6_), 122.02 (Ar–C_1′_), 126.87 (2CHAr–C_3,5_), 127.33 (Ar–C_4_), 131.31 (2CHAr–C_2′,6′_), 142.80 (Ar–C_1_), 153.32 (Ar–C_4′_), 158.83 (CH=N), 162.66 (C=N), 163.69 (thiadiazole-C_5_), 168.80 (thiadiazole-C_2_); MS *m*/*z* (%): 443 (M^+^, 12.9), 442 (1.4), 428 (1.1), 414 (3.0), 383 (1.3), 353 (0.9), 342 (1.1), 299 (1.0), 263 (1.0), 251 (1.9), 223 (1.0), 195 (1.0), 181 (1.3), 149 (3.6), 116 (6.2), 90 (17.8), 63 (18.4), 57 (100); anal. calc. For C_19_H_21_N_7_O_2_S_2_ (443.54): C, 51.45; H, 4.77; N, 22.11%; found: C, 51.46; H, 4.78; N, 22.12%.

##### 3.1.3.3. 4-[2-Imino-5-(1-((4-nitrobenzylidene)hydrazineylidene)ethyl)-1,3,4-thiadiazol-3(2H)-yl]benzenesulfonamide (7c)

The compound was obtained by heating a solution of 4-nitrobenzaldehyde 6c (0.48 g, 0.003 mol) for 7 h under reflux. Brown powder (0.84 g, 63%); M.P. 177–179 °C; IR *ν*_max/_cm^−1^ = 3305, 3258 (NH_2_), 3112 (NH), 1623 (C=N), 1516 (C=C), 1307, 1291 (SO_2_); ^1^H-NMR (DMSO-d_6_): *δ*_ppm_ = 2.47 (s, 3H, CH_3_), 7.49 (s, 2H, NH_2_), 7.90 (d, *J* = 1.7 Hz, 2CH, Ar–H_2,6_), 8.04 (d, *J* = 1.7 Hz, 2CHAr–H_3,5_), 8.16 (d, *J* = 3.4 Hz, 2CH, Ar–H_2′,6′_), 8.36 (d, *J* = 3.4 Hz, 2CHAr-H_3′,5′_), 8.67 (s, 1H, N=CH), 9.49 (s, 1H, NH); ^13^C-NMR (DMSO-d_6_): *δ*_ppm_ = 14.31 (CH_3_), 121.53 (2CHAr–C_2,6_), 124.73 (2CHAr–C_3′,5′_), 127.04 (2CHAr–C_2′,6′_), 130.12 (2CHAr–C_3_,_5_), 130.23 (Ar–C_4_), 140.05 (Ar–C_1_), 142.16 (Ar–C_1′_), 149.40 (N=CH), 151.55 (Ar–C_4′_), 158.41 (C=N), 159.60 (thiadiazole-C_5_), 168.70 (thiadiazole-C_2_); MS *m*/*z* (%): 445 (M^+^, 7.9), 444 (8.3), 429 (2.7), 416 (2.8), 393 (0.6), 368 (1.9), 354 (1.9), 335 (0.6), 308 (0.8), 269 (0.7), 237 (0.8), 223 (0.6), 205 (1.5), 190 (19.8), 174 (2.0), 141 (3.0), 119 (20.3), 95 (16.1), 73 (16.7), 57 (100); anal. calc. For C_17_H_15_N_7_O_4_S_2_ (445.47): C, 45.84; H, 3.39; N, 22.01%; found: C, 45.83; H, 3.40; N, 22.02%.

##### 3.1.3.4. 4-[2-Imino-5-(1-((2,4,6-trimethoxybenzylidene)hydrazineylidene)ethyl)-1,3,4-thiadiazol-3(2H)-yl]benzenesulfonamide (9)

The compound was obtained by heating a solution of 2,4,6-trimethoxybenzaldehyde 8 (0.62 g, 0.003 mol) for 5 h under reflux. Brown powder; (0.79 g, 54%); M.P. 189–192 °C; IR *ν*_max/_cm^−1^ = 3305, 3247 (NH_2_), 3115 (NH), 1620 (C=N), 1592 (C=C), 1325, 1294 (SO_2_); ^1^H-NMR (DMSO-d_6_): *δ*_ppm_ = 2.50 (s, 3H, CH_3_), 3.82 (s, 3H, OCH_3_), 3.86 (s, 6H, 2OCH_3_), 6.33 (s, 2H, Ar–H), 7.42 (s, 2H, NH_2_), 7.97 (d, *J* = 8.5 Hz, 2H, Ar–H_2,6_), 8.01 (d, *J* = 8.5 Hz, 2H, Ar–H_3,5_), 8.68 (s, 1H, =CH), 10.3 (s, 1H, NH); ^13^C-NMR (DMSO-d_6_): *δ*_ppm_ = 13.55 (CH_3_), 56.50 (CH_3_), 56.64 (CH_3_), 56.76 (CH_3_), 91.64 (2CHAr–C_3′,5′_), 103.76 (Ar–C_1′_), 122.13 (2CHAr–C_2,6_), 122.29 (2CHAr–C_3,5_), 127.34 (Ar–C_4_), 127.38 (Ar–C_1_), 142.85 (thiadiazole-C_5_), 143.07 (C=N), 149.4 (CH=N), 159.2 (Ar–C_4′_), 161.98 (Ar–C–O), 168.89 (thiadiazole-C_2_); MS *m*/*z* (%): 490 (M^+^, 6.9), 489 (8.3), 472 (1.9), 461 (1.9), 443 (2.3), 408 (3.5), 378 (2.7), 354 (2.2), 322 (2.6), 280 (3.2), 266 (3.3), 229 (3.0), 217 (4.6), 196 (3.6), 179 (100), 142 (4.4), 121 (32.4), 76 (26.6), 57 (41.5); anal. calc. For C_20_H_22_N_6_O_5_S_2_ (490.55): C, 48.98; H, 4.53; N, 17.12%; found: C, 48.99; H, 4.52; N, 17.13%.

##### 3.1.3.5. 4-[5-(1-((Furan-2-ylmethylene)hydrazineylidene)ethyl)-2-oxo-1,3,4-thiadiazol-3(2H)-yl]benzene sulfonamide (11)

The compound was obtained by heating a solution of furan-2-carbaldehyde 10 (0.30 g, 0.003 mol) for 8 h under reflux. Brown powder; (0.50 g, 43%); M.P. 222–223 °C; IR *ν*_max/_cm^−1^ = 3338, 3245 (NH_2_), 1685 (C=O), 1625 (C=N), 1593 (C=C), 1325, 1291 (SO_2_); ^1^H-NMR (DMSO-d_6_): *δ*_ppm_ = 2.44 (s, 3H, CH_3_), 6.72 (d, *J* = 3.4 Hz, 1H, furan-H_3_), 6.92 (t, *J* = 3.4 Hz, 1H, furan-H_4_), 7.21 (d, *J* = 3.4 Hz, 1H, furan-H_2_), 7.47 (s, 2H, NH_2_), 7.98 (d, *J* = 5.1 Hz, 2CH, Ar–H_2,6_), 8.01 (d, *J* = 8.5 Hz, 2CH, Ar–H_3,5_), 8.48 (s, 1H, N=CH); ^13^C-NMR (DMSO-d_6_): *δ*_ppm_ = 13.68 (CH_3_), 113.48 (furan-C_3_), 119.59 (2CHAr–C_2,6_), 121.68 (furan-C_4_), 126.91 (2CHAr–C_3,5_), 139.67 (Ar–C_4_), 146.64 (Ar–C_1_), 148.87 (furan-C_2_), 150.35 (thiadiazole-C_5_), 151.53 (furan-C_5_), 152.54 (C=N), 159.01 (C=NH), 168.29 (C=O); MS *m*/*z* (%): 391 (M^+^, 7.5), 390 (0.6), 378 (0.1), 365 (3.0), 341 (0.1), 311 (0.3), 295 (0.1), 280 (0.1), 262 (0.1), 239 (0.7), 221 (0.1), 205 (0.1), 198 (0.2), 188 (0.1), 176 (0.1), 154 (0.2), 128 (0.4), 116 (1.1), 94 (100), 78 (4.7), 65 (6.0); anal. calc. For C_15_H_13_N_5_O_4_S_2_ (391.42): C, 46.03; H, 3.35; N, 17.89%; found: C, 46.02; H, 3.36; N, 17.90%.

#### 3.1.4. General synthetic procedure for unsymmetrical azines: 1-arylidene (hetaryalidene)-2-(1-(2-imino-3-(4-sulfa-moyl-phenyl)-1,3,4-thiadiazole-5-yl)ethylidene) hydrazines (13, 15a–b)

A solution of 5 (1 g, 0.003 mol) and 0.001 mol of cyclic heterocyclic ketones (12 or 14a–b) was refluxed in absolute EtOH (40 mL) containing AcOH (3 drops) for 3–5 h. The precipitate was filtered and recrystallized from EtOH to yield the desired compounds (13 or 15a–b, respectively).

##### 3.1.4.1. 4-[2-Imino-5-(1-((2-oxoindolin-3-ylidene)hydrazineylidene)ethyl)-1,3,4-thiadiazol-3(2H)-yl]benzenesulfonamide (13)

The compound was obtained by heating a solution of indoline-2,3-dione 12 (0.47 g, 0.003 mol) for 4 h under reflux. Green powder; (0.96 g, 73%); M.P. > 300 °C; IR *ν*_max/_cm^−1^ = 3303, 3257 (NH_2_), 3115, 3019 (2NH), 1717 (C=O), 1607 (C=N), 1587 (C=C), 1322, 1292 (SO_2_); ^1^H-NMR (DMSO-d_6_): *δ*_ppm_ = 2.32 (s, 3H, CH_3_), 6.90–7.39 (m, 4H, Ar–H), 7.41 (s, 2H, NH_2_), 7.89 (d, *J* = 8.5 Hz, 2H, Ar–H_2,6_), 8.23 (d, *J* = 8.5 Hz, 2H, Ar–H_3,5_), 9.54 (s, 1H, NH), 10.92 (s, 1H, indole-NH); ^13^C-NMR (DMSO-d_6_): *δ*_ppm_ = 13.49 (CH_3_), 111.12 (2CHAr–C_2,6_), 116.13 (indole-C_4′_), 121.22 (indole-C_7_), 122.47 (indole-C_5_), 126.48 (indole-C_4_), 126.94 (2CHAr–C_3,5_), 128.19 (Ar–C_4_), 134.34 (indole-C_6_), 140.80 (indole-C_3′_), 141.70 (indole-C_5′_), 145.40 (Ar–C_1_), 147.30 (thaiadiazole-C_5_), 154.71 (C=N), 158.09 (thiadiazole-C_2_), 163.91 (C=O); MS *m*/*z* (%): 441 (M^+^, 11.5), 440 (2.3), 417 (0.7), 381 (0.7), 367 (1.3), 335 (0.7), 300 (1.7), 292 (0.7), 266 (0.9), 232 (0.6), 206 (2.3), 171 (2.5), 144 (9.3), 104 (100), 65 (41.8), 50 (34.8); anal. calc. For C_18_H_15_N_7_O_3_S_2_ (441.48): C, 48.97; H, 3.42; N, 22.21%; found: C, 48.96; H, 3.41; N, 22.22%.

##### 3.1.4.2. 4-[5-(1-((1-((Dimethylamino)methyl)-2-oxoindolin-3-ylidene)hydrazineylidene)ethyl)-2-imino-1,3,4-thiadiazol-3(2H)-yl]benzenesulfonamide (15a)

The compound was obtained by heating a solution of 1-((dimethylamino)methyl)indoline-2,3-dione 14a (0.65 g, 0.003 mol) for 3 h under reflux. Brown powder; (1.09 g, 54%); 73%; M.P. > 300 °C; IR *ν*_max/_cm^−1^ = 3303, 3269 (NH_2_), 3115 (NH), 1721 (C=O), 1609 (C=N), 1590 (C=C), 1320, 1304 (SO_2_); ^1^H-NMR (DMSO-d_6_): *δ*_ppm_ = 2.42 (s, 3H, CH_3_), 2.50 (s, 6H, 2CH_3_), 4.36 (s, 2H, CH_2_–N), 6.92 (d, *J* = 7.6 Hz, 1CH, indole-H_7_), 7.06 (t, *J* =7.6 Hz, 1CH, indole-H_5_), 7.44(t, *J* = 7.6 Hz, 1CH, indole-H_6_), 7.49 (s, 2H, NH_2_), 7.61 (d, *J* = 7.6 Hz, 1CH, indole-H_4_), 7.90 (d, *J* = 8.5 Hz, 2CH, Ar–H_2,6_), 8.01 (d, *J* = 8.5 Hz, 2CH, Ar–H_3,5_), 10.94 (s, 1H, NH); ^13^C-NMR (DMSO-d_6_): *δ*_ppm_ = 14.18 (CH_3_), 40.39 (CH_3_)_2_, 65.03 (N–CH_2_–N), 111.55 (2CHAr–C_2,6_), 116.49 (indole-C_7_), 121.17 (indole-C_5_), 123.06 (indole-C_4_), 127.85 (2CHAr–C_3,5_), 128.80 (Ar–C_4_), 142.00 (Ar–C_1_), 147.61 (thiadiazole-C_5_), 153.27 (C=N), 154.63 (thiadiazole-C_2_), 164.35 (C=O); MS *m*/*z* (%): 498 (M^+^, 2.6), 497 (3.1), 462 (2.9), 402 (2.6), 336 (3.0), 321 (2.5), 304 (2.6), 270 (3.6), 256 (3.1), 220 (3.8), 202 (2.5), 188 (6.1), 164 (4.0), 128 (7.0), 111 (16.0), 90 (40.7), 67 (31.5), 55 (100); anal. calc. For C_21_H_22_N_8_O_3_S_2_ (498.58): C, 50.59; H, 4.45; N, 22.48%; found: C, 50.58; H, 4.44; N, 22.47%.

##### 3.1.4.3. 4-[5-(1-((1-((Diethylamino)methyl)-2-oxoindolin-3-ylidene)hydrazineylidene)ethyl)-2-imino-1,3,4-thiadiazol-3(2H)-yl)benzenesulfonamide (15b)

The compound was obtained by heating a solution of 1-((diethylamino)methyl)indoline-2,3-dione 14b (0.74 g, 0.003 mol) for 5 h under reflux. Brown powder; (1.22 g, 77%); M.P. > 300 °C; IR *ν*_max/_cm^−1^ = 3305, 3264 (NH_2_), 3115 (NH), 1728 (C=O), 1609 (C=N), 1589 (C=C), 1380, 1304 (SO_2_); ^1^H-NMR (DMSO-d_6_): *δ*_ppm_ = 2.03 (t, *J* = 7.6 Hz, 3H, CH_3_), 2.34 (s, 3H, CH_3_), 2.92 (q, *J* = 7.6 Hz, 2H, CH_2_), 4.36 (s, 2H, CH_2_), 6.92 (d, *J* = 7.6 Hz, 1CH, indole-H_7_), 7.06 (t, *J* = 7.6 Hz, 1CH, indole-H_5_), 7.41 (s, 2H, NH_2_), 7.45 (t, *J* =7.6 Hz, 1CH, indole-H_6_), 7.90 (d, *J* = 8.5 Hz, 2CH, Ar–H_2,6_), 8.01 (d, *J* = 8.5 Hz, 2CH, Ar–H_3,5_), 9.56 (s, 1H, NH); ^13^C-NMR (DMSO-d_6_): *δ*_ppm_ = 11.52 (CH_3_), 14.18 (CH_3_), 41.61 (CH_2_N), 65.03 (N–CH_2_–N), 111.58 (2CHAr–C_2,6_), 116.49 (indole-C_4_), 121.17 (indole-C_7_), 123.05 (indole-C_5_), 126.95 (indole-C_4_), 127.34 (2CHAr–C_3,5_), 127.85 (Ar–C_4_), 128.74 (1CHAr–C_6_), 134.83 (C=N–N), 142.00 (Ar–C_1_), 145.85 (1H-indole-C_5′_), 147.61 (thiadiazole-C_5_), 148.07 (C=N), 154.60 (thiadiazole-C_2_), 164.29 (C=O); MS *m*/*z* (%): 526 (M^+^, 1.4), 451 (1.9), 409 (1.7), 383 (2.5), 369 (4.7), 325 (2.0), 299 (3.5), 265 (2.9), 241 (1.4), 208 (1.8), 175 (1.9), 166 (2.2), 145 (3.0), 123 (12.7), 110 (10.3), 91 (8.5), 69 (58.8), 57 (100); anal. calc. For C_23_H_26_N_8_O_3_S_2_ (526.63): C, 52.46; H, 4.98; N, 21.28%; found: C, 52.45; H, 4.97; N, 21.29%.

#### 3.1.5. Alternative method for synthesis of unsymmetrical azines: 1-arylidene (hetaryali dene)-2-(1-(2-imino-3-(4-sulfamoylphenyl)-1,3,4-thiadiazole-5-yl)ethylidene)hydrazines (15a–b)

A solution of 13 (1 g, 0.002 mol), 0.002 mol of formalin, and dimethylamine or formalin and diethylamine in EtOH (30 mL) containing concentrated HCl (three drops) under reflux for 12 h. The precipitate was filtered and recrystallized from EtOH to afford the compounds 15a–b, respectively.

### 3.2. Computational details

Gaussian 09 package software^43^ was used to carry out all quantum chemical calculations using the method of DFT-B3LYP^12,13^ combined with a standard 6-31G(d) basis set. The suggested geometries for the synthesized compounds were initially optimized using Pulay's gradient approach.^44^ Frequency calculations were performed to verify that the optimized geometries are actual minimums with real wavenumbers. The geometry of the *E* and *Z* forms of 5 were reoptimized in DMSO solution using the IEF-PCM solvation model,^45^ followed by ^1^H and ^13^C NMR chemical shift calculations applying the GIAO approach.^14,15^ The multi-standard approach^24,25^ was applied to get the theoretical chemical shifts for *E* and *Z* isomers using the isotropic magnetic shielding values (*σ*_i_, ppm) for H and C atoms acquired from the Gaussian output file. In this approach, methanol and benzene were used as references to predict sp^3^ and sp^2^ hybridized Cs/C–Hs, respectively, whereas comparable skeletons were used in the case of N–H protons. Thus, benzenesulfonamide,^46^ acetophenonehydrazone,^47^ and 5-iso-propyl-3-methyl-2-imino-1,3,4-thiadiazole^48^ were used as references to calculate the N–H chemical shifts in the benzenesulfonamide, hydrazone and iminothiadiazoles moieties, respectively. Furthermore, the HOMO/LUMO (FMOs) and energy gaps were predicted for the optimized geometries of the synthesized compounds 7a–c, 9, 11, 13, 15a and 15b. Accordingly, molecular reactivity descriptors such as ionization potential, electron affinity, hardness, chemical potential, and electrophilicity index were calculated using the values of energies for FMOs.^28,29^ The synthesized compounds' pharmacokinetics, drug-like characteristics, and toxicities were evaluated using the SwissADME^20^ and pkCSM^21^ online servers.

### 3.3. Biological evaluation

#### 3.3.1. Cytotoxicity evaluation

HepG2, MCF-7, WI 38, Caco-2 cancer cell lines were cultured in complete media of RPMI and DMEM, respectively, at 5% carbon dioxide and 37 °C following standard tissue culture work. The cells were grown in “10% fetal bovine serum (FBS) and 1% penicillin–streptomycin” in 96-multiwell plate. All the synthesized compounds were screened for their cytotoxicity using 20 μL of MTT solution (Promega, USA) for 48 h using untreated and treated cells with concentrations of (0.01, 0.1, 1, 10, and 100 μM) for 48 h.^41,42^ The plate was cultured for 3 hours. The percentage of cell viability was calculated following this equation: (100 − (*A*_sample_)/(*A*_control_)) × 100. An ELISA microplate reader was used to measure the absorbance at 690 nm to calculate the viability *versus* concentration, and the IC_50_ value using GraphPad prism software.

## 4. Conclusions

Herein, the combi-molecule strategy was used to synthesize a bundle of differently hybrid 1,3,4-thiadiazole sulfonamide derivatives and unsymmetrical azines 7a–c, 9, 11, 13, 15a and 15b started with 5-acetyl-3-*N*-(4-sulfamoylphenyl)-2-imino-1,3,4-thiadiazoline 4. In the solution phase, compound 5 exists as a mixture of *E* and *Z* configurations, according to the results of computed/observed ^1^H and ^13^C NMR chemical shifts. The DFT-B3LYP calculations favor the final synthesized products in the *EE* configuration with reference to the azomethine C=N bonds. For the newly synthesized compounds, quantum chemical descriptors and drug-likeness properties were predicted and correlated to their *in vitro* bioactivities. Experimentally, the synthesized compounds were investigated for their anticancer effects *in vitro* against cancer cell lines: HepG-2, Caco-2, MCF-7, and WI-38, where they exhibited promising results.

## Conflicts of interest

The authors declare that there are no conflicts of interest.

## Supplementary Material

RA-013-D3RA00123G-s001
