# Supplementary material for: Novel asymmetrical azines appending 1,3,4-thiadiazole sulfonamide: synthesis, molecular structure analyses, in silico ADME, and cytotoxic effect
Source: RSC Adv. 2023 Apr 3;13(15):10353–66. doi: 10.1039/d3ra00123g (PMC10068595; doi:10.1039/d3ra00123g)
Supplement: RA-013-D3RA00123G-s001 [file RA-013-D3RA00123G-s001.pdf]

Electronic Supplementary Material (ESI) for RSC Advances.
This journal is © The Royal Society of Chemistry 2023

Supplementary Data

Novel asymmetrical azines appending 1,3,4-thiadiazole sulfonamide: Synthesis, molecular structure analyses, *in silico* ADME, and cytotoxic effect

Samir Bondock*[a,b], Tallah Albarqi[a], Ibrahim A. Shaaban[a,c], Moaz M. Abdou[d]

[a]Chemistry Department, Faculty of Science, King Khalid University, 9004 Abha, Saudi Arabia

[b]Chemistry Department, Faculty of Science, Mansoura University, 35516 Mansoura, Egypt

[c]Department of Chemistry, Faculty of Science (Men's Campus), Al-Azhar University, Nasr City 11884, Cairo, Egypt

[d]Egyptian Petroleum Research Institute, Nasr City, 11727, Cairo, Egypt

Correspondence

Samir Bondock, Chemistry Department, Faculty of Science, Mansoura University, 35516 Mansoura, Egypt.

Email: bondock@mans.edu.eg

1. Materials and instrumentation

1) All melting points were determined on Stuart SMP11 apparatus and were uncorrected.
2) The IR spectra were recorded in KBr discs, on a Jasco FT/IR-460 plus spectrophotometer at College of Science, King Khalid University.
3) The ^{1}H- and ^{13}C-NMR spectra were recorded in DMSO-d_6 at 850 MHZ on a BrukerAvanceAV-850 NMRUltrshield^{TM} spectrometer at King Abdulaziz University, Jeddah, Saudi Arabia.
4) Mass spectra were measured using the Shimadzu GC/MS-QP 1000 EX mass spectrometer at 70 eV, at the Micro Analytical Center, Cairo University, Giza, Egypt.
5) Elemental analyses were carried out at the Micro Analytical Center, Cairo University, Giza, Egypt.
6) Screening of the synthesized compounds in vitro against three human cancer cell lines: hepatocellular carcinoma (HepG-2), colon cancer (Caco-2), breast cancer (MCF-7) and one normal lung fibroblast (WI-38). The cell lines were obtained from American Type Culture Collection (ATCC) by the Holding company for biological products and vaccines (VACSERA), Giza, Egypt.

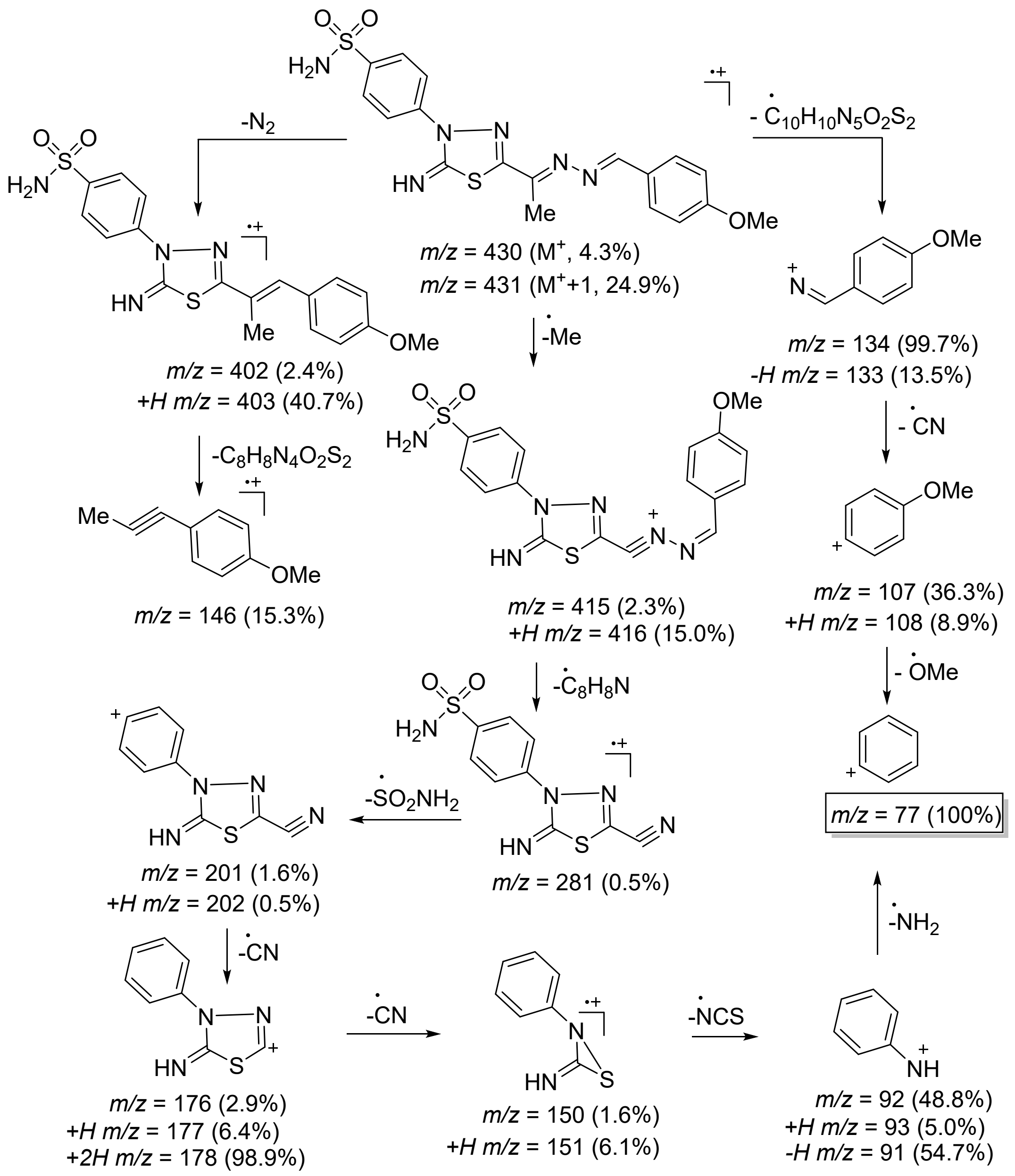

Scheme 1. Mass fragmentation pattern of compound **7a**.

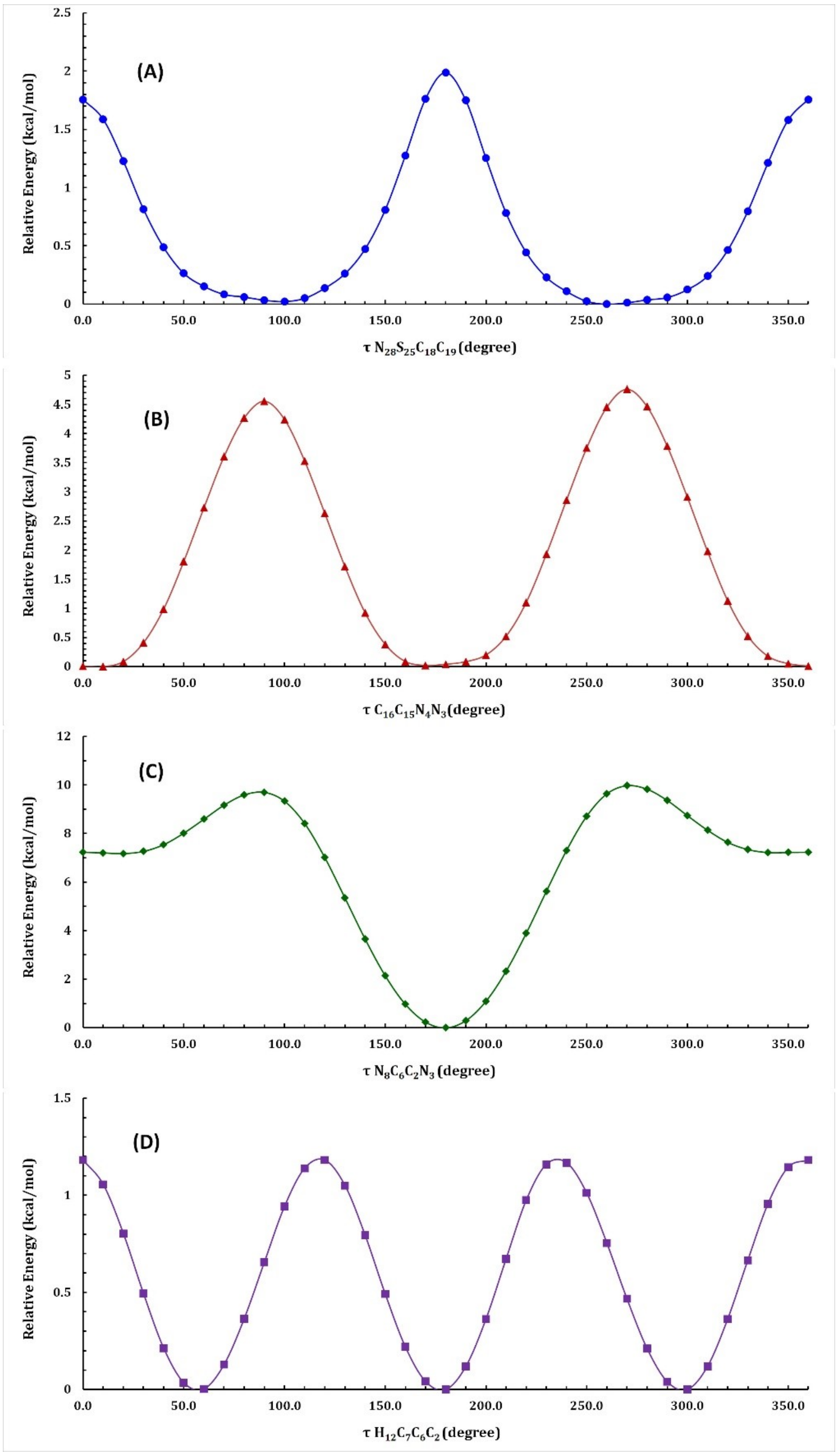

Figure S1. The computed PES for internal rotations in the E isomers of **5**; **(A)** sulfonamide moiety, **(B)** phenyl sulfonamide moiety, **(C)** $C(CH_3)NNH_2$ moiety and **(D)** methyl moiety.

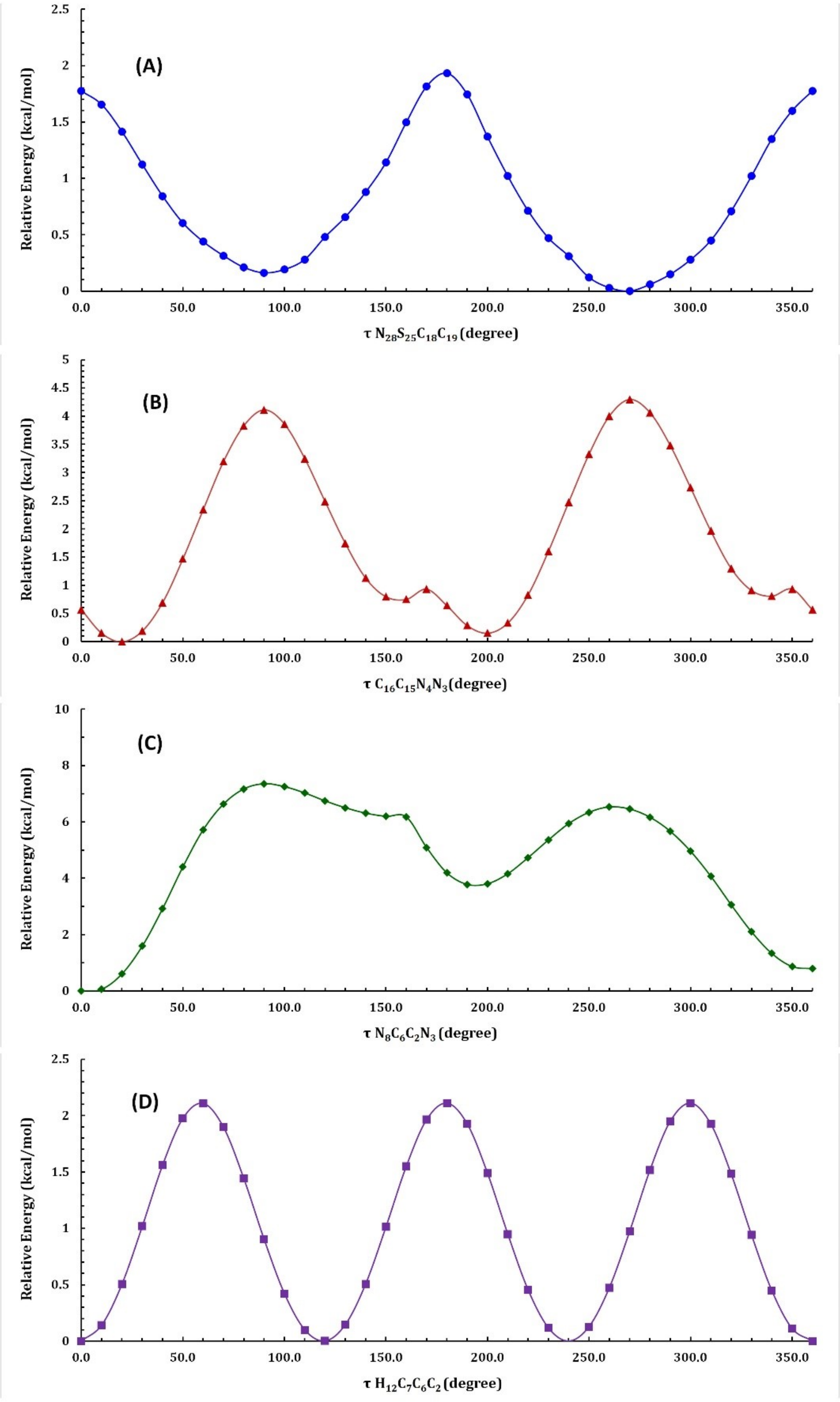

Figure S2. The computed PES for internal rotations in the Z isomers of **5**; **(A)** sulfonamide moiety, **(B)** phenyl sulfonamide moiety, **(C)** $C(CH_3)NNH_2$ moiety and **(D)** methyl moiety.

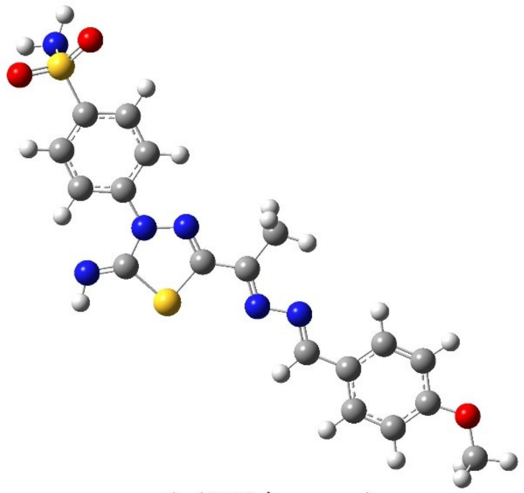

1 (***EE*** isomer)
HF= –2047.174609

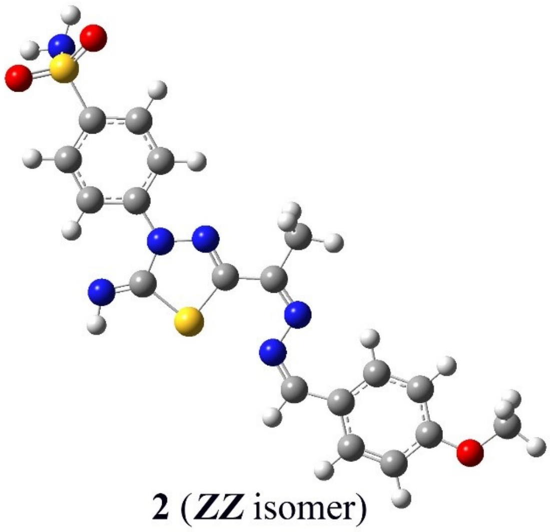

2 (***ZZ*** isomer)
HF= –2047.166237

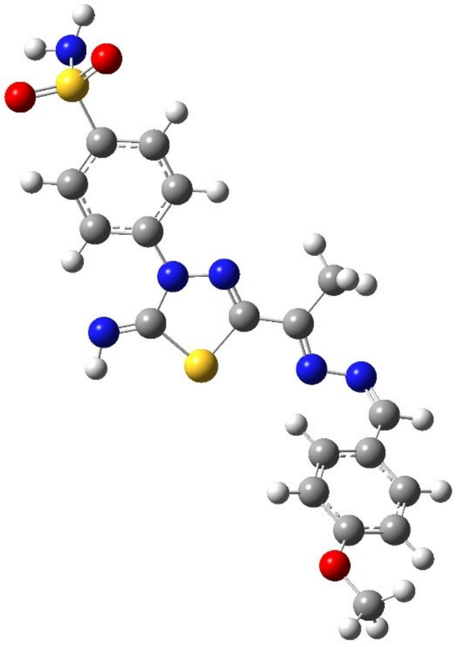

3 (***EZ*** isomer)
HF= –2047.168535

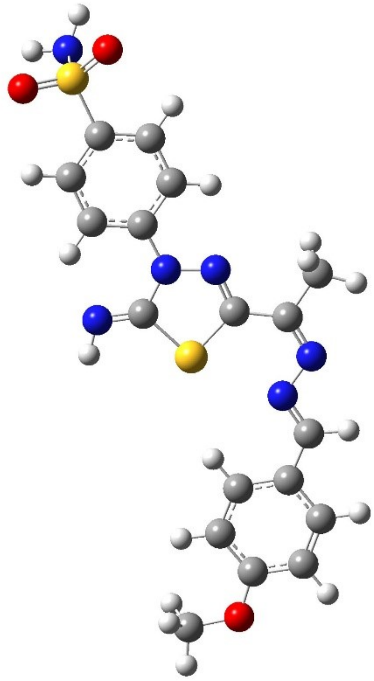

4 (***ZE*** isomer)
HF= –2047.171529

Figure S3. Optimized geometries for the proposed configurations of compound **7a** obtained from B3LYP/6-31G(d) calculations.

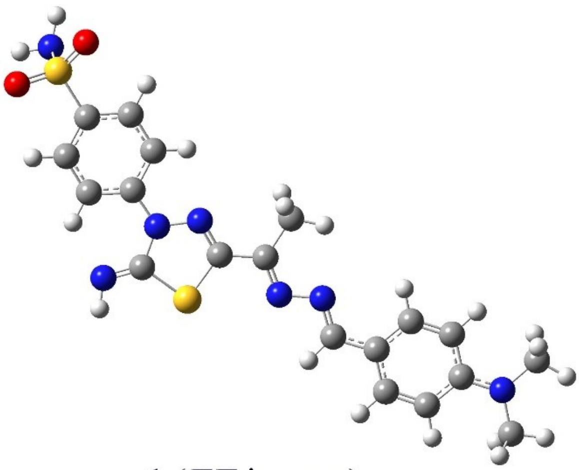

1 (***EE*** isomer)
HF= –2066.621509

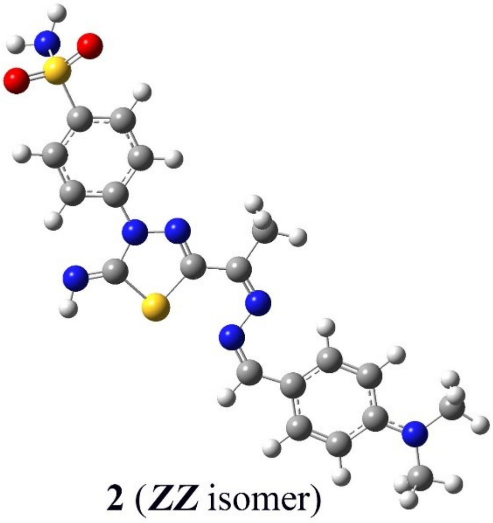

2 (***ZZ*** isomer)
HF= –2066.613383

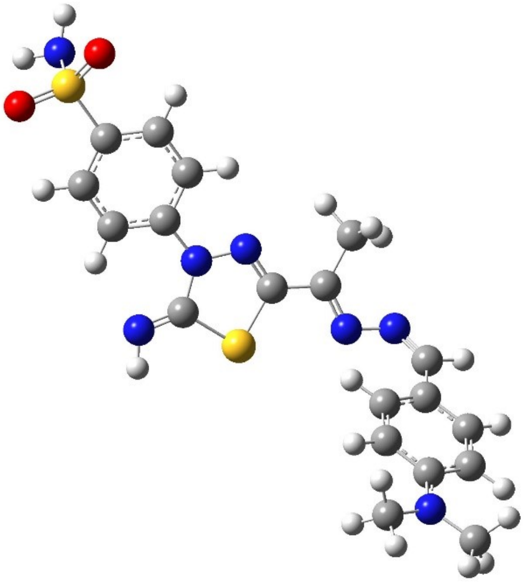

3 (***EZ*** isomer)
HF= –2066.615461

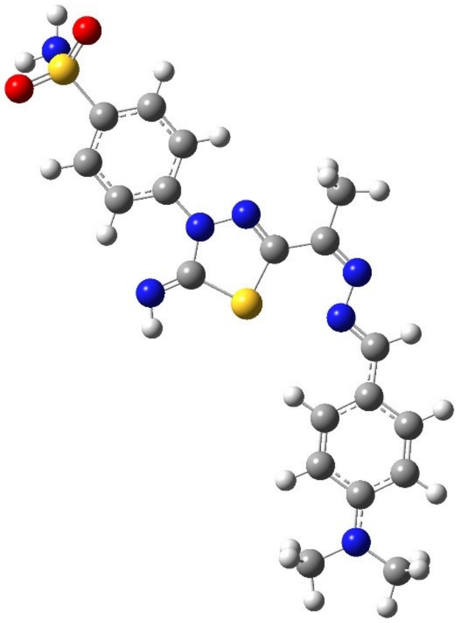

4 (***ZE*** isomer)
HF= –2066.618754

Figure S4. Optimized geometries for the proposed configurations of compound **7b** obtained from B3LYP/6-31G(d) calculations.

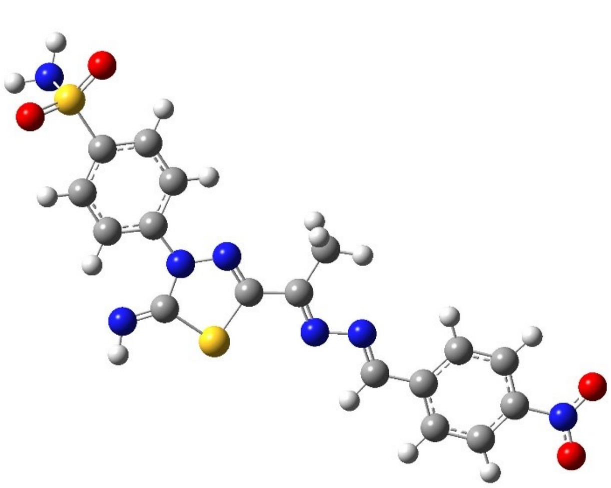

1 (***EE*** isomer)
HF= –2137.148645

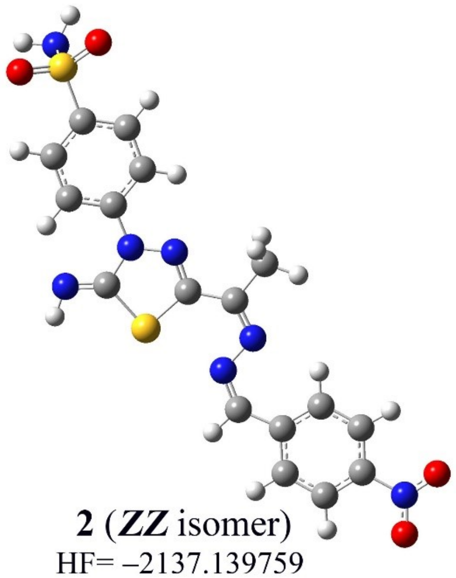

2 (***ZZ*** isomer)
HF= –2137.139759

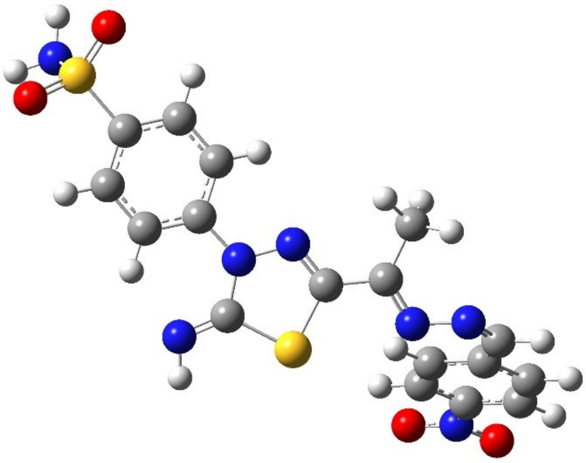

3 (***EZ*** isomer)
HF= –2137.142714

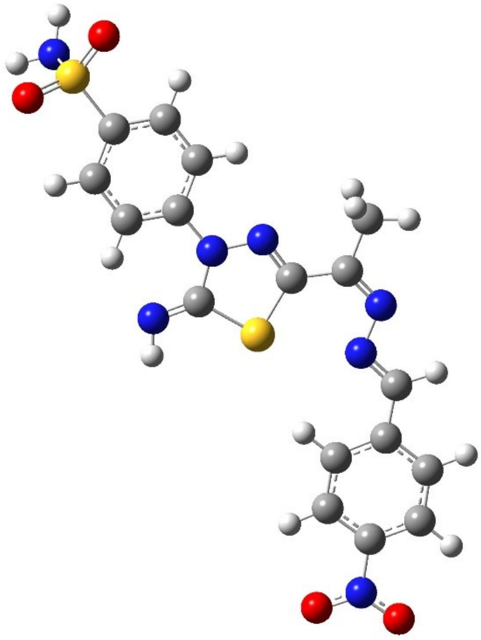

4 (***ZE*** isomer)
HF= –2137.145381

Figure S5. Optimized geometries for the proposed configurations of compound **7c** obtained from B3LYP/6-31G(d) calculations.

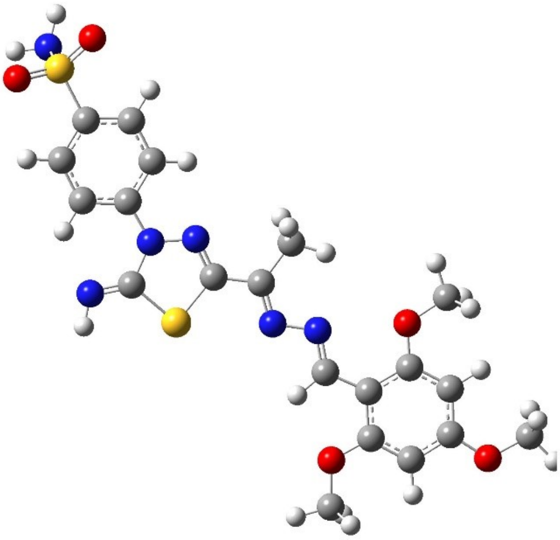

1 (***EE*** isomer)
HF= −2276.213429

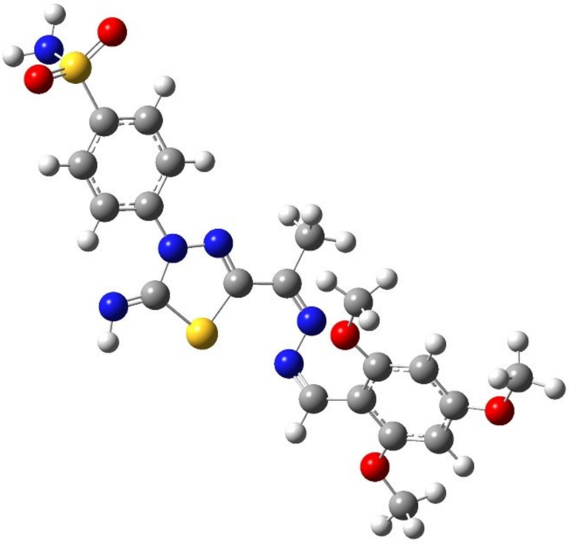

2 (***ZZ*** isomer)
HF= −2276.204309

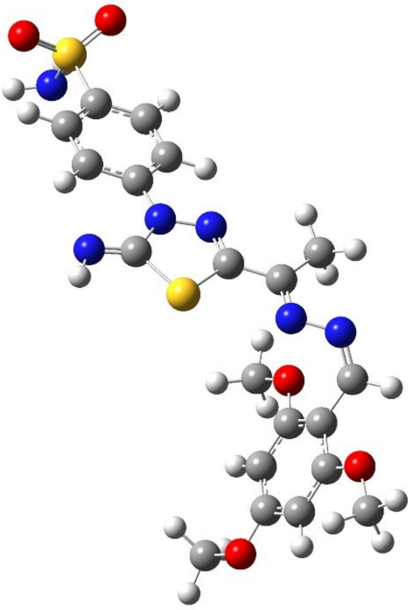

3 (***EZ*** isomer)
HF= −2276.207865

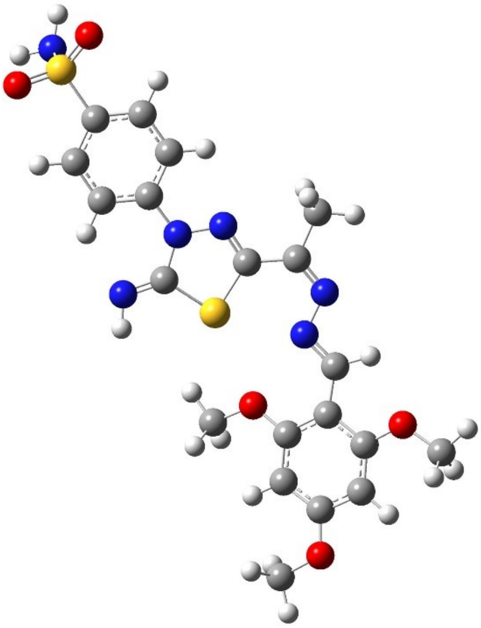

4 (***ZE*** isomer)
HF= −2276.210618

Figure S6. Optimized geometries for the proposed configurations of compound **9** obtained from B3LYP/6-31G(d) calculations.

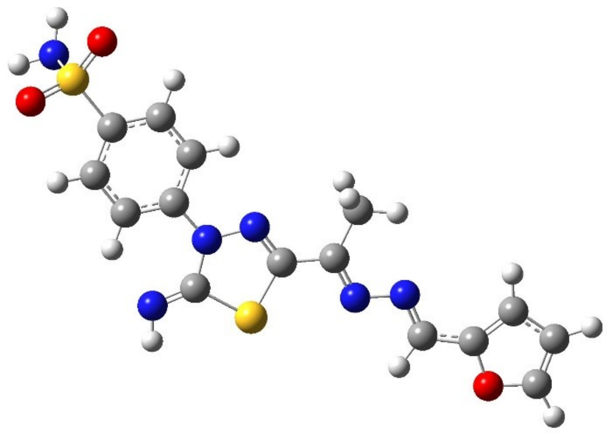

1 (***EE*** isomer)
HF= −1930.42554

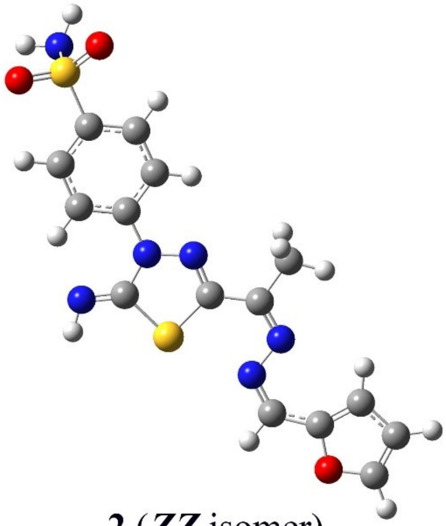

2 (***ZZ*** isomer)
HF= −1930.421288

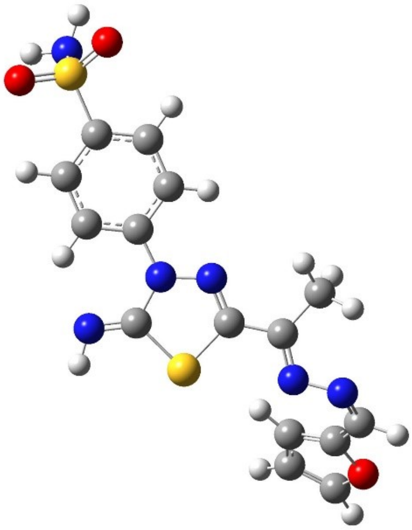

3 (***EZ*** isomer)
HF= −1930.423287

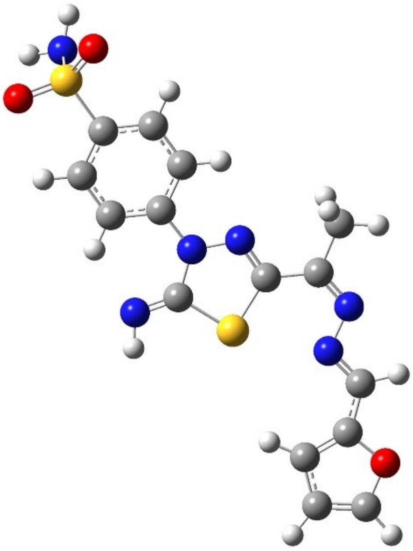

4 (***ZE*** isomer)
HF= −1930.423002

Figure S7. Optimized geometries for the proposed configurations of compound **11** obtained from B3LYP/6-31G(d) calculations.

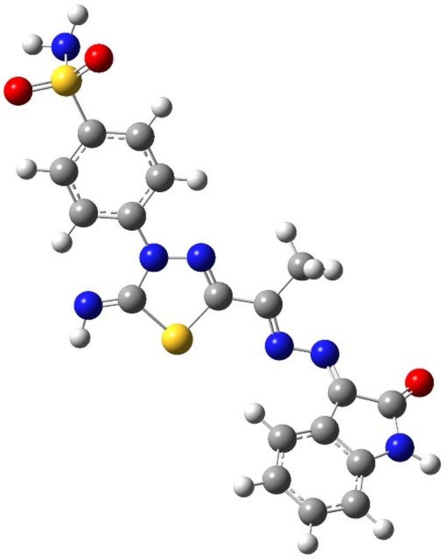

1 (***EE*** isomer)
HF= –2100.141412

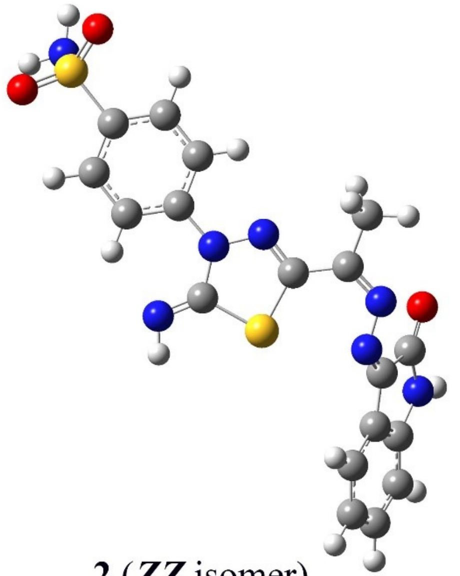

2 (***ZZ*** isomer)
HF= –2100.136203

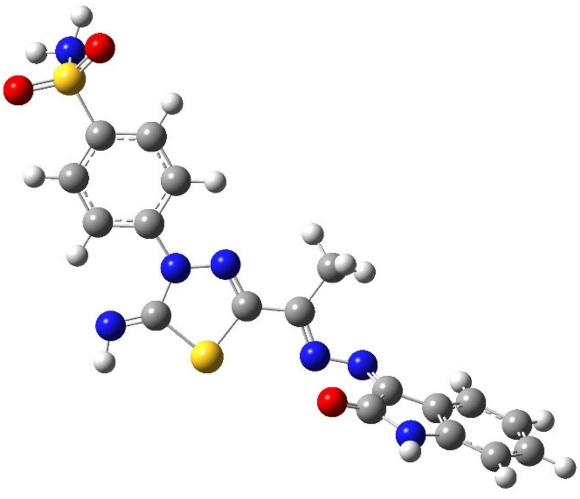

3 (***EZ*** isomer)
HF= –2100.140941

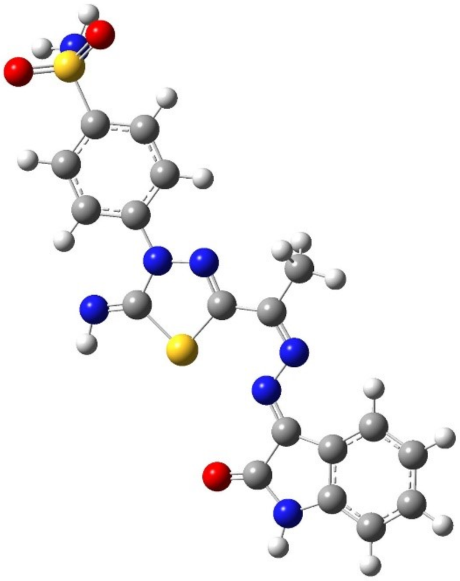

4 (***ZE*** isomer)
HF= –2100.140155

Figure S8. Optimized geometries for the proposed configurations of compound **13** obtained from B3LYP/6-31G(d) calculations.

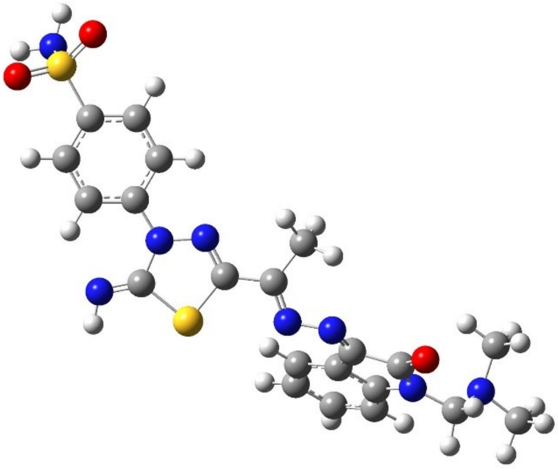

1 (***EE*** isomer)
HF= −2273.419471

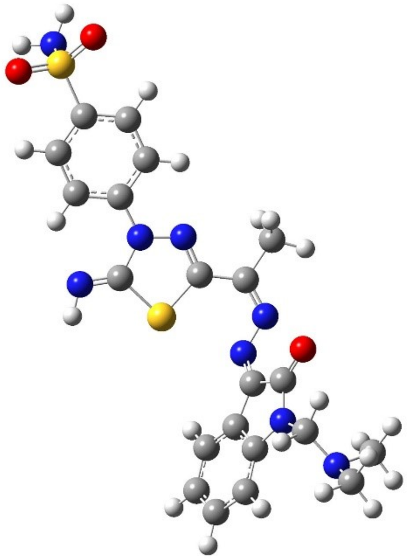

2 (***ZZ*** isomer)
HF= −2273.414291

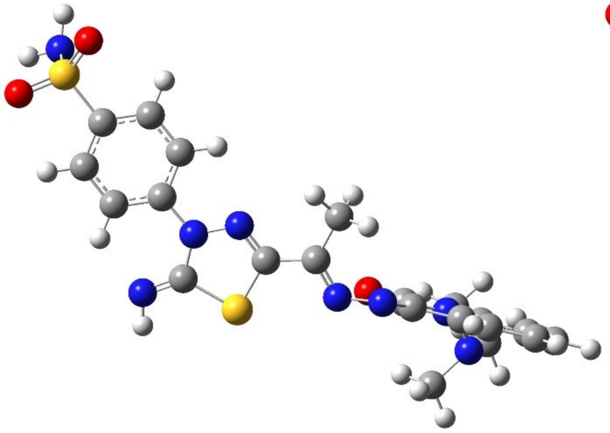

3 (***EZ*** isomer)
HF= −2273.419083

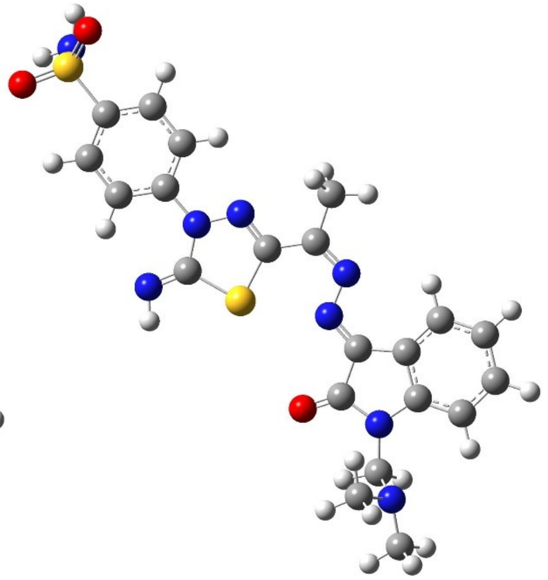

4 (***ZE*** isomer)
HF= −2273.41831

Figure S9. Optimized geometries for the proposed configurations of compound **15a** obtained from B3LYP/6-31G(d) calculations.

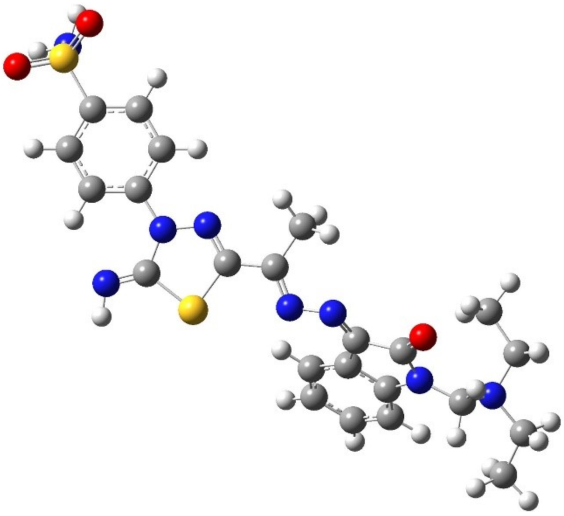

1 (***EE*** isomer)
HF= −2352.04409

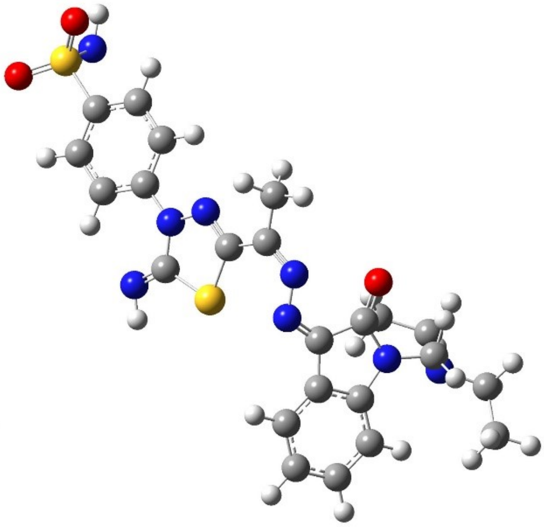

2 (***ZZ*** isomer)
HF= −2352.038863

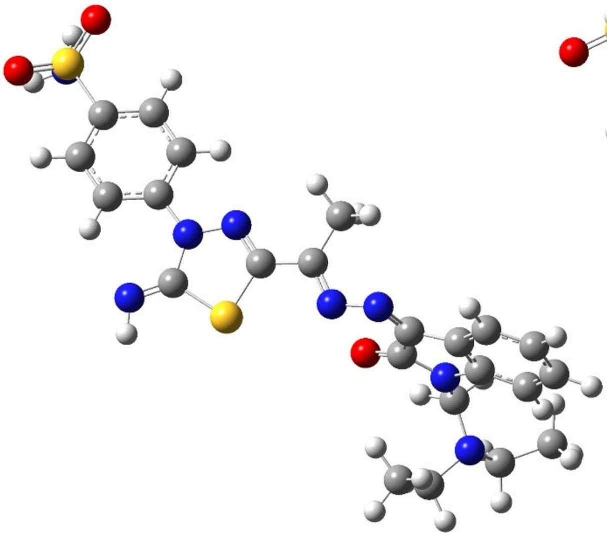

3 (***EZ*** isomer)
HF= −2352.043659

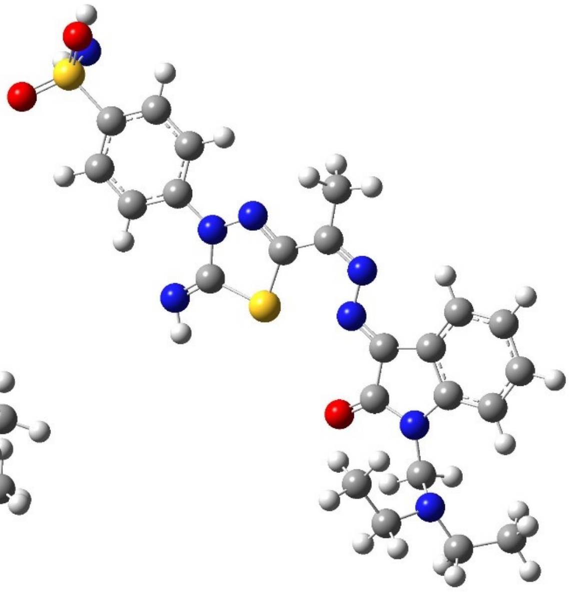

4 (***ZE*** isomer)
HF= −2352.042893

Figure S10. Optimized geometries for the proposed configurations of compound **15b** obtained from B3LYP/6-31G(d) calculations.

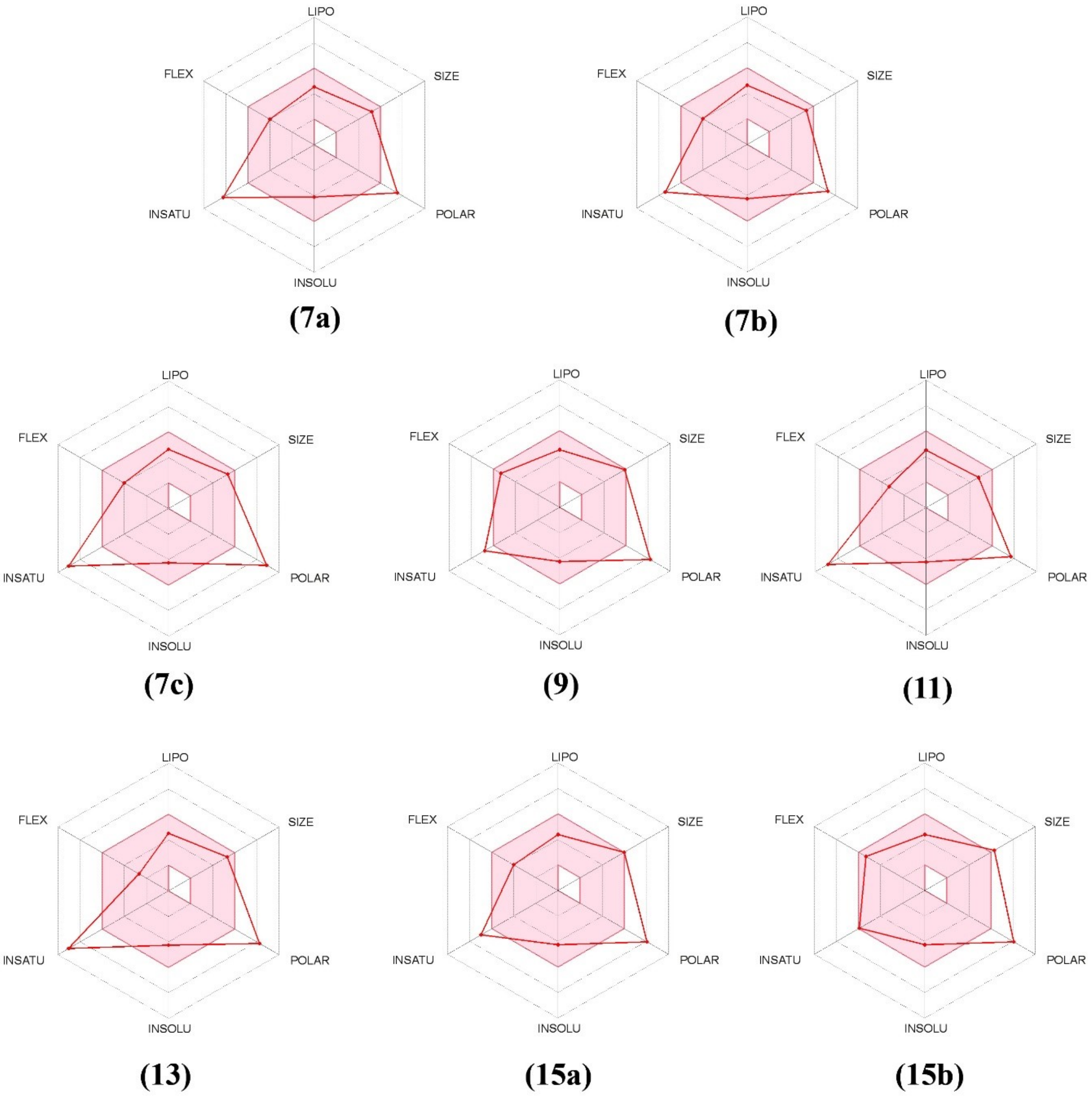

Figure S11. Swiss ADME's bioavailability radar of the synthesized compounds.

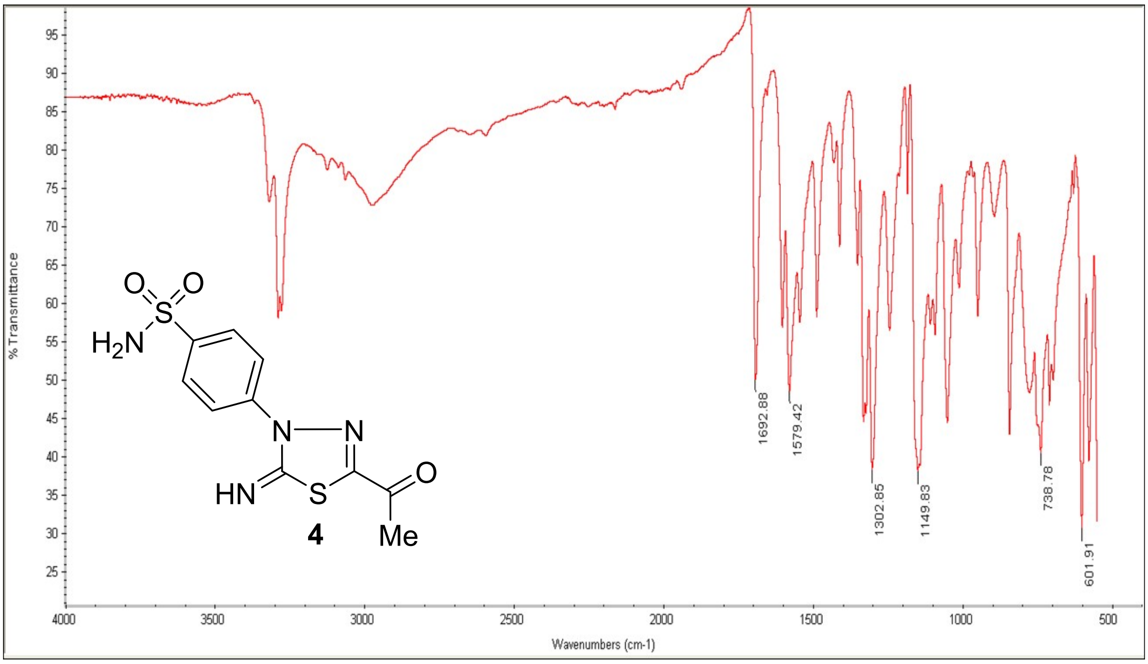

Figure S12. IR spectrum of compound **4**

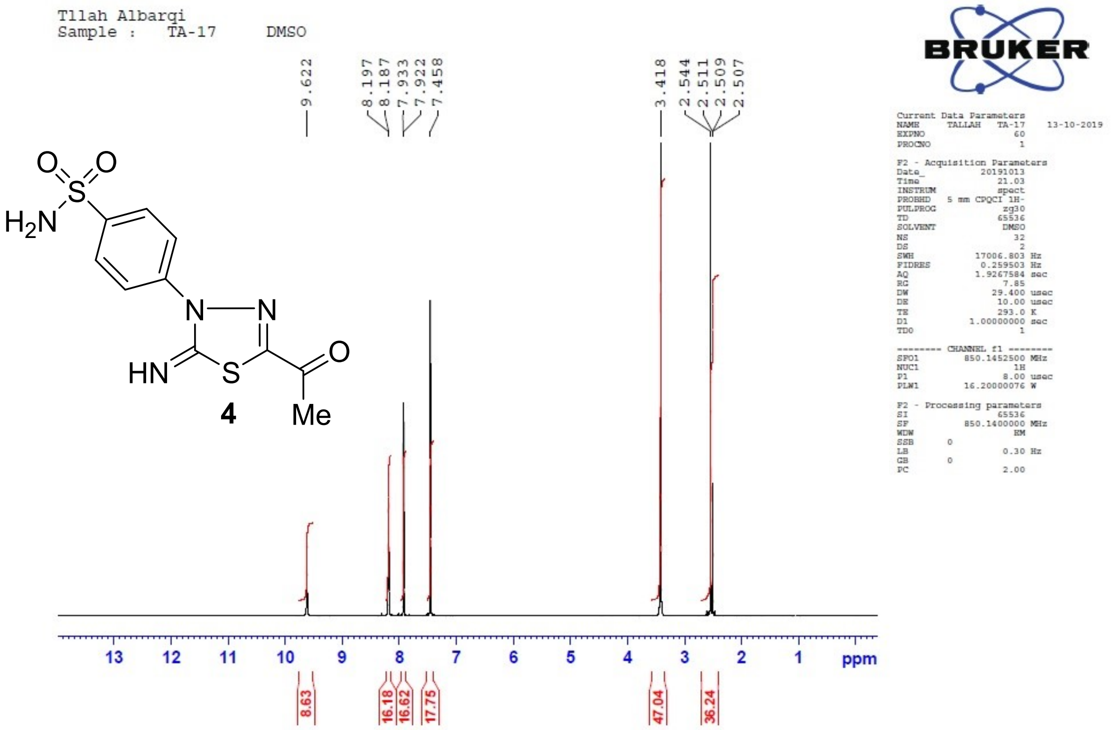

Figure S13. ^{1}H-NMR spectrum of compound **4**.

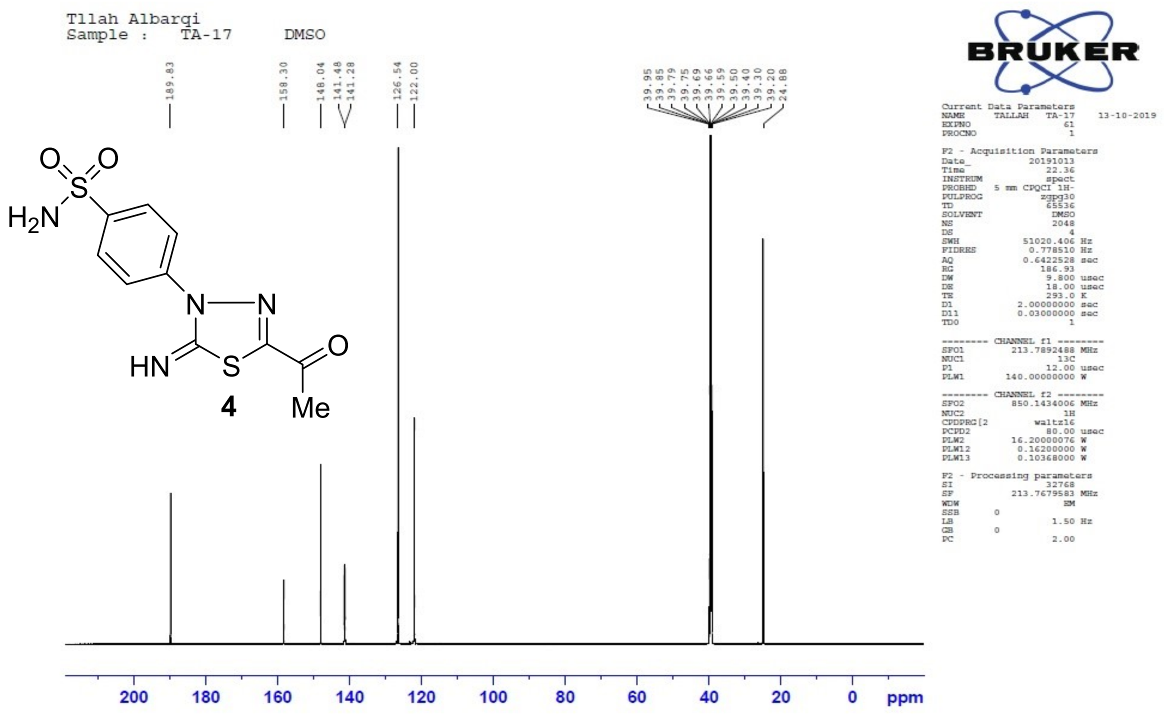

Figure S14. ^{13}C-NMR spectrum of compound **4**.

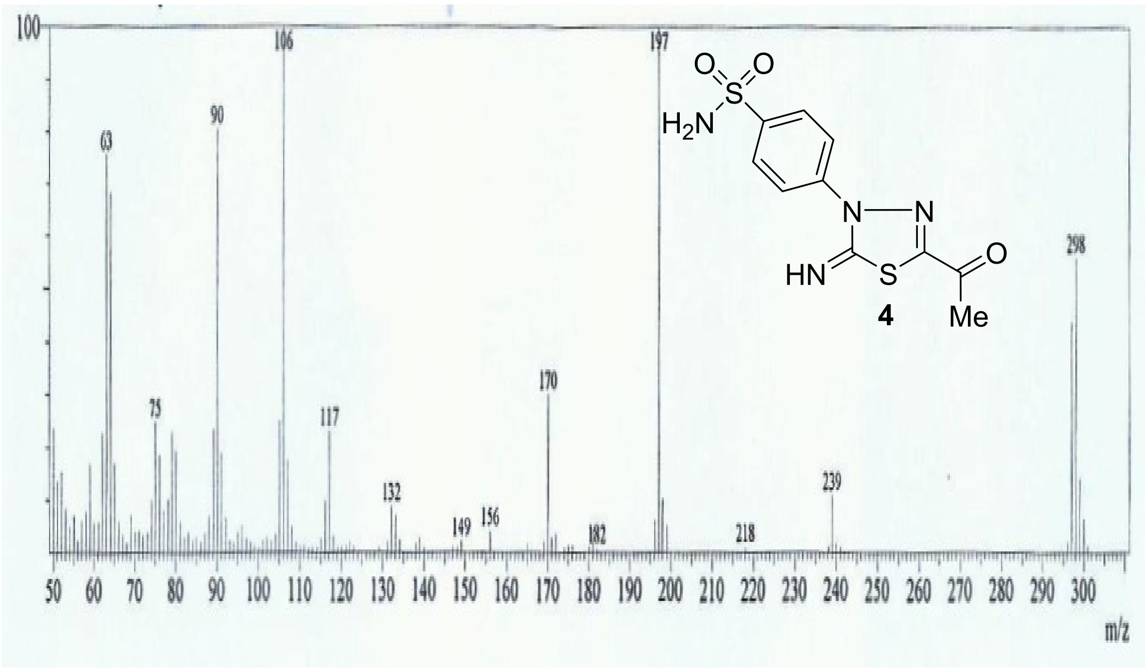

Figure S15. Mass spectrum of compound **4.**

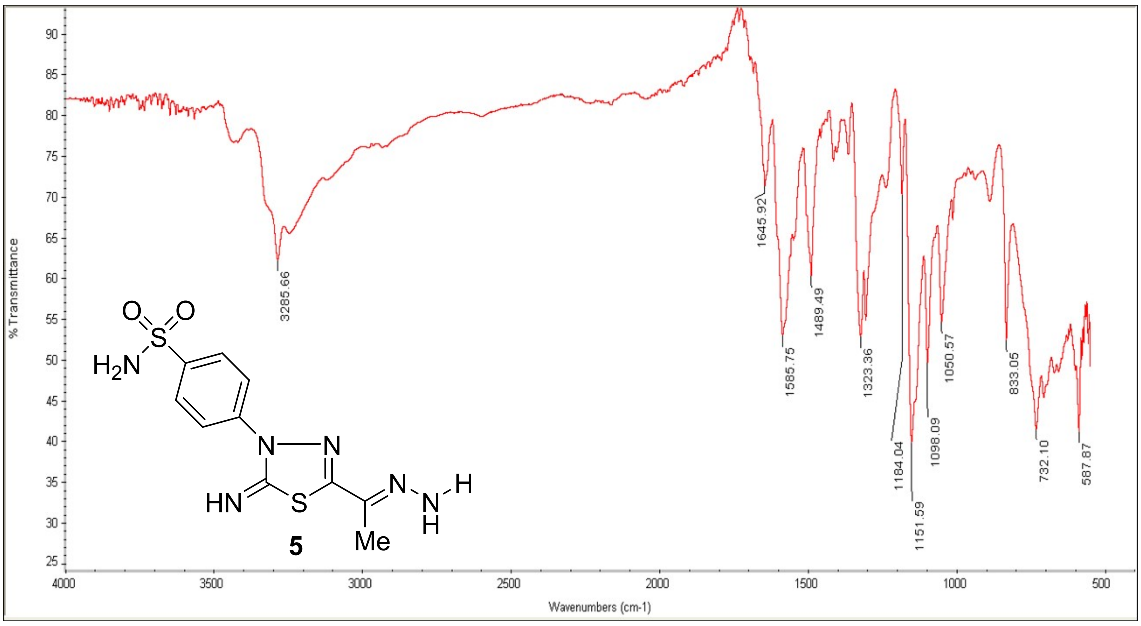

Figure S16. IR spectrum of compound **5**.

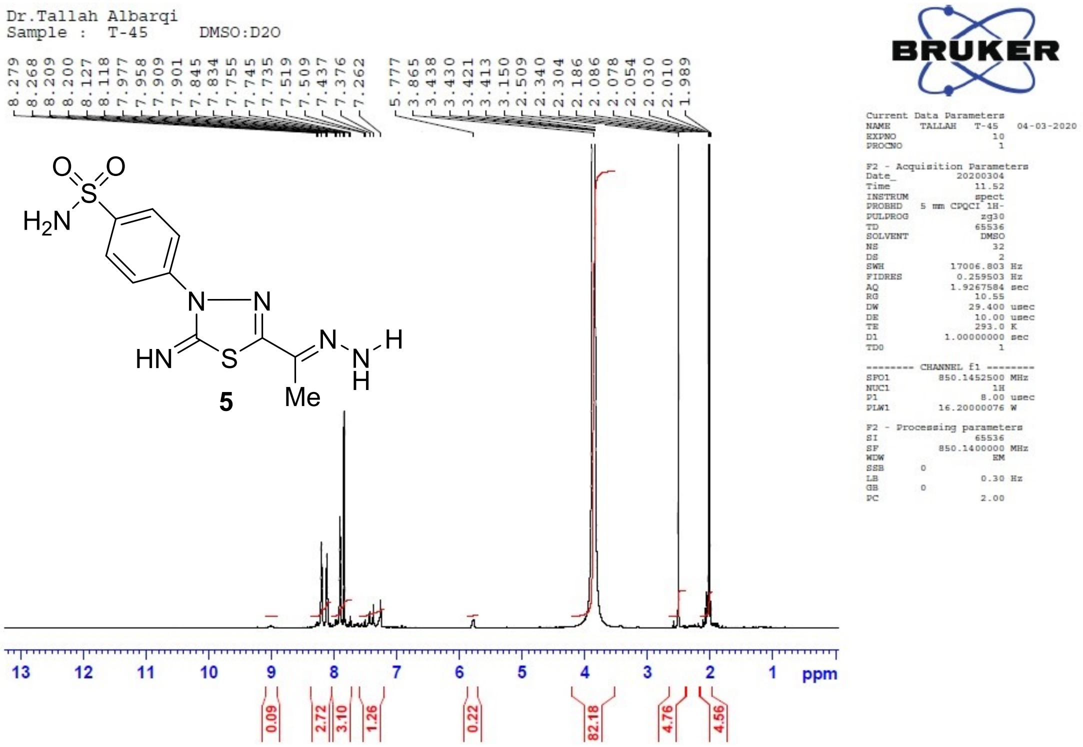

Figure S17. ^{1}H-NM spectrum of compound **5**.

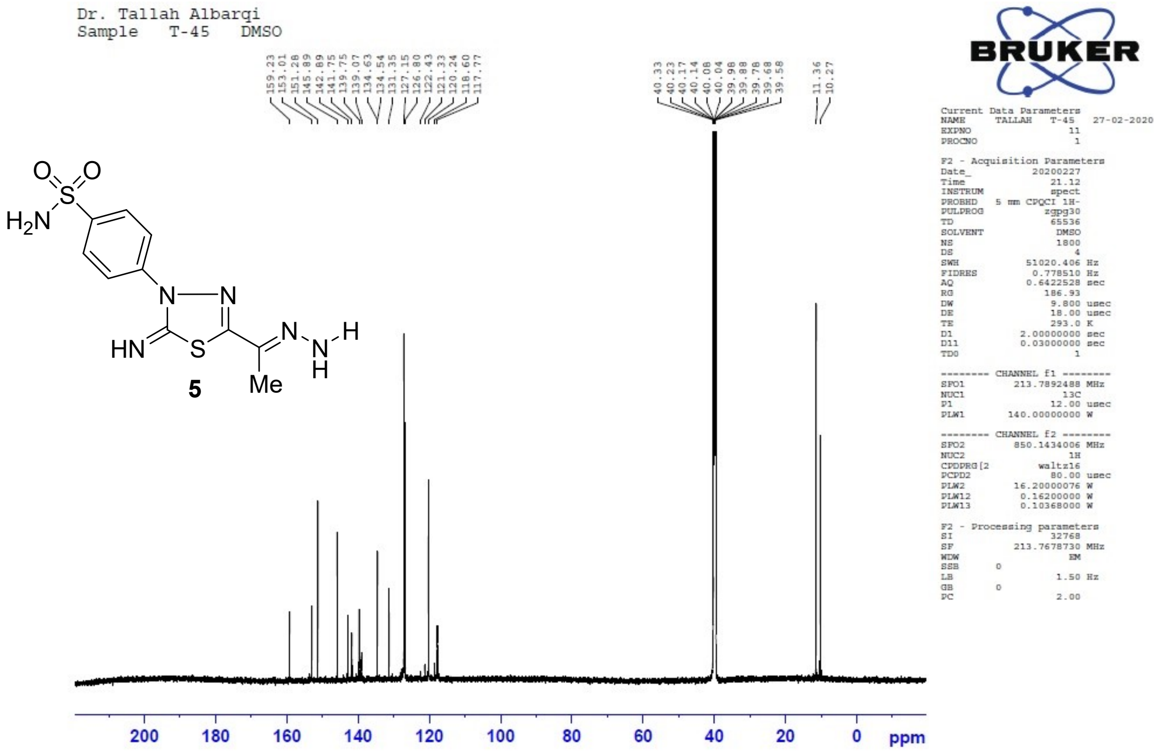

Figure S18. ^{13}C-NMR spectrum of compound **5**.

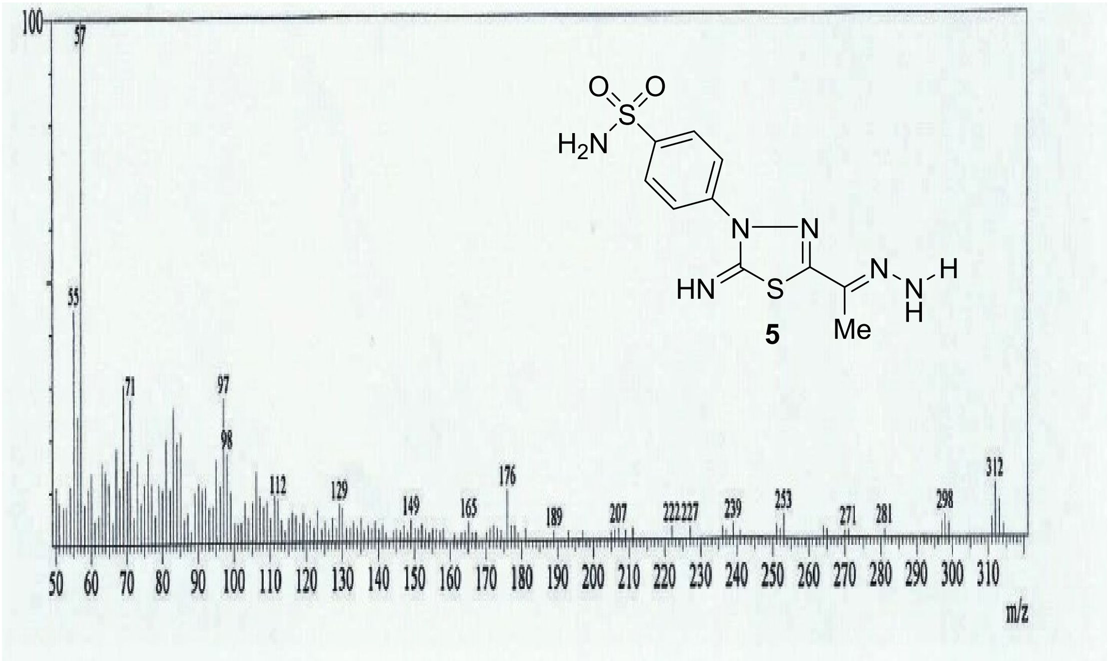

Figure S19. Mass spectrum of compound **5.**

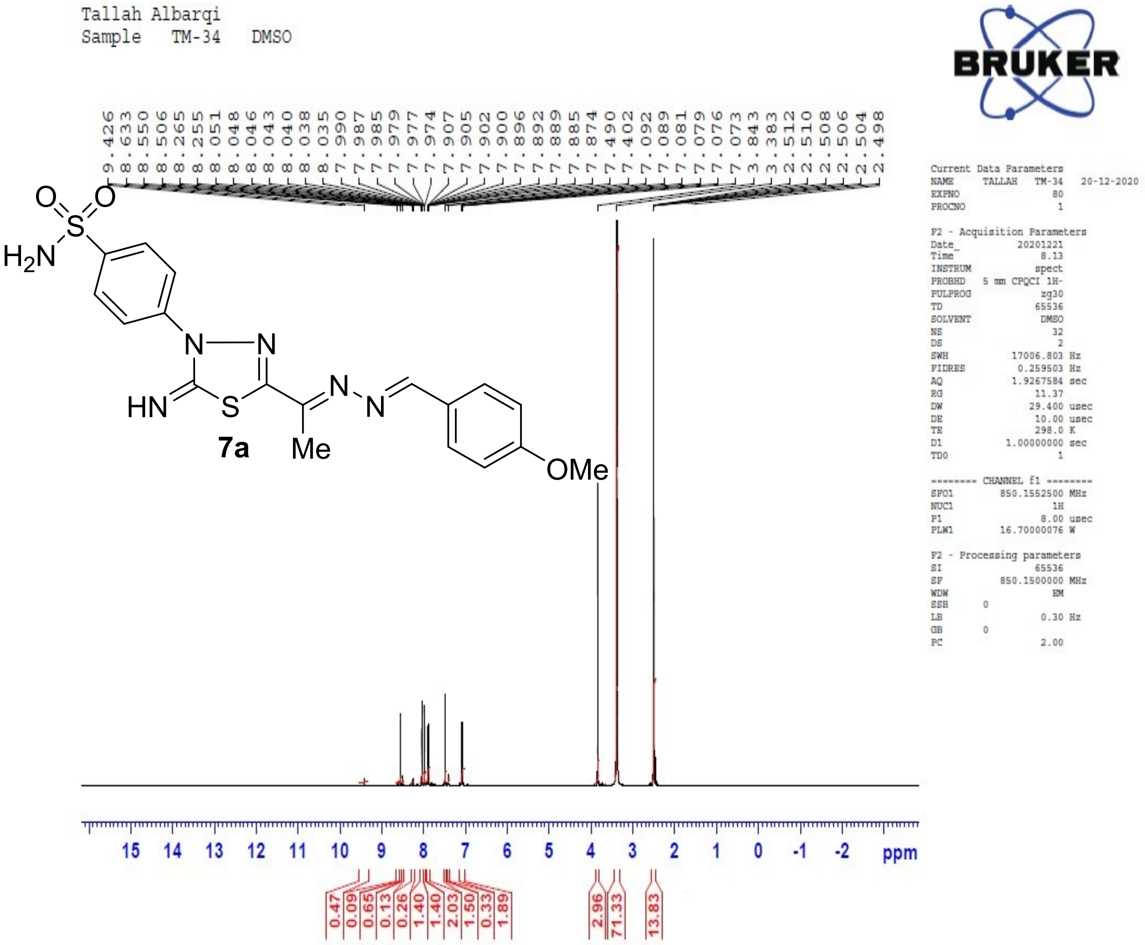

Figure S20. ^{1}H-NMR spectrum of compound **7a**.

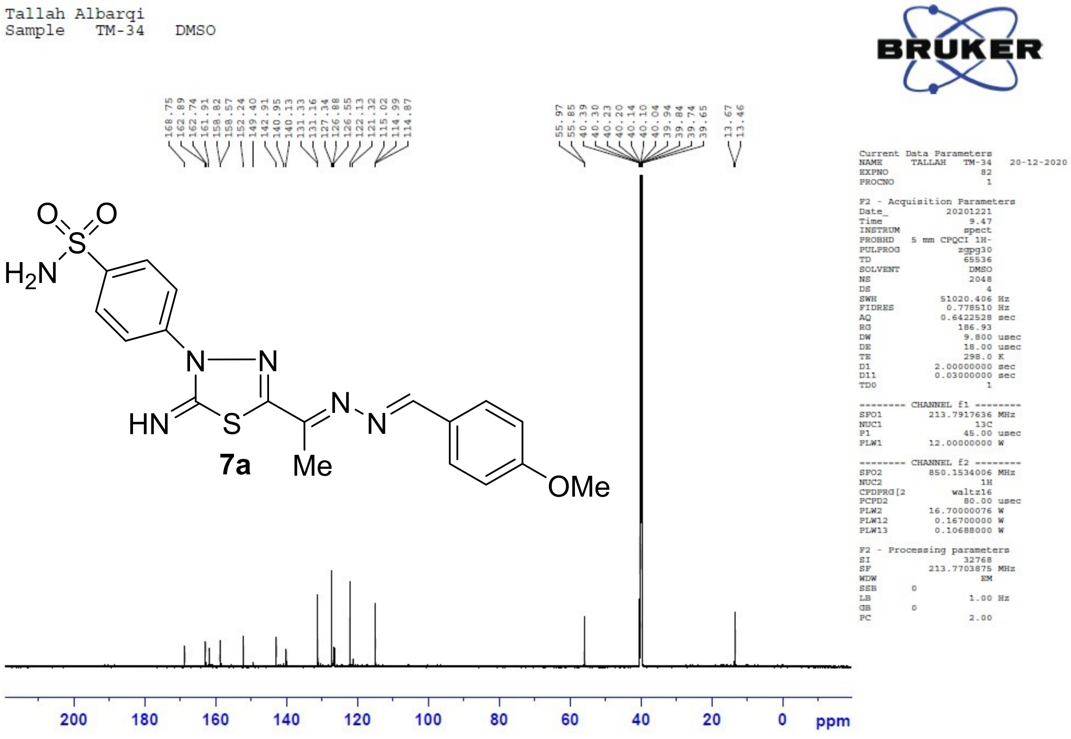

Figure S21.^{3}C-NMR spectrum of compound **7a**.

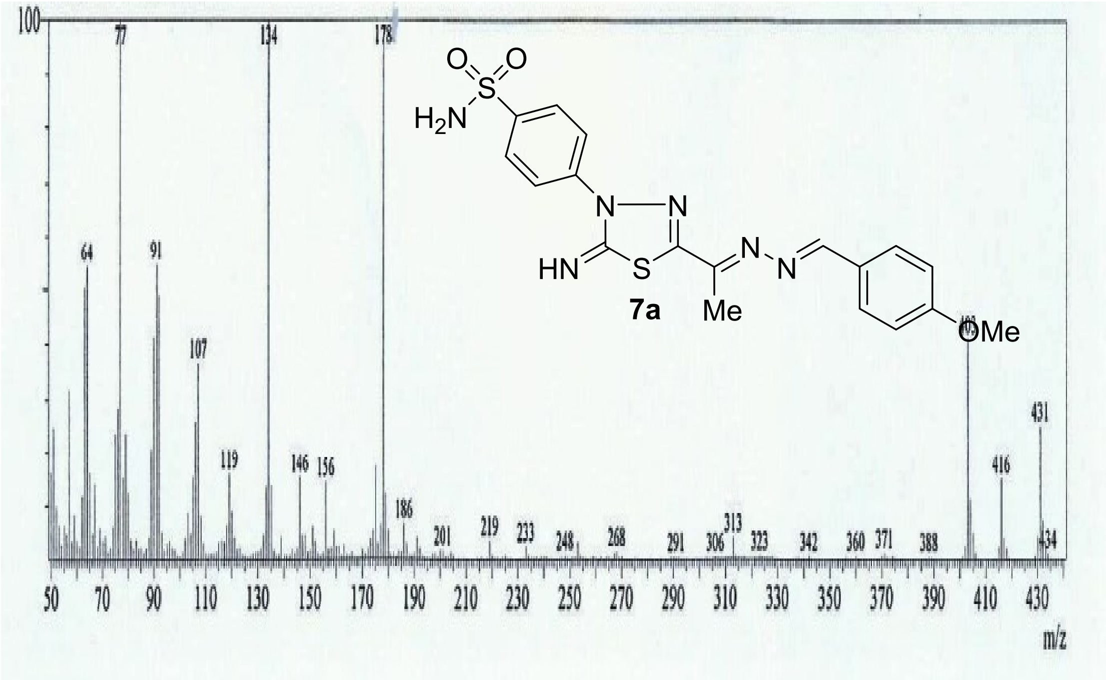

Figure S22. Mass spectrum of compound **7a.**

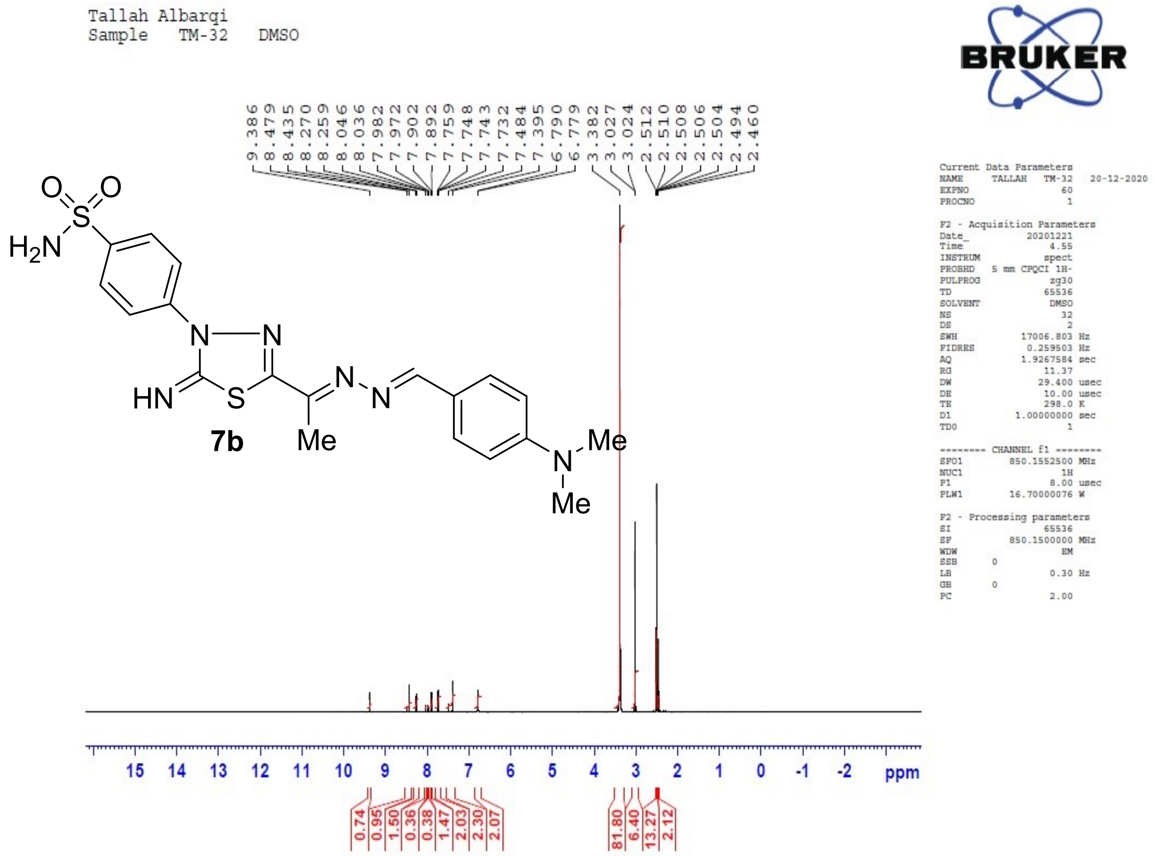

Figure S23. ^{1}H-NMR spectrum of compound **7b**.

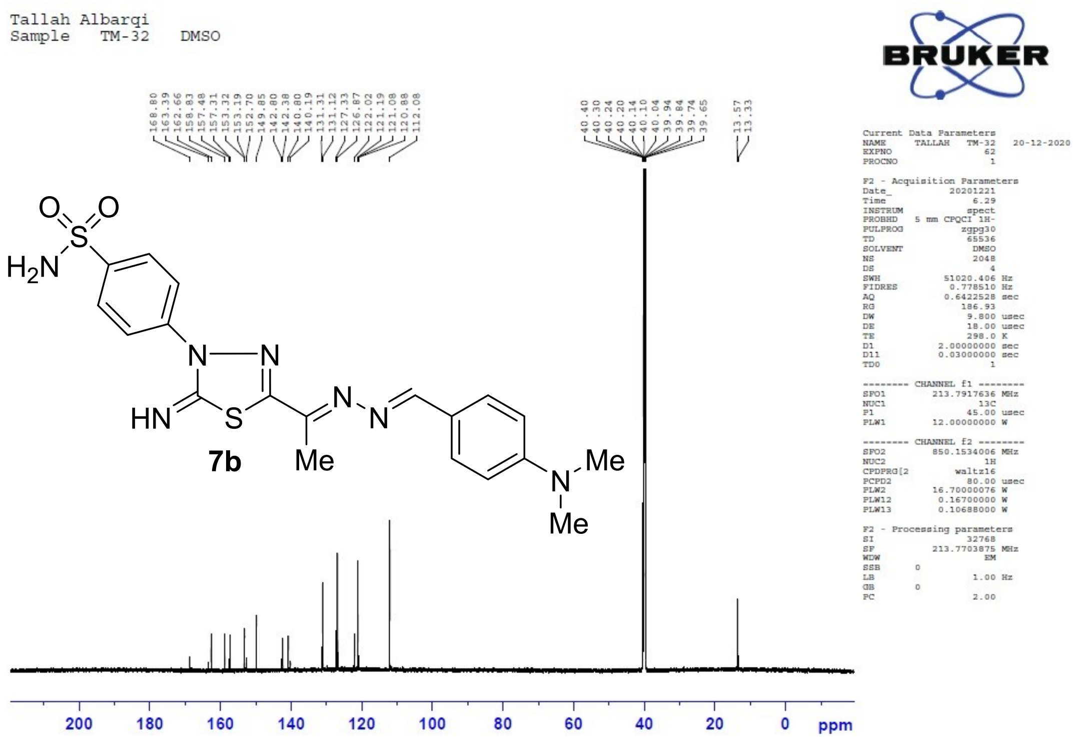

Figure S24. ^{13}C-NMR spectrum of compound **7b**.

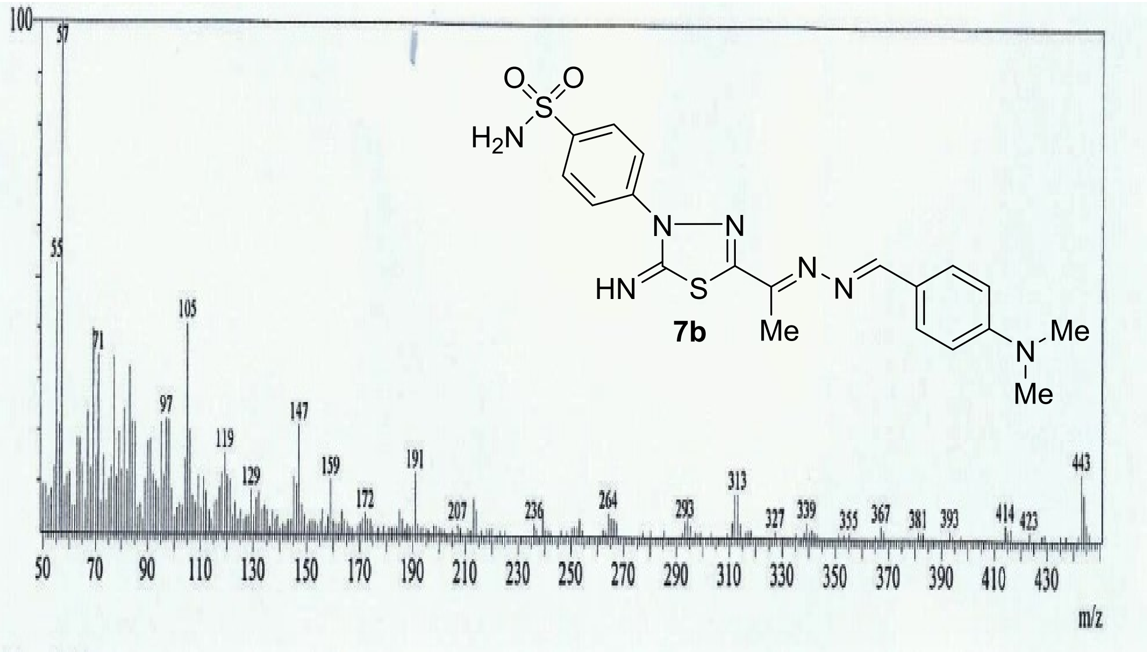

Figure S25. Mass spectrum of compound **7b.**

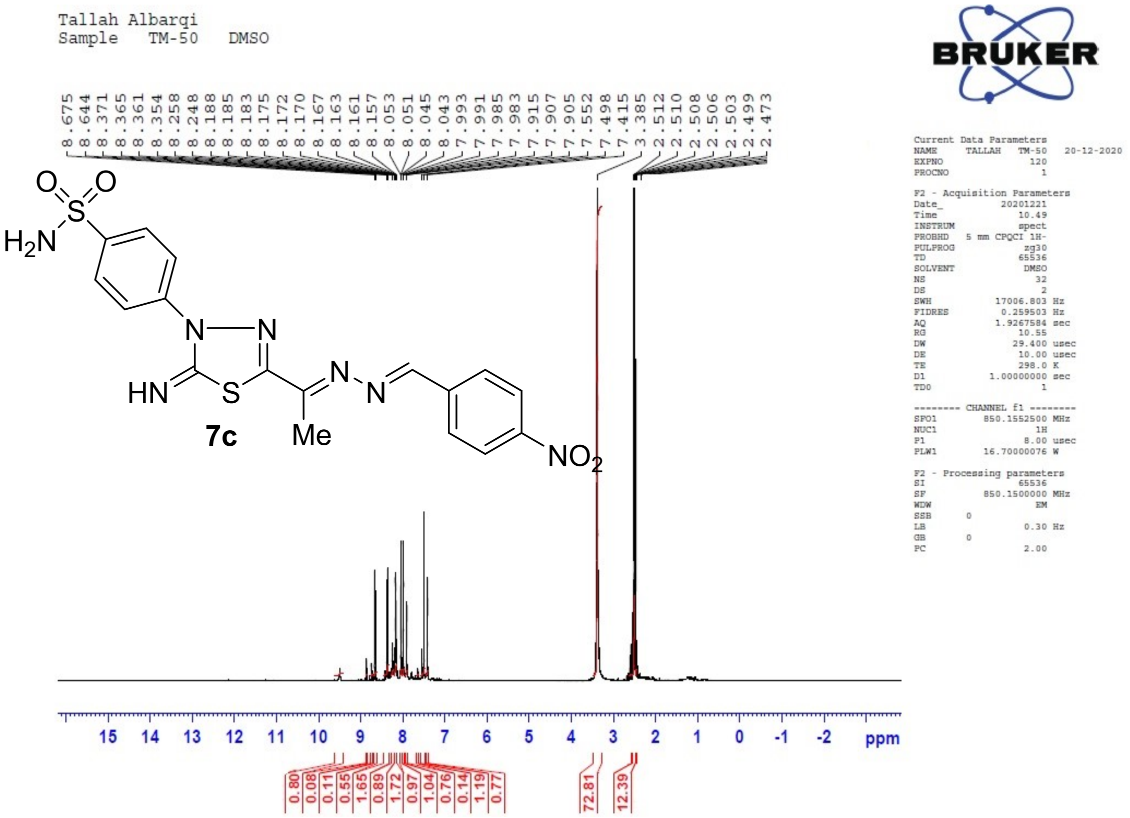

Figure S26. ^{1}H-NMR spectrum of compound **7c.**

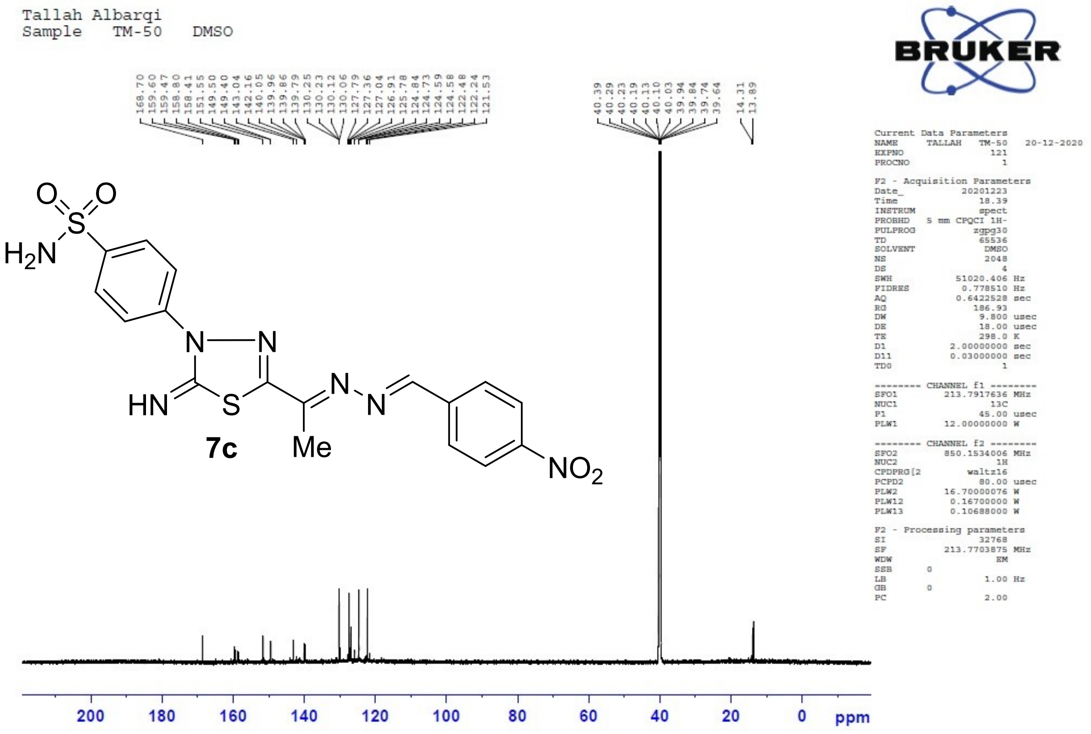

Figure S27. ^{13}C-NMR spectrum of compound **7c**.

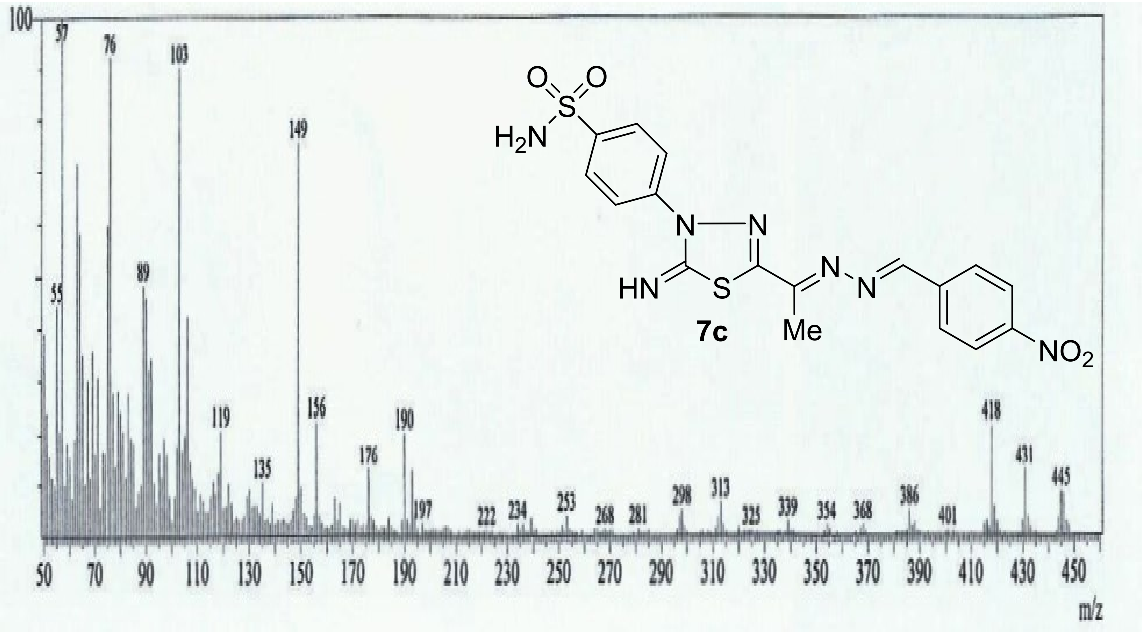

Figure S28. Mass spectrum of compound **7c.**

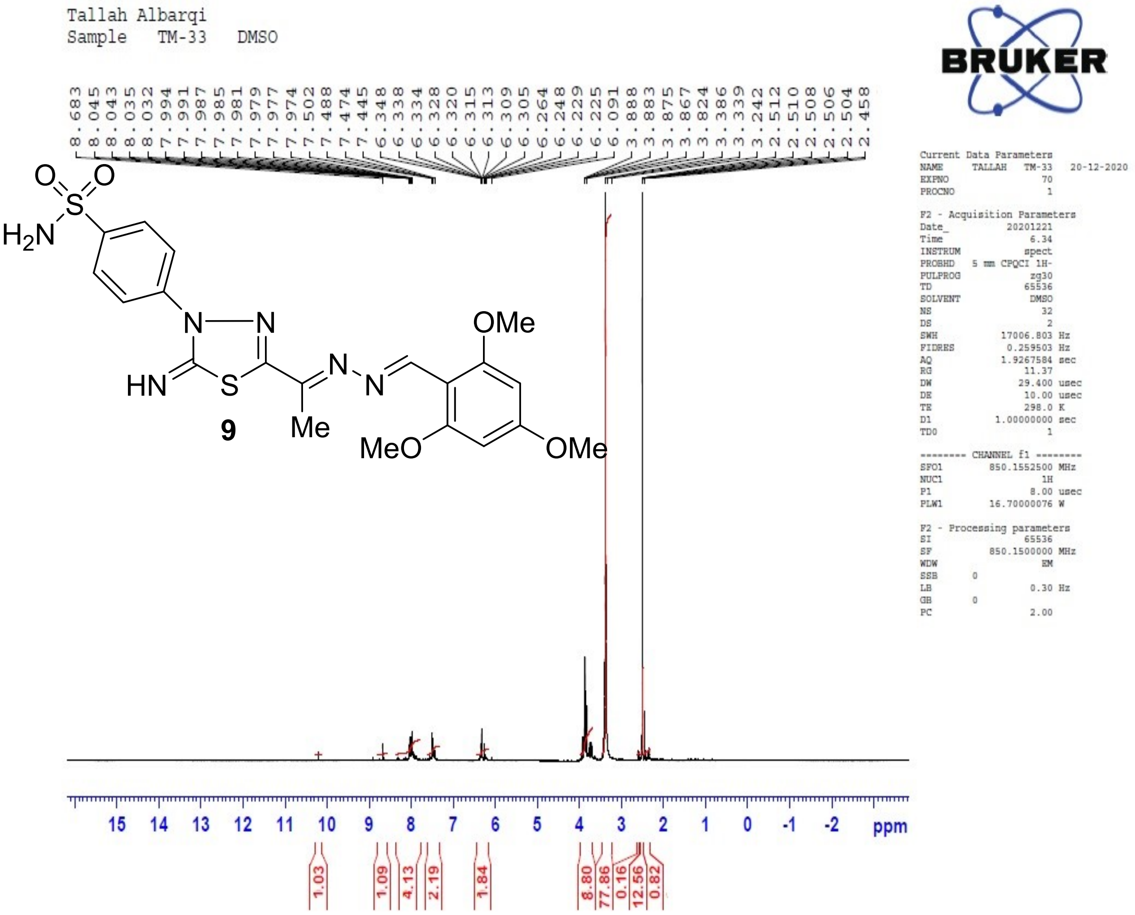

Figure S29. ^{1}H-NMR spectrum of compound **9**.

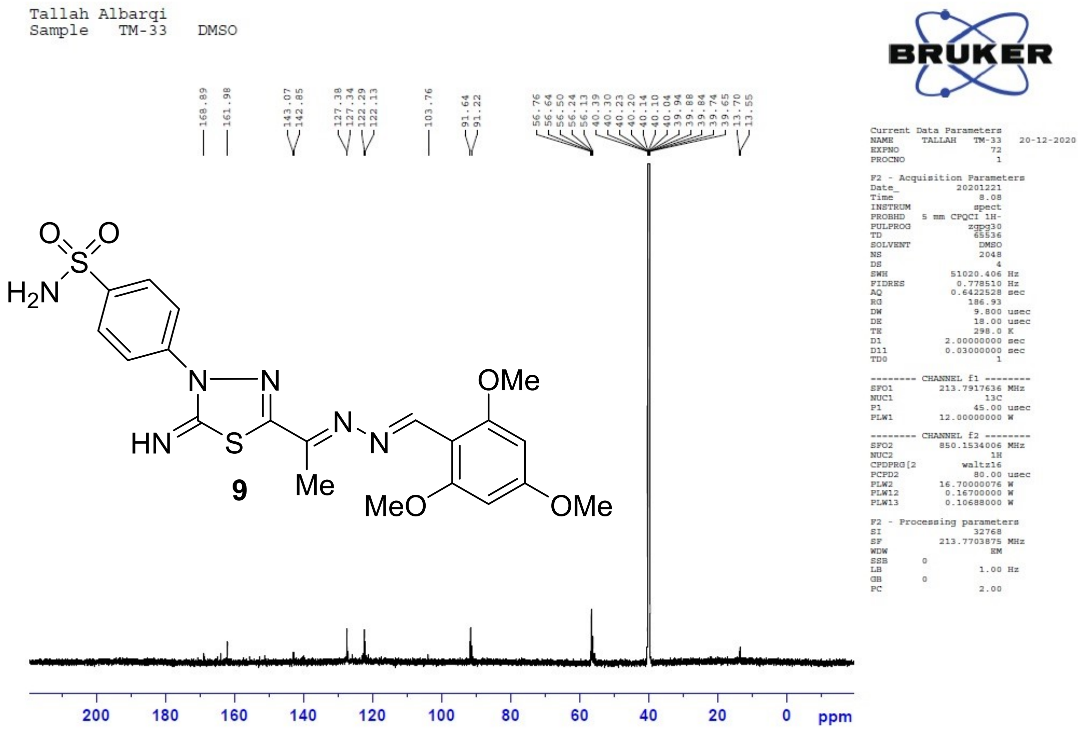

Figure S30. ^{13}C-NMR spectrum of compound **9**.

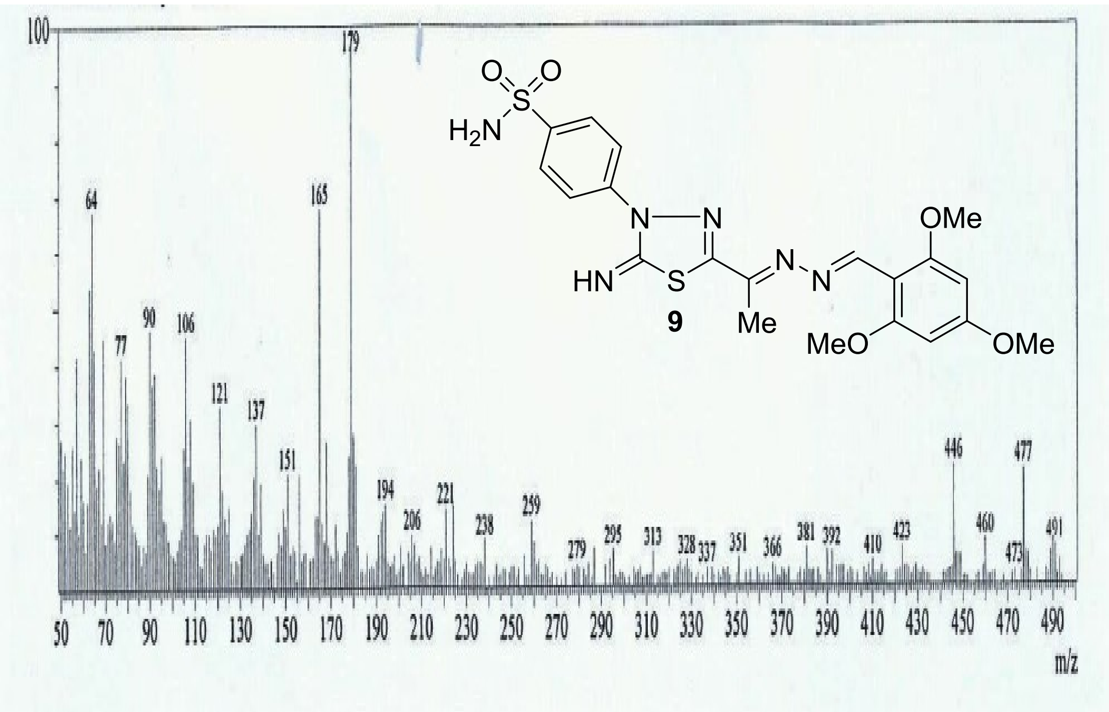

Figure S31. Mass spectrum of compound **9.**

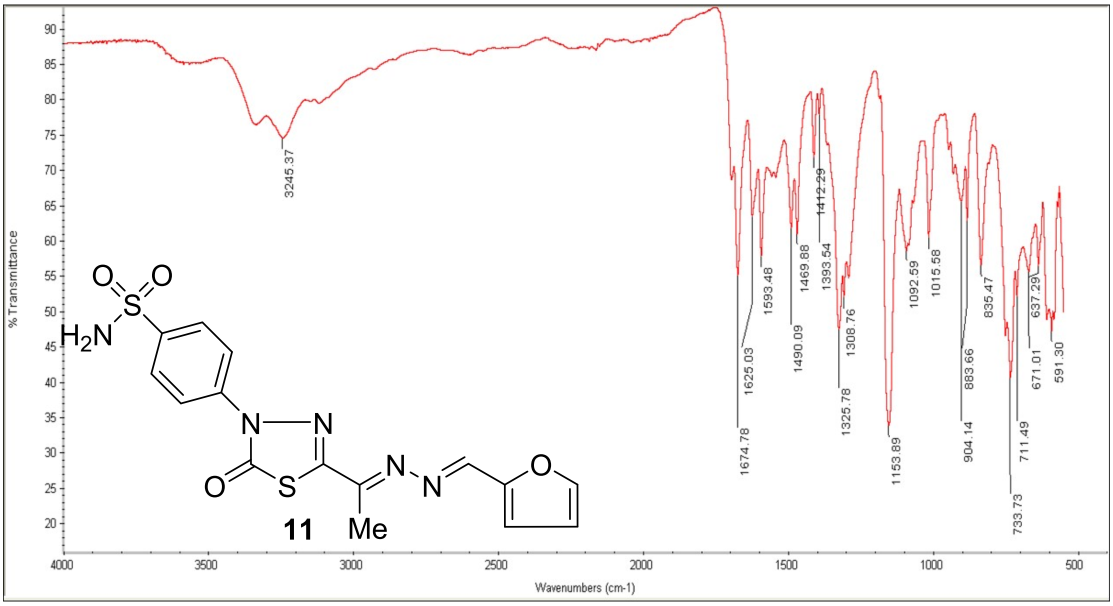

Figure S32. IR spectrum of compound **11.**

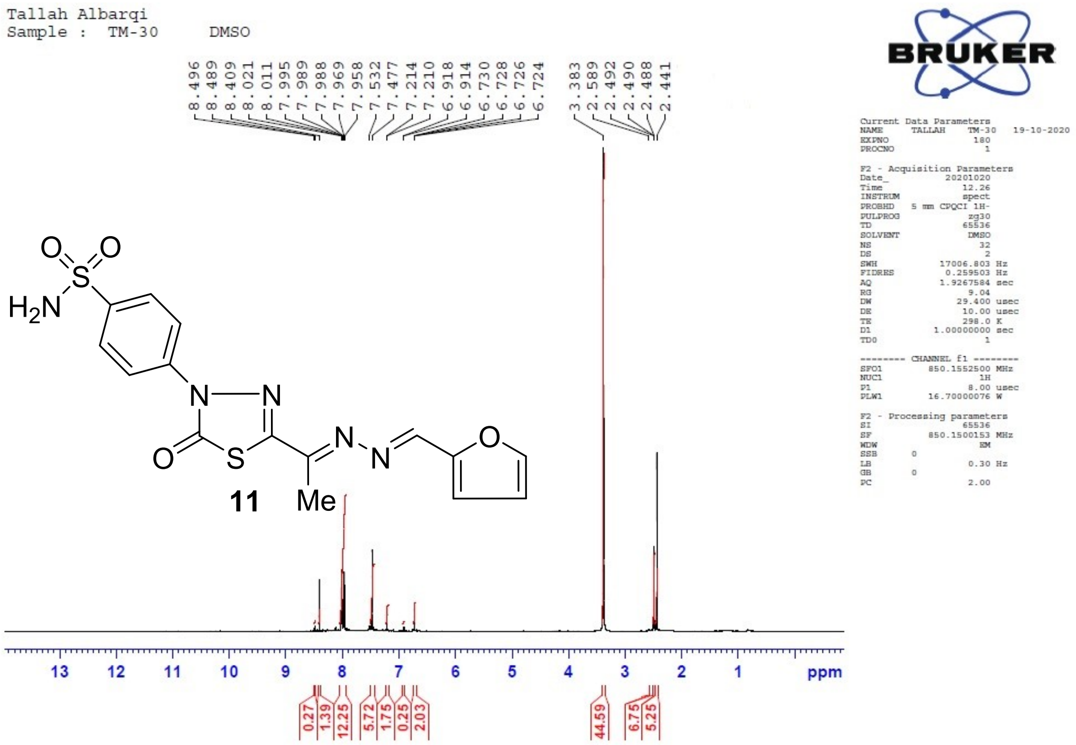

Figure S33. ^{1}H-NMR spectrum of compound **11.**

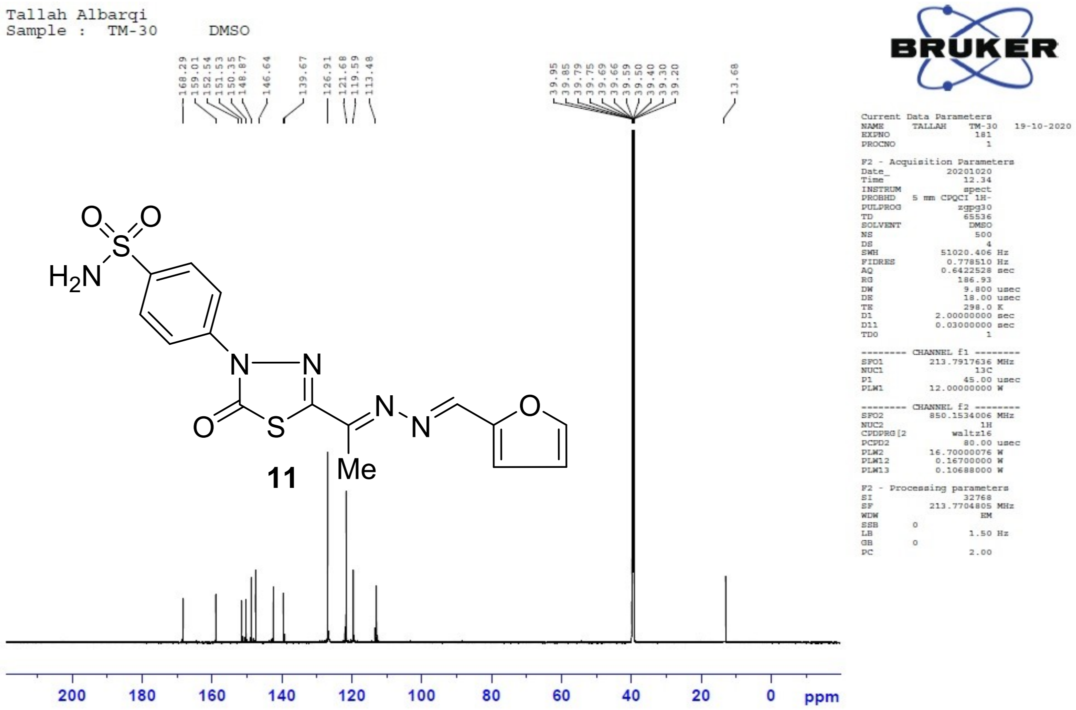

Figure S34. ^{13}C-NMR spectrum of compound **11**.

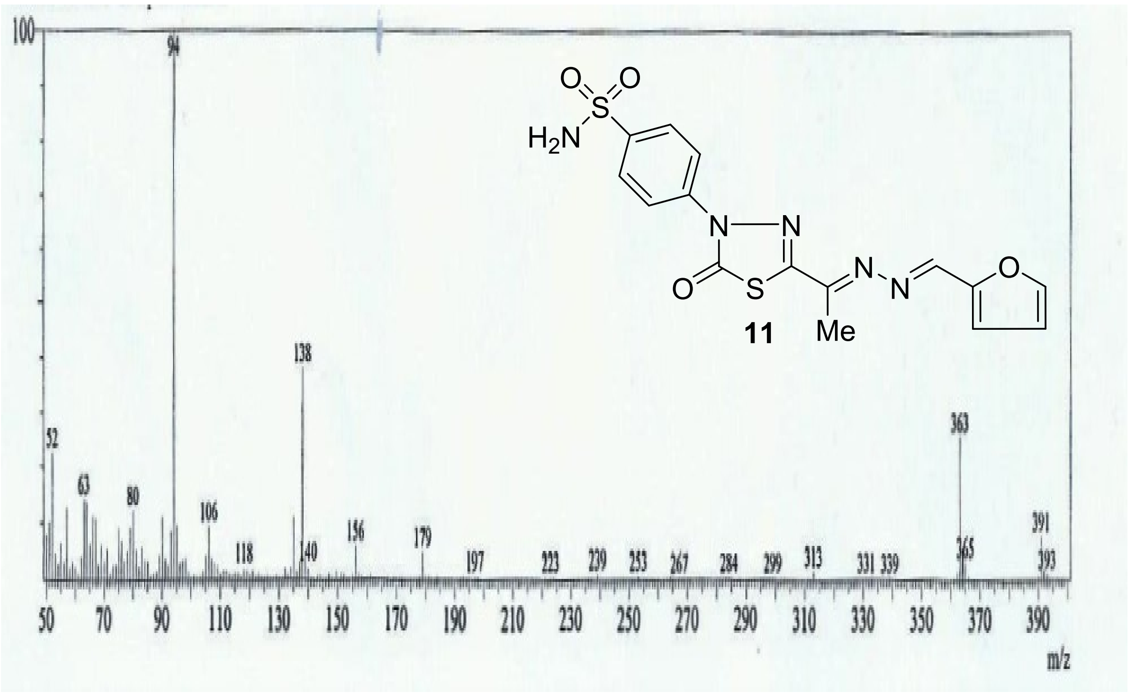

Figure S35. Mass spectrum of compound **11.**

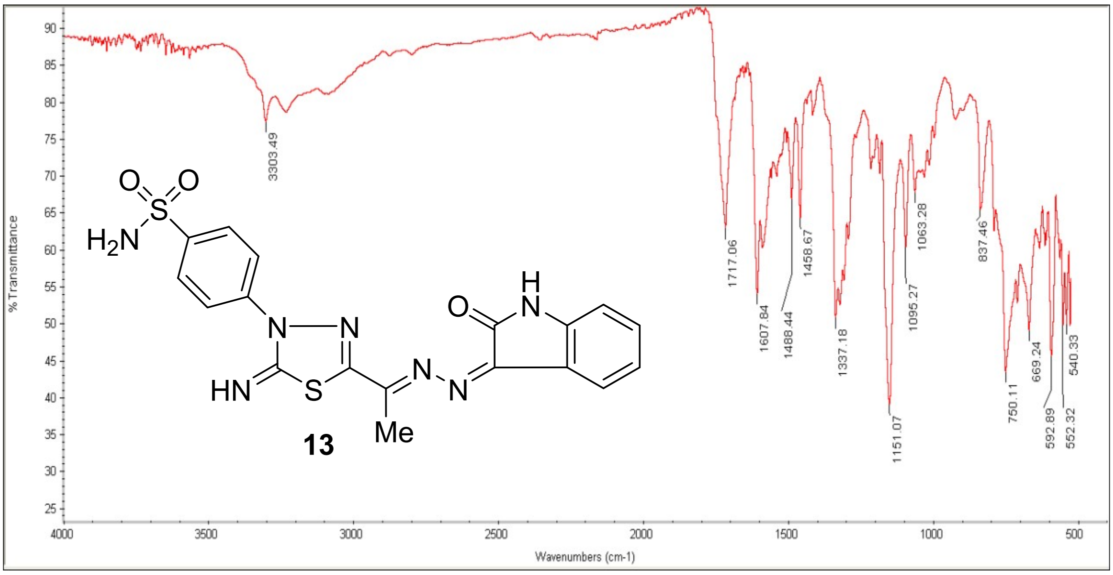

Figure S36. IR spectrum of compound **13.**

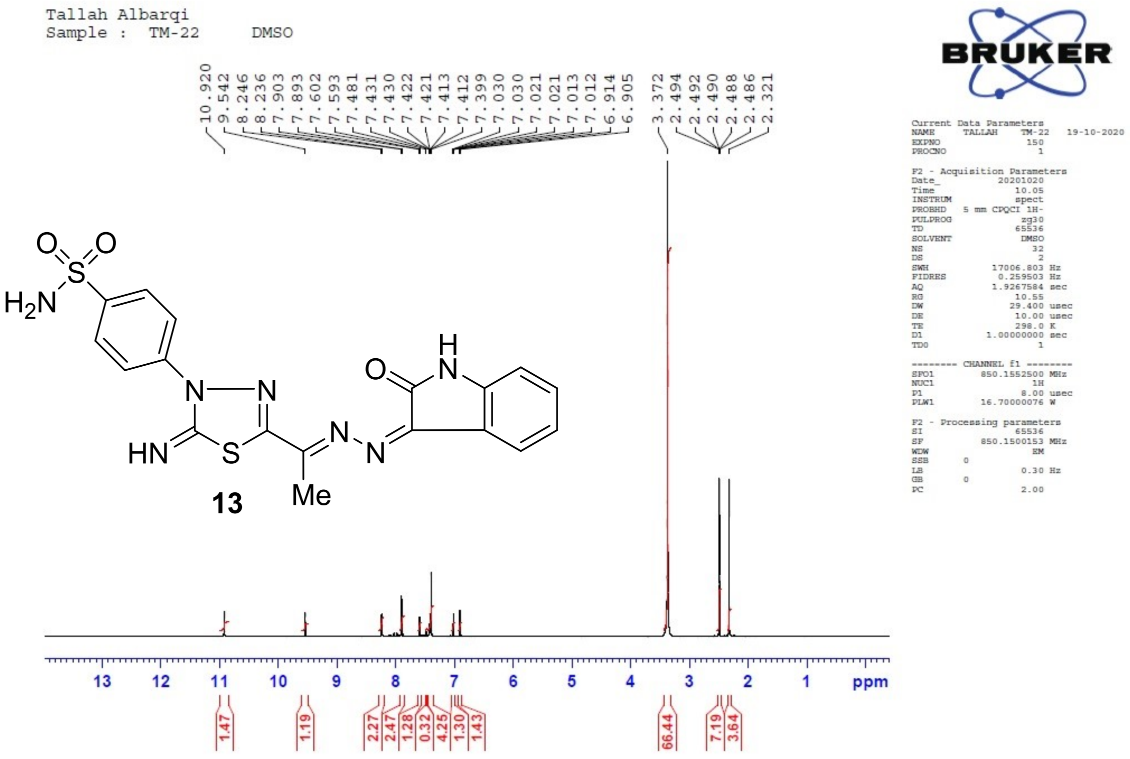

Figure S37. ^{1}H-NMR spectrum of compound **13.**

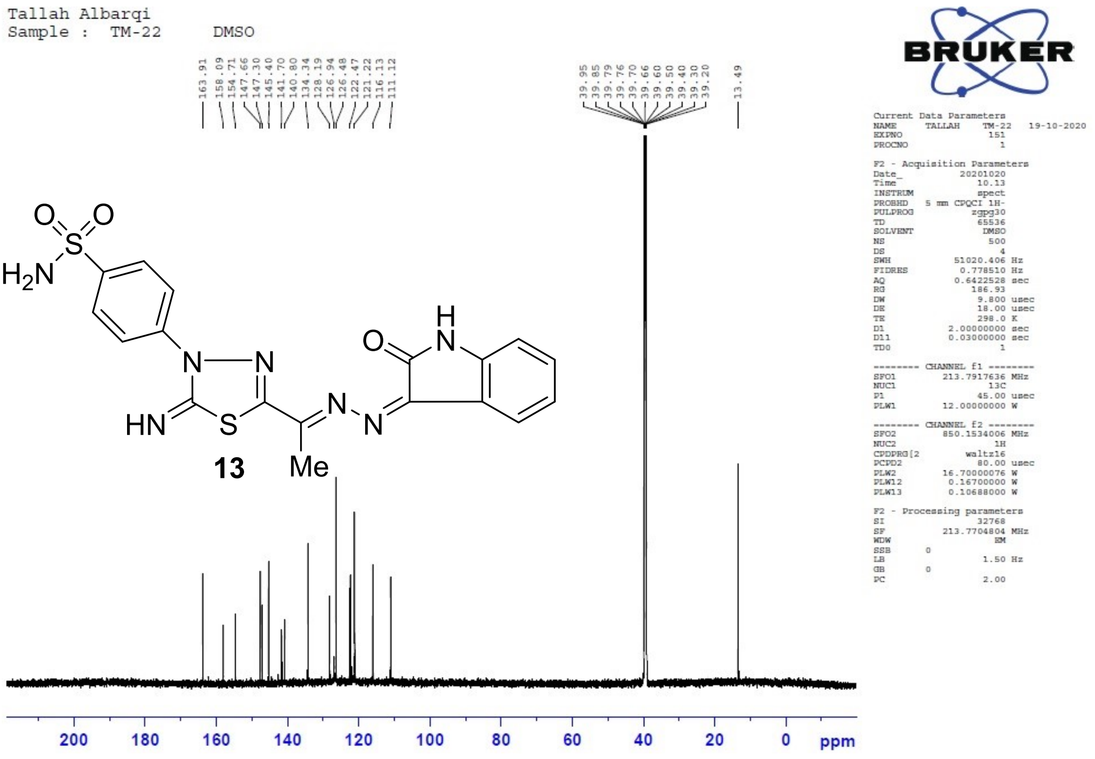

Figure S38. ^{13}C-NMR spectrum of compound **13.**

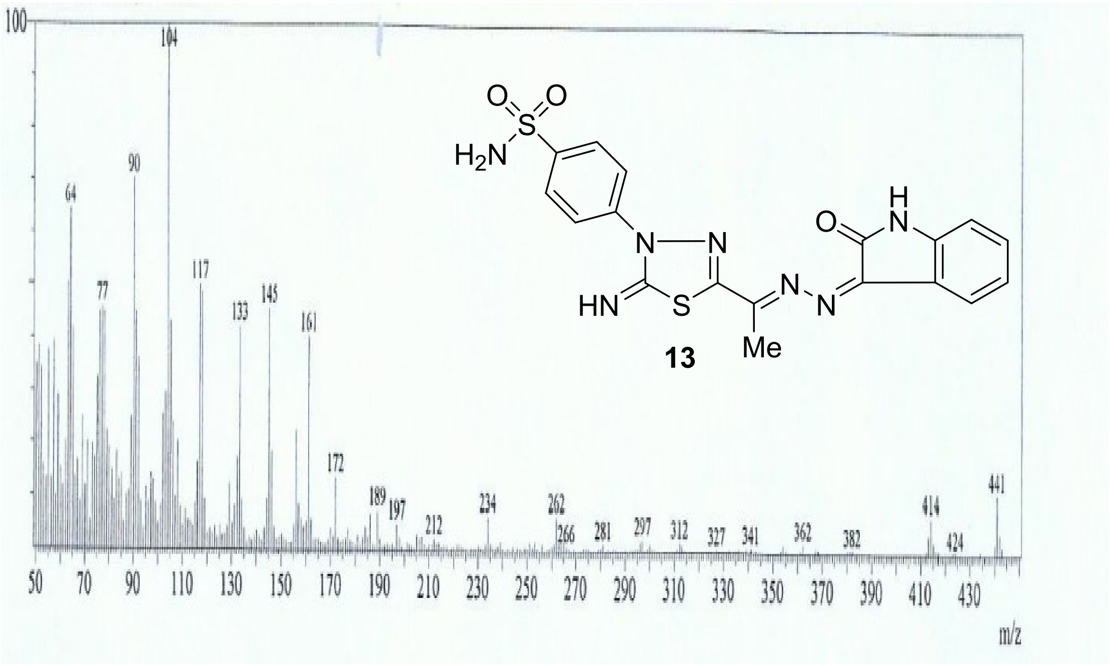

Figure S39. Mass spectrum of compound **13.**

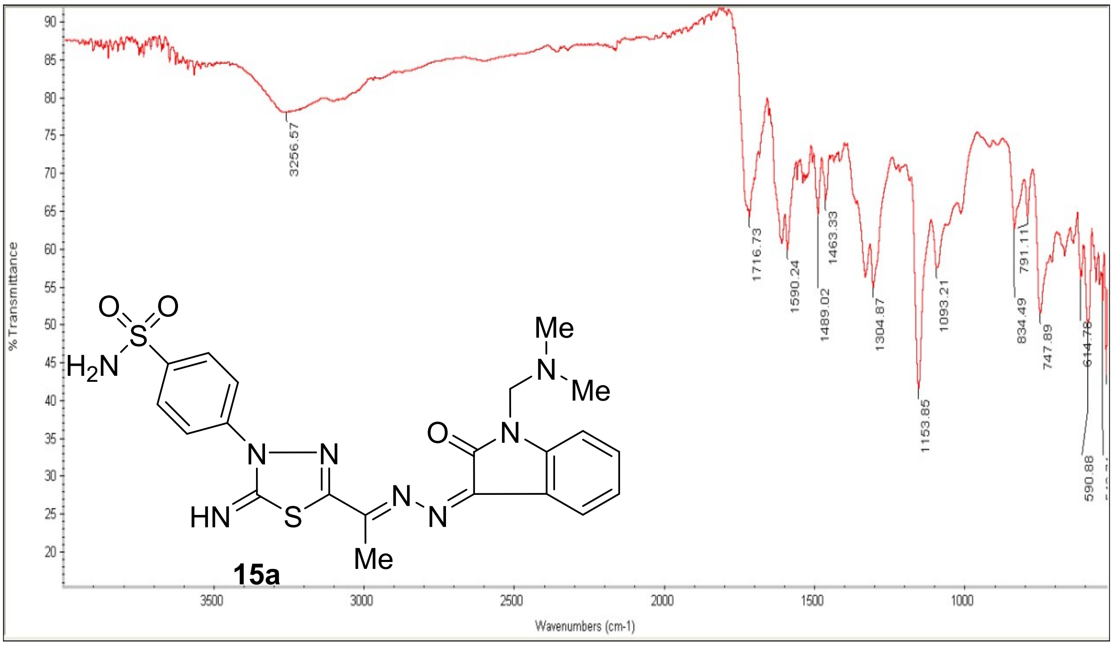

Figure S40. IR spectrum of compound **15a.**

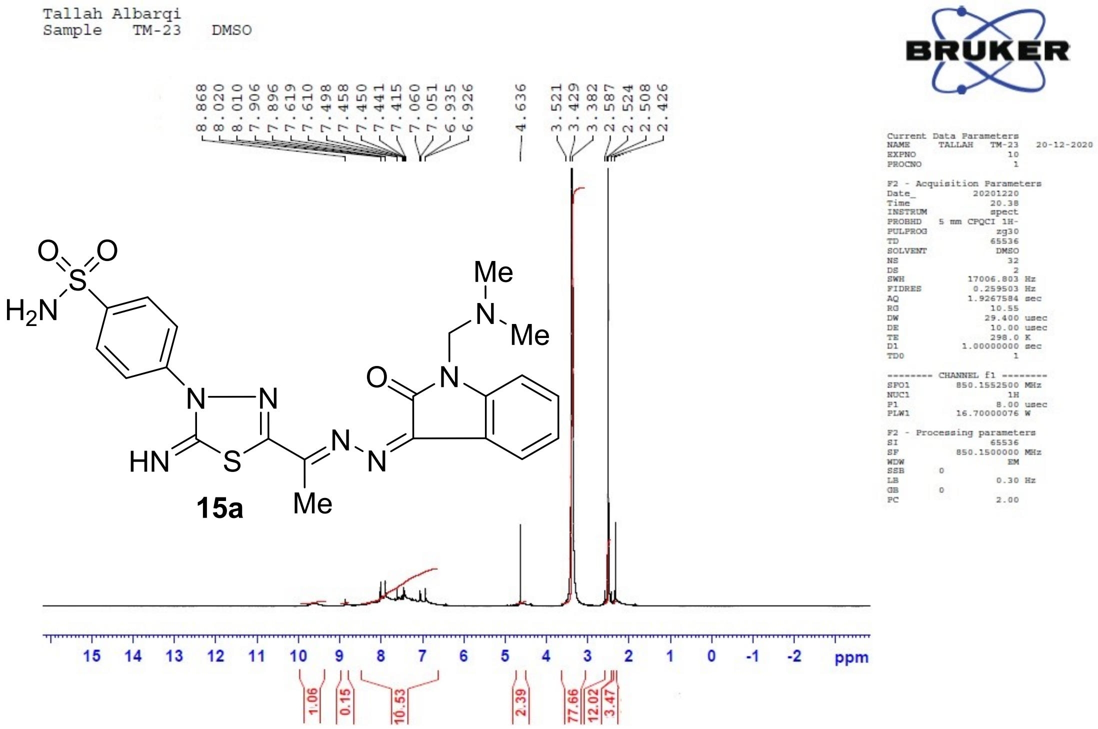

Figure S41. ^{1}H-NMR spectrum of compound **15a**.

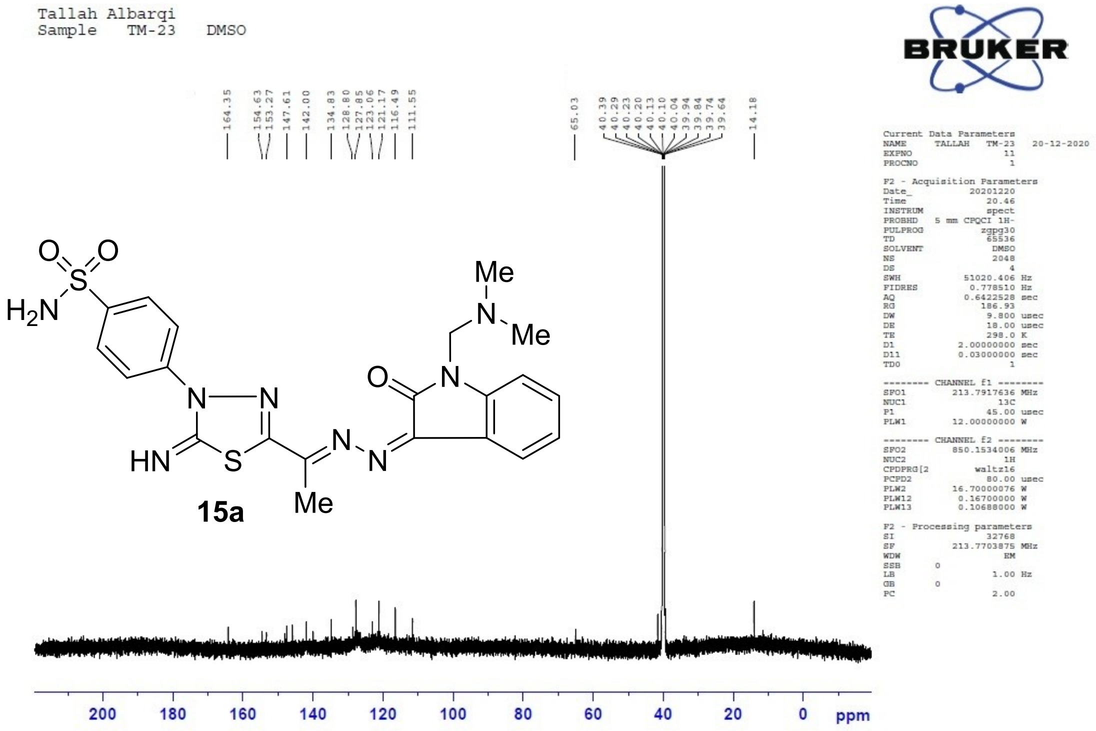

Figure S42. ^{13}C-NMR spectrum of compound **15a**.

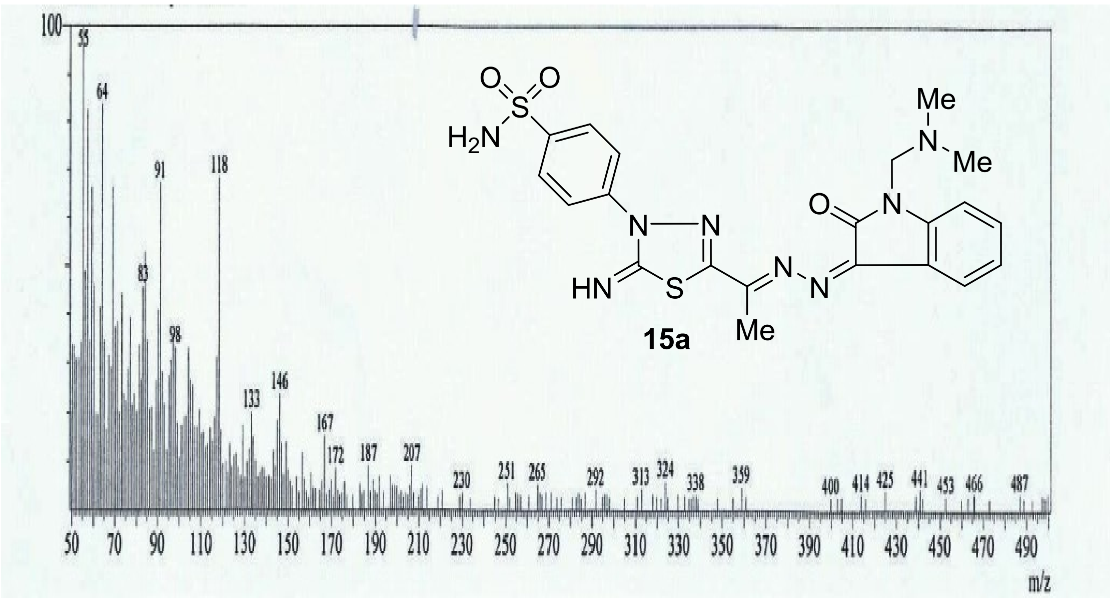

Figure S43. Mass spectrum of compound **15a.**

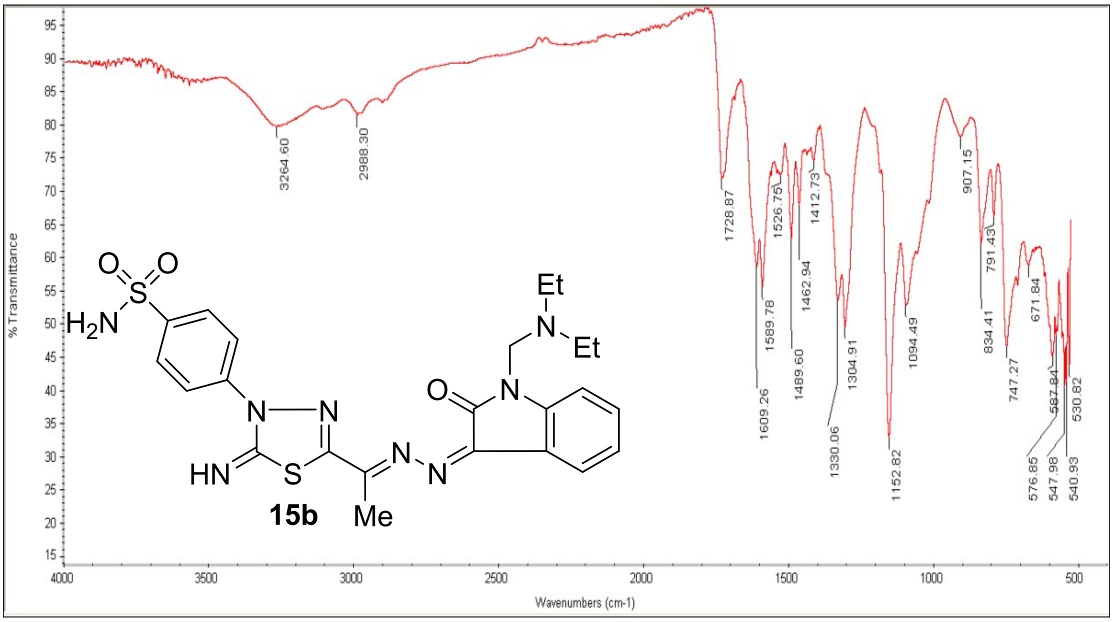

Figure S44. IR spectrum of compound **15b**.

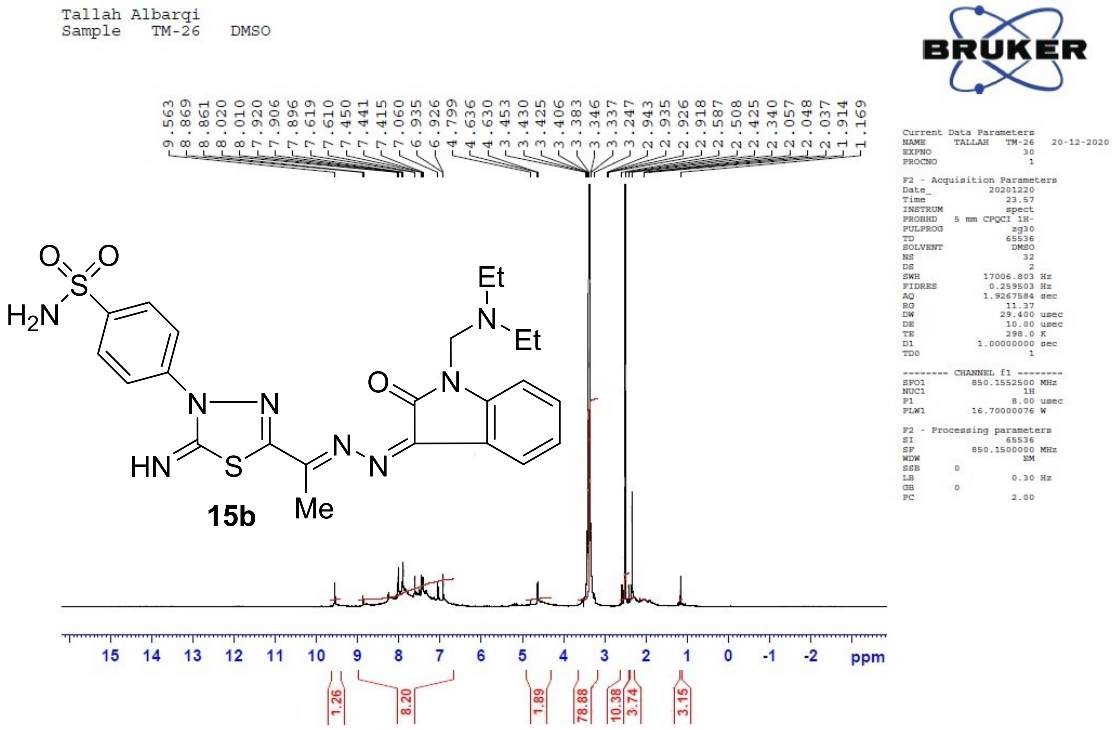

Figure S45. ^{1}H-NMR spectrum of compound **15b**.

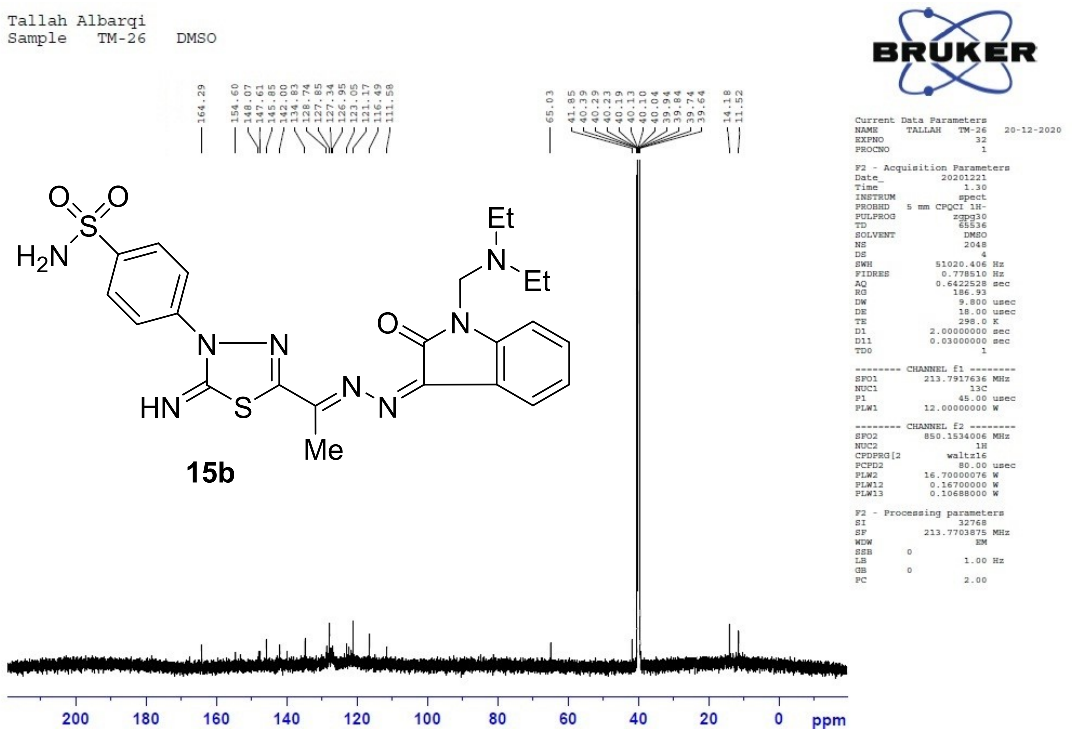

Figure S46. ^{13}C-NMR spectrum of compound **15b**.

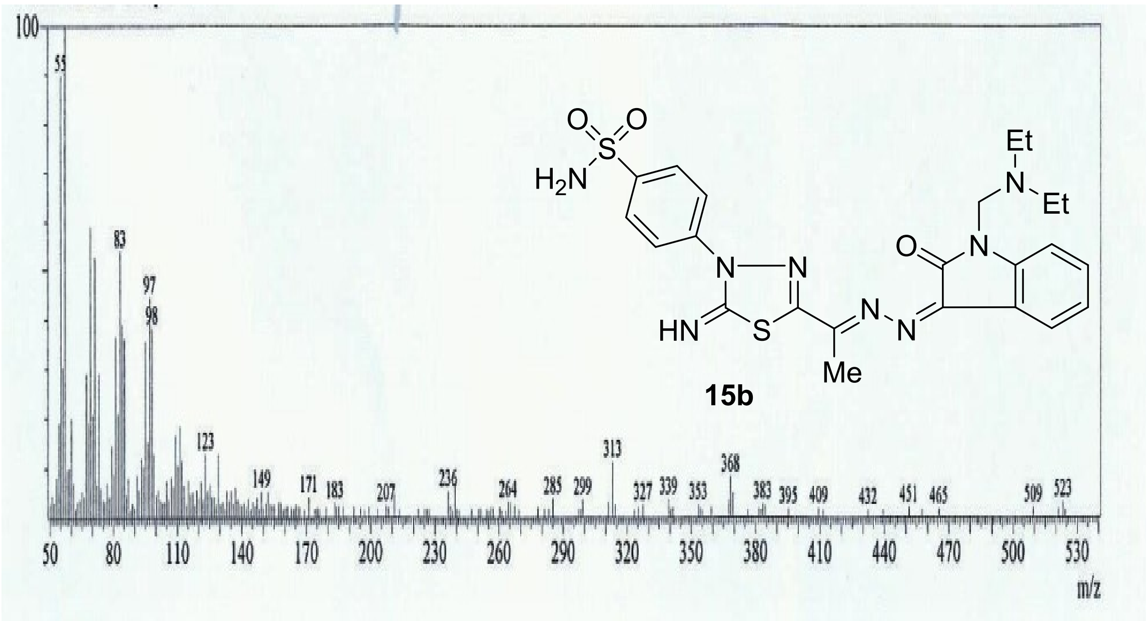

Figure S47. Mass spectrum of compound **15b.**

Table S1 Calculated[a] energies (E) and energy differences (ΔE) for possible configurations of the synthesized products.

Cpd	E (Hartree)	ΔE (kcal/mol) [b]		
	**1 (EE)** [c]	**2 (ZZ)** [c]	**3 (EZ)** [c]	**4 (ZE)** [c]
**7a**	– 2047.174609	5.27	3.83	3.81
**7b**	– 2066.621509	5.12	3.81	1.74
**7c**	– 2137.148645	5.60	3.74	2.06
**9**	– 2276.213429	5.75	3.51	1.77
**11**	– 1930.42554	2.68	1.42	1.60
**13**	– 2100.141412	3.28	0.30	0.79
**15a**	– 2273.419471	3.26	0.24	0.73
**15b**	– 2352.04409	3.29	0.27	0.75

[a] The DFT-B3LYP functional combined with 6-31G(d) basis set were applied in these calculations.

[b] Energy differences (ΔE) are relative to the most stable configuration (**1**, EE),

[c] The optimized geometries of the proposed configurations for all synthesized compounds **7a-c**, **9**, **11**, **13, 15a** and **15b** (Figures S3-S10 at supplementary material).
